# The E Block motif is associated with *Legionella pneumophila* translocated substrates

**DOI:** 10.1111/j.1462-5822.2010.01531.x

**Published:** 2010-11-03

**Authors:** Li Huang, Dana Boyd, Whitney M Amyot, Andrew D Hempstead, Zhao-Qing Luo, Tamara J O'Connor, Cui Chen, Matthias Machner, Timothy Montminy, Ralph R Isberg

**Affiliations:** 1 Department of Molecular Biology and Microbiology, Tufts University School of Medicine150 Harrison Avenue, Boston, MA 02111, USA; 2 Department of Microbiology and Molecular Genetics, Harvard Medical SchoolBoston, MA 02115, USA; 3 Howard Hughes Medical Institute150 Harrison Avenue, Boston, MA 02111, USA

## Abstract

*Legionella pneumophila* promotes intracellular growth by moving bacterial proteins across membranes via the Icm/Dot system. A strategy was devised to identify large numbers of Icm/Dot translocated proteins, and the resulting pool was used to identify common motifs that operate as recognition signals. The 3′ end of the *sidC* gene, which encodes a known translocated substrate, was replaced with DNA encoding 200 codons from the 3′ end of 442 potential substrate-encoding genes. The resulting hybrid proteins were then tested in a high throughput assay, in which translocated SidC antigen was detected by indirect immunofluorescence. Among translocated substrates, regions of 6–8 residues called E Blocks were identified that were rich in glutamates. Analysis of SidM/DrrA revealed that loss of three Glu residues, arrayed in a triangle on an α-helical surface, totally eliminated translocation of a reporter protein. Based on this result, a second strategy was employed to identify Icm/Dot substrates having carboxyl terminal glutamates. From the fusion assay and the bioinformatic queries, carboxyl terminal sequences from 49 previously unidentified proteins were shown to promote translocation into target cells. These studies indicate that by analysing subsets of translocated substrates, patterns can be found that allow predictions of important motifs recognized by Icm/Dot.

## Introduction

*Legionella pneumophila* is a Gram-negative bacterium that maintains a facultative intracellular lifestyle in a wide variety of host cells (Horwitz and Silverstein, 1980; Barbaree *et al*., 1986; Henke and Seidel, 1986). Originally identified as the causative agent of Legionnaire's disease (Edelstein and Finegold, 1979), its natural hosts appear to be fresh water amoebae (Rowbotham, 1980; Henke and Seidel, 1986), which may act as reservoirs for human diseases that are acquired via aerosol of contaminated water supplies (Lasheras *et al*., 2006). After taking residence in the lung, the bacteria grow within alveolar macrophages (Davis *et al*., 1983; Jacobs *et al*., 1984), using a replication strategy that is morphologically very similar to that used in amoebae (Horwitz, 1983a; Abu Kwaik, 1996). In all cell types analysed, intracellular replication proceeds within a specialized replication compartment that recruits membrane components of the early secretory apparatus (Kagan and Roy, 2002; Derre and Isberg, 2004; Kagan *et al*., 2004) and initially avoids interaction with membrane compartments associated with the endosomal trafficking pathway (Horwitz, 1983b). Intracellular growth proceeds for 16–30 h before the host cell lyses, liberating bacteria that are primed to initiate replication in neighbouring uninfected cells. This priming involves a post-exponential phase regulatory response that ensures maximal expression of a cohort of bacterial proteins necessary for a fresh round of intracellular replication (Hammer and Swanson, 1999).

The bacterial determinant most closely associated with establishing a replication vacuole is the Icm/Dot protein translocation apparatus, a type IVb secretion system highly related to bacterial conjugative DNA transfer systems (Segal *et al*., 1998; Vogel *et al*., 1998). The complex of Icm/Dot proteins spans the bacterial envelope, allowing the transfer of proteins from the bacterial cytoplasm across membranes located in the target host eukaryotic cell (Vincent *et al*., 2006). Of the more than 20 *icm*/*dot* genes identified, most are required for intracellular growth (Segal *et al*., 1998; Vogel *et al*., 1998) and establishment of the replication vacuole, indicating that Icm/Dot-translocated substrates (IDTS) control construction of the replication compartment (Swanson and Isberg, 1995; Wiater *et al*., 1998). Several of the IDTS have been shown to directly regulate membrane traffic associated with the movement of vesicles along steps in the early secretory system (Kagan and Roy, 2002; Nagai *et al*., 2002; Machner and Isberg, 2006; Murata *et al*., 2006; Habyarimana *et al*., 2008), modulate the host ubiquitination system (Price *et al*., 2009; Ensminger and Isberg, 2010), or interfere with host protein synthesis (Belyi *et al*., 2008). While the importance of the latter two activities is unclear, control of early secretory system components by *L. pneumophila* proteins provides a potential molecular explanation for how the replication vacuole is constructed. Chief among the *L. pneumophila* proteins that control vesicle traffic are three IDTS, DrrA/SidM, LidA and LepB, that regulate the activation and GTPase cycle of Rab1, a small Ras-like protein that controls endoplasmic reticulum-derived membrane trafficking to the Golgi (Ingmundson *et al*., 2007; Machner and Isberg, 2007). Although these bacterial substrates target proteins that control host cell secretory traffic, most of the IDTS are dispensable for replication vacuole formation, indicating that there may be considerable functional redundancy in this system (Luo and Isberg, 2004).

Thus far, over 100 potential *L. pneumophila* IDTS have been identified (Luo and Isberg, 2004; de Felipe *et al*., 2005; Zusman *et al*., 2007; Altman and Segal, 2008; Burstein *et al*., 2009). Many of the identified IDTS have regions showing strong sequence similarities to domain families that are primarily eukaryotic in nature (de Felipe *et al*., 2005). Substrates have been identified either bioinformatically (Cazalet *et al*., 2004), by predicted transcriptional regulatory properties (Zusman *et al*., 2007), or by directly screening for translocation of protein fusions having assayable enzymatic activities. The original screen for translocated substrates was based on the assumption that proteins moved into host cells by the Icm/Dot system contained sequence information at the extreme carboxy terminus that can be recognized by the translocation apparatus (Luo and Isberg, 2004). This was based on the demonstration that translocation signals are located in the carboxyl terminal of proteins involved in conjugative DNA transfer by related type IV secretion systems (Vergunst *et al*., 2000). As few as 20 amino acids from the terminus of one of the IDTS is capable of conferring high efficiency translocation (Nagai *et al*., 2005). Although similar signals appear to exist in all IDTS, the substrates are not uniform in their translocation efficiencies and there is sequence information elsewhere within these peptides that modulates movement into host cells (Ninio *et al*., 2005). A large number of the substrates contain regions that antagonize translocation, causing a requirement for the IcmS/IcmW complex to act as a putative secretion chaperone that binds these regions. The dependence on this chaperone varies greatly among the substrates (Cambronne and Roy, 2007).

Two bioinformatics studies have provided evidence for amino acid preferences in the translocation signal (Kubori *et al*., 2008; Burstein *et al*., 2009). One of these studies identified candidates based on a complex series of traits expected for substrates, including regulatory and sequence dispersion data (Burstein *et al*., 2009), while the other was based on scanning sequences of known IDTS (Kubori *et al*., 2008). Both came to the conclusion that there was a preference for short polar amino acids located in the carboxyl terminal 20 residues, and that negatively charged amino acids were enriched in the region providing the putative signal. No specific consensus sequences have been identified that confer translocation among the known substrates, however, and there has been little genetic interrogation of the known targeting signals other than the demonstration that a hydrophobic amino acid must exist near the extreme carboxy terminus of the IDTS (Nagai *et al*., 2005).

In this study we have expanded the spectrum of characterized IDTS by performing a screen for translocation substrates in which a known signal from an IDTS is replaced with carboxyl terminal fragments from over 400 ORFs encoding potential translocated substrates. The screen revealed a motif containing multiple glutamates found in more than half the IDTS. Using the information from this hunt and previous studies, the glutamate-rich motif was used to identify additional translocated substrates encoded by *L. pneumophila*.

## Results

### Direct screen for identification of translocation signals

**Figure 1:**
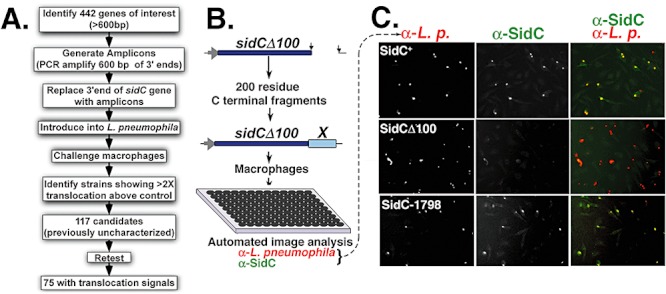
SidC fusion assay allows identification of carboxyl terminal sequences from *L. pneumophila* proteins that promote protein translocation into macrophages. A. Flowchart of procedure that allowed identification of carboxyl terminal translocation signals. B. Construction and screening of gene bank. The plasmid pZL204 (*Experimental procedures*) encodes the *sidC* gene under *Ptac* control, has a multi-cloning site and a premature truncation removing 100 codons from the 3′ end of the gene, allowing expression of a protein that is translocation defective. Rescue of the translocation defect was performed by introducing a bank of 600 nucleotide-long fragments amplified from the 3′ ends of 442 genes encoding candidate translocated substrates (*sidC-*X). The resulting gene fusion constructions were introduced into *L. pneumophila* strain Lp02*ΔsidC* and used to challenge bone marrow-derived macrophages for 1 h before fixation and staining with anti-*L. pneumophila* and anti-SidC antisera in 96-well microtiter plates. The stained samples were subjected to image analysis by automated microscopy, and the efficiency of translocation was assayed quantitatively (*Experimental procedures*). C. Examples of images captured during high throughput screening. Macrophage infections are displayed after challenge with three different strains. SidC^+^: *L. pneumophila* encoding intact *sidC* gene under *ptac* control. SidCΔ100: *L. pneumophila* harbouring pZL204 plasmid. SidC-1798: *L. pneumophila* harbouring a plasmid encoding *sidCΔ100* fused to the 3′ end sequence of *Lpg1798*. Cells were immunostained using: α-*L.p.*, mouse monoclonal antibody directed against *L. pneumophila*; α-SidC, rabbit antisera directed against the SidC protein. Rightmost panels are merged images of the two antibody probings, coloured as noted, with yellow spots denoting bacteria translocating SidC, and red indicating bacteria defective for SidC translocation.

Icm/Dot-translocated substrates have been identified using a variety of approaches, none of which used a direct screen for measuring substrate delivery into known target cells as the primary strategy. To develop a comprehensive screening strategy with as few biases as possible, an assay was developed to allow detection of proteins exported by the Icm/Dot system using scanning of microtiter wells by fluorescence microscopy (Fig. 1). The assay takes advantage of the fact that antibody directed against the IDTS SidC is highly specific, and reveals the presence of the protein about the *L. pneumophila* vacuole 1 h after uptake of bacteria into cultured bone marrow-derived macrophages (Luo and Isberg, 2004). Translocation of the protein requires an intact Icm/Dot system and sequences located in the carboxyl terminal 100 amino acids, as truncation of SidC before this region interferes with its detection about the replication vacuole (VanRheenen *et al*., 2006). The defect can be quantitated by capturing low power (20×) images of samples probed with anti-*L. pneumophila* and anti-SidC, determining the percentage of *L. pneumophila* that show colocalization with SidC (Fig. 1, αSidC: compare SidC^+^ with SidCΔ100). This strategy allows identification of translocation signals, as the introduction of sequences encoding the carboxy terminus of the previously uncharacterized protein Lpg1798 rescued the translocation defect of SidCΔ100. *L. pneumophila* encoding a SidC–Lpg1798 fusion protein had high levels of SidC detected about the *Legionella* containing vacuole (Fig. 1, SidC-1798).

Proteins that encode translocation signals were next identified by screening a bank of 442 ORFs from the *L. pneumophila* Philadelphia 1 genome for their ability to restore translocation to the SidCΔ100 fragment. The candidates were predicted to encode proteins larger than 200 amino acids (Table S1; Chien *et al*., 2004), and were chosen based on: (i) their sequence similarity to eukaryotic genes, (ii) similarity to other known bacterial virulence factors or (iii) lack of significant similarity to genes encoded by organisms other than *L. pneumophila* or the closely related *Coxiella burnetti*, which also encodes an Icm/Dot Type IVb secretion system. The candidate library was constructed by fusing 600 nucleotides from the 3′ end of each of these ORFs to the 3′ end of the *sidCΔ100* coding region, followed by introducing the fusion constructions on a plasmid into *L. pneumophila ΔsidC* (*Experimental procedures*). The resulting bacterial strains were grown in culture and used to challenge bone marrow macrophages seeded into 96-well microtiter dishes having optically clear bottoms, followed by fixation and probing by indirect immunofluorescence with anti-SidC serum and monoclonal anti-*L. pneumophila* (Fig. 1, *Experimental procedures*). The wells were then subjected to automated quantitative fluorescence microscopy analysis, with the images interrogated for the percentage of bacteria having colocalized SidC (*Experimental procedures*).

Of the 442 candidate fusions analysed in triplicate, 117 were selected for further analysis based on the criteria that they showed at least twofold higher SidC colocalization than the SidCΔ100 negative control and that there had been no published evidence for translocation of these proteins at the time the assays were initiated. The corresponding plasmids were isolated from each of the candidates, sequenced, reintroduced into *L. pneumophila ΔsidC*, and each was analysed in triplicate by automated fluorescence microscopy in repeated assays. Consistently, 75 fusions were found to confer SidC colocalization with the *L. pneumophila* vacuole (Table 1, *P* < 0.05). Of this group, 32 proteins were demonstrated to have translocation signals by other groups after this analysis was initiated (Table 1B; de Felipe *et al*., 2008; Kubori *et al*., 2008; Burstein *et al*., 2009). Most of the remaining 43 are uncharacterized, with the exception of four that are encoded by genes co-regulated with other IDTS genes that have not been previously demonstrated to be translocated [Table 1A; Ceg11, Ceg15, Ceg18, Ceg28; (Zusman *et al*., 2007)]. Genes identified in this fashion were called *rav* (*r*egion *a*llowing *v*acuole colocalization) or *mav* (*m*ore regions *a*llowing *v*acuole colocalization). Of this group of 43 genes encoding translocation signals, the predicted proteins encoded by eight showed strong predictions for coiled-coil regions, as well as one having ankyrin repeats (Table 1). These are structural characteristics observed in a number of other characterized IDTS.

**Table 1:** (A) Translocation efficiency of carboxyl terminal SidC fusions to previously unidentified proteins. (B) Translocation efficiency of carboxyl terminal SidC fusions to identified proteins that were previously shown to be translocated.

A					
Fusion	Gene name	Annotation	Translocation efficiency (%)^a^	*P*-value^b^	Reference
Lpg0008	*ravA*	Coiled-coil	66.6 + 5.7		
Lpg0030	*ravB*	Coiled-coil	69.7 ± 13.9		
Lpg0107	*ravC*	COG1723	72.2 ± 6.6		
Lpg0160	*ravD*		28.5 ± 2.1	*P* < 0.023	
Lpg0195	*ravE*		43.1 ± 6.5	*P* < 0.017	
Lpg0196	*ravF*	Lpg1752,Lpg1387 paralog	57.7 ± 3.0		
Lpg0210	*ravG*		44.2 ± 2.4	*P* < 0.0003	
Lpg0401	*ceg11*		33.4 ± 1.6	*P* < 0.004	Zusman *et al*. (2007)
Lpg0439	*ceg15*		54.4 ± 18.4		Zusman *et al*. (2007)
Lpg0733	*ravH*	Coiled-coil	48.8 ± 3.4	*P* < 0.0006	
Lpg0898	*ceg18*		83.6 ± 1.8		Zusman *et al*. (2007)
Lpg0926	*ravI*		34.4 ± 3.3	*P* < 0.015	
Lpg0944	*ravJ*		48.7 ± 5.7		
Lpg0969	*ravK*		35.6 ± 7.9	*P* < 0.08	
Lpg1108	*ravL*	Esterase	39.5 ± 7.6	*P* < 0.02	
Lpg1109	*ravM*	Coiled-coil	26.3 ± 2.7	*P*^c^ < 0.02	
Lpg1111	*ravN*		45.3 ± 2.8	*P* < 0.0007	
Lpg1129	*ravO*		80.8 ± 16.4		
Lpg1152	*ravP*		45.4 ± 2.1	*P* < 0.0004	
Lpg1154	*ravQ*		41.2 ± 1.4	*P* < 0.0006	
Lpg1166	*ravR*	Coiled-coil	46.2 ± 13.2		
Lpg1183	*ravS*		58.7 ± 13.1		
Lpg1316	*ravT*	Coiled-coil	37.9 ± 2.3	*P* < 0.002	
Lpg1317	*ravW*	Coiled-coil	42.0 ± 1.6	*P* < 0.0006	
Lpg1489	*ravX*		52.1 ± 2.9		
Lpg1551	*ravY*		65.4 ± 2.2		
Lpg1683	*ravZ*		80.1 ± 4.2		
Lpg1687	*mavA*	Ankyrin repeats	34.0 ± 1.8	*P* < 0.026	
Lpg1752	*mavB*		42.1 ± 11.9	*P* < 0.0008	
Lpg1797	*rvfA*	Paralog *ravF*, Lpg1387	60.7 ± 9.1		
Lpg2147	*mavC*	Paralog 2148	46.6 ± 3.6	*P* < 0.001	
Lpg2199	*mavD*		66.6 ± 4.2		
Lpg2311	*ceg28*		60.6 ± 10.3		Zusman *et al*. (2007)
Lpg2344	*mavE*		37.8 ± 1.8	*P* < 0.002	
Lpg2351	*mavF*		73.1 ± 6.2		
Lpg2391	*sdbC*	Paralog *sidB*,	46.3 ± 8.5	*P* < 0.006	
Lpg2424	*mavG*		58.6 ± 8.8		
Lpg2425	*mavH*	SH3-domain?	65.4 ± 7.7		
Lpg2444	*mavI*		46.8 ± 6.2		
Lpg2498	*mavJ*		28.1 ± 5.6	*P* < 0.034	
Lpg2525	*mavK*	F-Box?	50.4 ± 0.8		
Lpg2526	*mavL*		49.5 ± 1.7		
Lpg2577	*mavM*		44.2 ± 13.5		
Lpg2815	*mavN*	Rgryl_010007 similarity	42.5 ± 4.3	*P* < 0.0006	
Lpg2879	*mavO*		23.4 ± 0.3	*P* < 0.04	
Lpg2884	*mavP*		87.9 ± 2.5		
Lpg2975	*mavQ*		30.7 ± 8.1	*P* < 0.03	
*ΔsidC100*		Negative control^d^	12.7 ± 2.2		

B					
Fusion	Gene Name	Annotation	Translocation Efficiency (%)^a^	*P*-value^b^	Reference
Lpg0012	CegC1		42.6 ± 2.1	*P* < 0.00073	Altman and Segal (2008)
Lpg0059	Ceg2		26.4 ± 3.2	*P* < 0.04	Zusman *et al*. (2007)
Lpg0096	Ceg4		55.6 ± 14.1		Burstein *et al*. (2009)
Lpg0191	Ceg5		79.5 ± 2.0		Burstein *et al*. (2009)
Lpg0402	LegA9		68.1 ± 6.5		de Felipe *et al*. (2008)
Lpg0403	LegA7		53.9 ± 13.8		de Felipe *et al*. (2008)
Lpg0518			36.6 ± 2.3	*P* < 0.004	Kubori *et al*. (2008)
Lpg0634			66.8 ± 2.4		Kubori *et al*. (2008)
Lpg0696	Lem3		88.2 ± 3.0		Burstein *et al*. (2009)
Lpg0968	SidK	Coiled-coil	72.7 ± 4.8		Xu *et al*. (2010)
Lpg1120	Lem6		31.0 ± 3.0	*P* < 0.0002^c^	Burstein *et al*. (2009)
Lpg1121	Ceg19		81.2 ± 5.0		Burstein *et al*. (2009)
Lpg1145	Lem7		74.4 ± 10.1		Burstein *et al*. (2009)
Lpg1148			67.0 ± 15.1		Kubori *et al*. (2008)
Lpg1158			101.9 ± 3.1		Kubori *et al*. (2008)
Lpg1290	Lem8		36.6 ± 6.3	*P* < 0.027	Burstein *et al*. (2009)
Lpg1588	LegC6		87.0 ± 11.7		de Felipe *et al*. (2008)
Lpg1602	LegL2		59.7 ± 7.6		de Felipe *et al*. (2008)
Lpg1851	Lem14		86.9 ± 3.5		Burstein *et al*. (2009)
Lpg1949	Lem17		80.3 ± 1.5		Burstein *et al*. (2009)
Lpg1963	PieA/LirC		89.1 ± 3.8		Ninio *et al*. (2009); Zusman *et al*. (2008)
Lpg1976	LegG1		38.4 ± 6.2	*P* < 0.02	de Felipe *et al*. (2008)
Lpg1978	SetA		59.1 ± 8.4		Heidtman *et al*. (2009)
Lpg2137	LegK2		76.1 ± 2.16		de Felipe *et al*. (2008)
Lpg2248	Lem21		91.7 ± 5.4		Burstein *et al*. (2009)
Lpg2322	LegA5		36.8 ± 14.8	*P* < 0.007	de Felipe *et al*. (2008)
Lpg2327	Ceg6		91.1 ± 2.9		Kubori *et al*. (2008)
Lpg2392	LegL6		59.5 ± 4.8		de Felipe *et al*. (2008)
Lpg2504	Ceg32		31.3 ± 2.9	*P* < 0.02	Burstein *et al*. (2009)
Lpg2529	Lem27		64.3 ± 3.2		Burstein *et al*. (2009)
Lpg2603	SdmB/Lem28		48.0 ± 3.7		Burstein *et al*. (2009)
Lpg2793	LepA		54.7 ± 2.2		Chen *et al*. (2007)
*ΔsidC100*	Negative control^d^		12.7 ± 2.2		

SidC fusions to carboxyl termini of the orfs noted were introduced in *L. pneumophila* Lp02 and assayed for translocation into bone marrow derived macrophages using the immunofluorescence assay described (Experimental Procedures). Shown are data from typical experiments, except where data from several experiments were pooled, as noted.

a Displayed are the percentage of bacteria showing positive SidC staining relative to the control strain harboring a plasmid with intact SidC^+^ gene (set as 100% efficiency). Data are means ± standard error of 3–4 incubations of macrophages with *L. pneumophila* derivatives.

b Displayed are P values of two tailed T test Assuming Equal Variances, comparing negative control to noted samples. Values were determined for all samples in which fusions showed translocation efficiencies that were less than 45% of that observed for the intact SidC control plasmid, and are determined for single experiments n = 3–4 infections.

c Data are pooled from two experiments, n = 8 macrophage infections, to determine significance.

d Data are pooled from all experiments performed, n = 23 macrophage infections with Lp02(pΔ*sidC100*).

### Determinants of hybrid protein translocation

**Figure 2:**
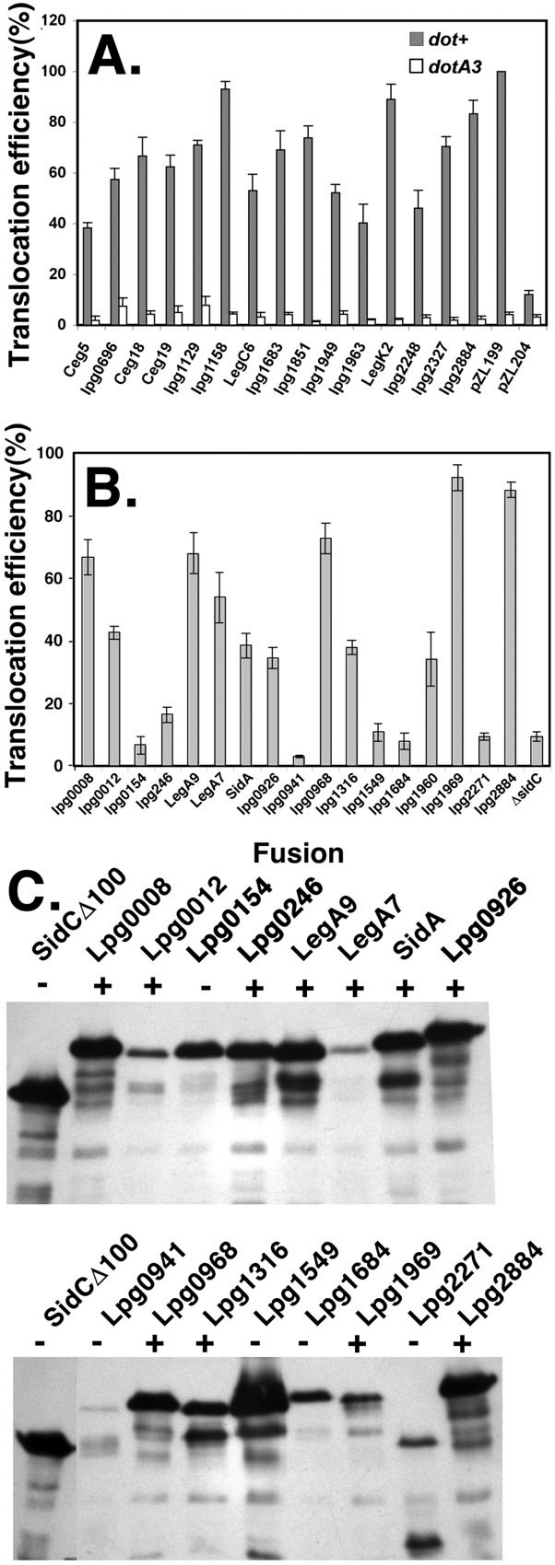
Efficient translocation of hybrid proteins requires the Icm/Dot system. A. Plasmids encoding 15 of the hybrid proteins that translocated efficiently (Table 1) were introduced into Lp02(*dot*^+^) and the *dotA^-^* strain Lp03. Translocation of each SidC fusion into bone marrow-derived macrophages was assayed in 96-well plates using standard immunofluorescence detection of SidC antigen about the *Legionella*-containing vacuole followed by image capture and quantitative analysis (*Experimental procedures*). Plasmids pZL199 and pZL204 encode full-length *sidC* and *sidCΔ100, respectively (Experimental procedures*). Data are expressed as mean ± SE of quadruplicate samples. Shown is a typical experiment. B. Translocation of hybrid proteins shows a range of efficiencies. Assay performed identically to panel A. C. SidC hybrid proteins expressed in *L. pneumophila* show a range of steady state levels. Bacteria were grown in AYE supplemented with IPTG to A_600_ = 3.2 before lysis, volumes adjusted to account for variation in density, fractionated by SDS gel electrophoresis, transferred to membranes and probed with affinity purified rabbit anti-SidC (*Experimental procedures*). +: positive for translocation of hybrid protein. −: no detectable translocation of hybrid protein based on SidC translocation assay.

To determine if vacuolar colocalization of the SidC hybrid proteins was dependent on the Icm/Dot translocation system, 15 of the plasmids encoding the SidC hybrid proteins were introduced into *L. pneumophila ΔsidCdotA3*, a mutant having a defective Icm/Dot translocation system and lacking endogenous SidC. In each case vacuolar localization of SidC antigen required the presence of the Icm/Dot system, consistent with the model that the carboxyl terminal signals present in these fusions were recognized by the translocation system (Fig. 2A).

One model for how the carboxy terminal fragments could lead to translocation of SidC is that the fragments stabilize the SidCΔ100 truncation, leading to increased steady state levels of the protein and higher frequency of SidC colocalization with the *Legionella* vacuole. To investigate this possibility, a group of hybrids that were either translocation proficient or incompetent were analysed by immunoblotting with anti-SidC. In general, there was a poor correlation between steady state levels of the hybrids and frequency of vacuoles staining with anti-SidC. For instance, steady state levels of the SidCΔ100, SidC-Lpg0154 and SidC-Lpg1549 were extremely high, but none were translocation competent (Fig. 2B and C). On the other hand, SidC-Lpg0012 and SidC-Lpg1969 were poorly expressed, but were readily observed to be associated with intracellular bacteria (Fig. 2B and C). Therefore, translocation competency is not a result of stabilization of the SidCΔ100 construction. This does not eliminate the possibility, however, that fusion proteins such as SidC-Lpg0941, which is poorly expressed and not translocated, are false negatives in this hunt.

### Identification of a common sequence found in the carboxyl termini of translocated substrates

**Figure 3:**
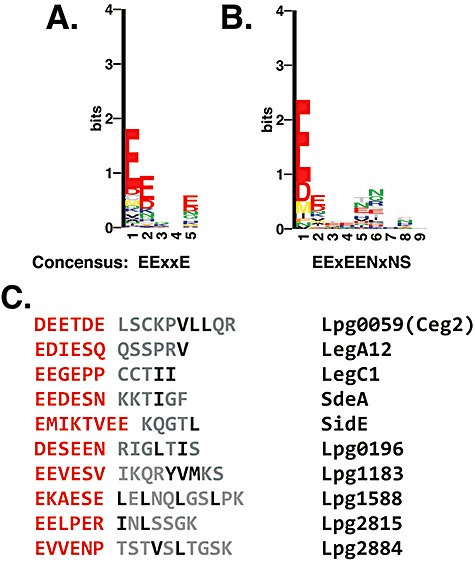
Identification of the E Block as a common element of the carboxyl termini of translocated substrates. Shown are two consensus sequences identified using BLOCK MAKER search (Henikoff *et al*., 1995) of the carboxyl terminal 30 amino acids of set of 98 proteins (Table S3) observed to be rich in glutamates. A. BLOCK identified by MOTIF program (Smith *et al*., 1990). B. BLOCK identified using GIBBS sampler program (Henikoff *et al*., 1995; Neuwald *et al*., 1995). C. Examples of previously identified translocated substrates and extreme carboxyl terminal sequences from translocation competent SidCΔ100 fusions (Table 1). In red are residues that contain a block of Glu residues, in black are hydrophobic residues that are putatively important for translocation competency (Nagai *et al*., 2005).

One hundred and eighty-two proteins, identified by pooling the data from both the SidC assay and published IDTS (Table S2), were subjected to a bioinformatic search to identify common motifs in the carboxyl terminal 75 amino acids that could distinguish translocated substrates from other *L. pneumophila* proteins (*Experimental procedures*). The BLOCKS routine (http://blocks.fhcrc.org/blockmkr/) indicated that motifs may exist in the carboxyl terminal 30 amino acids, so the search was then limited to this region of each protein (Table S2; *Experimental procedures*). When this search was repeated, glutamate-rich motifs were identified in 98 of the proteins that were searched (Fig. 3A). This represents 50% of the known translocated substrates. Depending on the algorithm used, the motifs were 5–9 residues long, with multiple glutamate residues at the amino terminal end of the motifs. At residue sites showing enrichment for Glu, there was also a bias towards Asp, indicating a preference for acidic residues in this motif. Although there exist IDTS that did not have easily identifiable examples of this Glu-rich motif, it has been previously reported that there is a bias for acidic residues within the carboxyl terminus of the IDTS, so even proteins that are not rich in Glu at the carboxyl terminus may have critical acidic residues that contribute to the translocation signal (Burstein *et al*., 2009). Examples of 10 IDTS that have identifiable Glu-rich regions in their carboxyl termini (called E Blocks throughout) are displayed (Fig. 3C).

**Figure 4:**
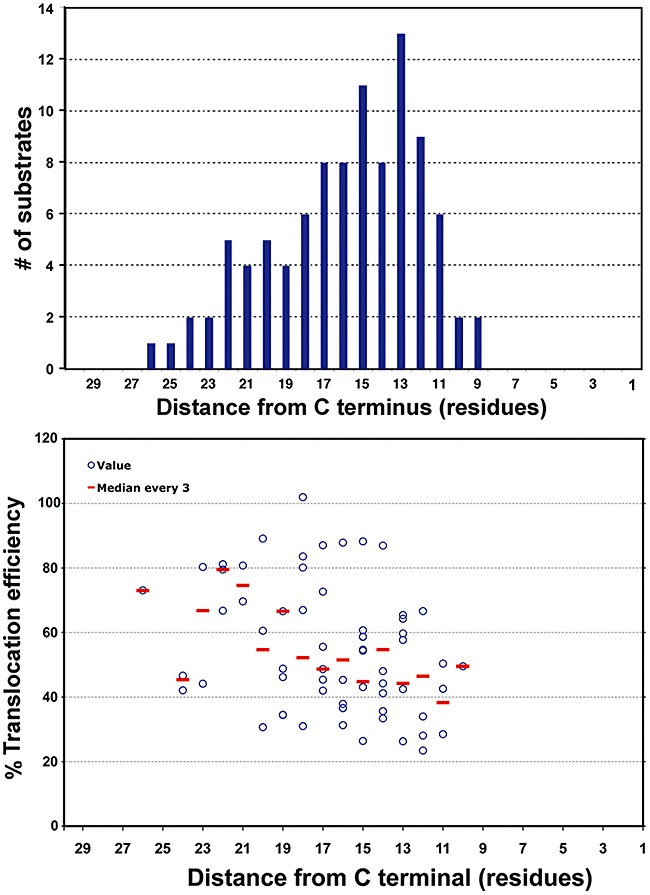
The E Block is located within 25 amino acids of the carboxyl terminus of translocated substrates (A). Distance of the E block from the carboxyl terminus of putative translocated substrates. A group of 98 substrates and putative substrates (Table S5) were inspected visually for the presence of two or more Glu or Asp residues in a string of 6–8 contiguous amino acids in the carboxyl terminal of each protein. The distance from the terminus is defined as starting from the most amino terminal residue in each block. The number of substrates having the first Glu/Asp residue at the noted site was plotted as a function of the distance of each from the carboxyl terminal. B. The translocation efficiency of 64 SidC fusions with proteins having recognizable E Blocks (listed in Table S6) was determined by the high throughput immunofluorescence assay (*Experimental procedures*). The translocation efficiency of each is plotted as a function of the distance of the beginning of the E Block from the extreme C terminus. Circles indicate the translocation efficiencies for individual fusion proteins. Red bars denote the medians for each site, ±1 residue. For example, the median at residue 15 was calculated from the translocation efficiencies of fusions having E Blocks starting at either residue 14, 15 or 16 from the C terminus.

The 98 substrates were subjected to visual inspection to identify the region of the carboxyl termini that had the E Block (Table S3). The visually identified E Blocks had 3–6 acidic residues in a region spanning 6–10 residues (Table S3), similar to the consensus length and amino acid composition determined by the BLOCK algorithm. Among this population of IDTS, the site of each E Block was not uniform, with some preference for the most amino terminal glutamate of the block being located between 11 and 18 residues from the extreme carboxyl terminus of the protein (Fig. 4A). Although proteins with the highest translocation efficiencies appeared somewhat biased towards having E Blocks more distant from the carboxy terminal end, there was a wide range of efficiencies among the fusions (Fig. 4B). It is likely that differences in protein stability and folding among the various SidC fusions (Fig. 2C) obscured any site preference for the E Block. Rather, the distribution plot of E Block sites (Fig. 4A) may be a better indicator of preferred sites.

We determined if carboxyl terminal motifs containing multiple glutamates were significantly enriched in IDTS compared with other *L. pneumophila* proteins by calculating *z* scores for the presence of these motifs relative to their expected relative abundance. To perform this analysis, the region spanning 8 to 25 amino acids from the extreme carboxyl terminus was specifically interrogated, because this region contained most of the Glu-rich blocks of sequences (Fig. 4A). We calculated expected means and standard deviations for three short motifs commonly found in this region of IDTS (ExxE, ExE and EE, Table 2) as described in *Experimental procedures*. A *z* score was calculated by determining the number of standard deviations that the actual data varied from the mean of the expected number of occurrences of the noted motif, if it occurred randomly. All three motifs showed impressive *z* scores for the group of IDTS (ranging from *z* = 10.0 to 12.1), indicating that the frequency of each was much higher than expected. For instance, among the 182 IDTS analysed, the ExxE motif was found 53 times, compared with the expected 12 ± 3 occurrences (Table 2, *z* = 12.1). In contrast *z* scores for the presence of these motifs in the rest of the genome were much lower (ranging from *z* = −1.7 to 3.6). The reason for this result is not because the IDTS have a larger number of these motifs throughout the length of their sequences. If the carboxyl terminal 25 amino acids are removed from the IDTS, then the frequency/residue of these motifs is 0.004. This low frequency is almost identical to what is found in the rest of the proteins encoded by *L. pneumophila*, missing the carboxyl terminal 25 amino acids (frequency/residue = 0.005). Therefore, enrichment for these motifs is only found in the carboxyl termini of the IDTS. It should be noted that the EE and ExxE motifs occurred within this 18 amino acid span in the rest of the genome somewhat more frequently than predicted by base composition (*z* = 2.4 and 3.6, respectively). This is consistent with the model that several IDTS still remain to be identified.

**Table 2:** Enrichment of double E motifs in translocated substrates.

Motif	Genome-(substrates)^a^				Translocated substrate list^b^						
	Expected^c^	Found^d^	*z* score^e^	Expected^c^	Found^d^	*z* score^e^
EE	190 ± 13	222	2.4	12 ± 3	53	11.8
ExE	180 ± 13	158	−1.7	12 ± 3	45	10.0
ExxE	170 ± 13	216	3.6	11 ± 3	51	12.1

a Genome–(substrates): all the predicted proteins in *L. pneumophila* genome, missing the 182 proteins used for BLOCKS search (Table S2).

b Translocated substrate list: 182 proteins used for BLOCKS search (Table S2).

c Expected: the amino acid composition of the −25 to −8 carboxyl terminal region for all predicted proteins in the genome was determined (*Experimental procedures*) and the expected frequency of indicated motifs based on random chance was calculated, rounded to nearest integer for ease of display.

d Found: the number of predicted proteins with noted motif that were identified in each group of predicted proteins.

e Number of standard deviations from the mean of the Expected.

z scores were determined to evaluate the significance of the noted motifs in regions located between amino acids −25 and −7 from the carboxyl terminus in target proteins, where x = any amino acid.

### The presence of a glutamate-rich region in the carboxyl terminal of SidM/DrrA is required for efficient translocation

**Figure 5:**
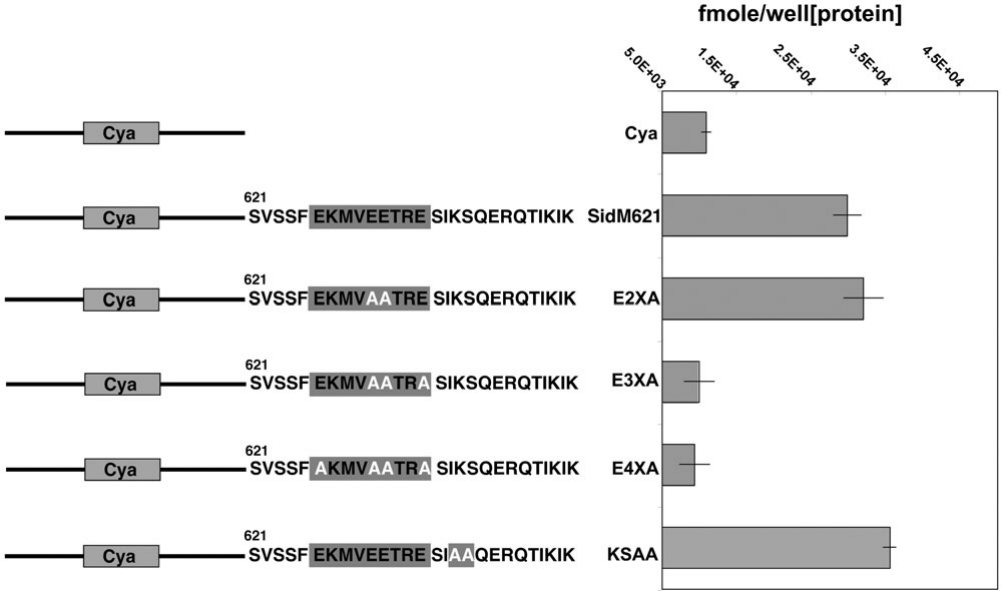
A glutamate-rich region is required for efficient translocation of cyclase fusions containing the C terminus of SidM. DNA fragments encoding the carboxyl terminal 27 amino acid region of SidM or noted mutants were fused to the *cyaA* gene and the translocation efficiency of each was determined after challenge of U937 cells with *L. pneumophila* expressing the indicated fusion (*Experimental procedures*). Displayed are the sequences fused to CyaA and the noted mutations in white lettering. Data are from one of four individual experiments, and are the mean of three replicates ± SE. To calculate activities, the amount cAMP generated per sample was normalized to the steady state levels of each fusion protein based on Western blots probing with anti-CyaA (*Experimental procedures*), to compensate for any differences in protein expression in the samples tested (fmole/well[protein]).

To determine if the presence of a series of glutamates near the carboxyl terminus modulates the efficiency of translocation via the Icm/Dot system, a previously characterized translocated substrate was analysed. SidM/DrrA, a guanine nucleotide exchange factor for Rab1 (Machner and Isberg, 2006; Murata *et al*., 2006), has a 9 amino acid region near the carboxyl terminus containing four Glu residues (Fig. 5, gray boxes). The region encoding the extreme carboxyl terminal 27 amino acid region of SidM/DrrA was fused to the 3′ end of *cyaA*, which encodes a calmodulin-dependent adenelyl cyclase (Sory and Cornelis, 1994). Fusions to *cyaA* have little cyclase activity unless the resulting hybrid is introduced into eukaryotic cells that express calmodulin. Therefore, sequence-dependent translocation of the hybrid protein into calmodulin-containing cells can be assayed by simply measuring cyclase activity after allowing contact of bacteria with host cells. This assay has been used numerous times to demonstrate translocation of IDTS (Chen *et al*., 2007; Burstein *et al*., 2009).

Differentiated U937 cells (*Experimental procedures*) were challenged for 1 h with *L. pneumophila* strains harbouring *cyaA* fusions to the carboxyl terminal 27 codons of the *sidM* gene, and the efficiency of cAMP formation was determined, normalizing to the amount of fusion protein expressed by each strain (*Experimental procedures*; fmoles/[CyaA fusion]). A CyaA fusion having the wild-type carboxyl terminus of SidM showed a clear increase in cyclase activity relative to a strain encoding the *cyaA* ORF (Fig. 5; compare Cya with SidM621). Similar high levels of cyclase activity were observed when two of the four Glu residues in the putative E Block region were changed to Ala, indicating that not all Glu residues in this block are required for the formation of a translocation signal (Fig. 5, E2XA; E630A/E631A). On the other hand, when either three (E630A/E631A/E634A), or four (E626/E630A/E631A/E634A) of the Glu residues were changed to Ala, there was no detectable increase in cyclase activity relative to the negative control protein, indicating that some combination of these three residues are required for efficient translocation (Fig. 5, compare E4XA and E3XA with Cya). As an additional control, a double Ala mutant was constructed replacing residues outside the E block, which showed no decrease in cyclase activity relative to the wild-type fusion, consistent with a wide range of side chains being allowed in this region (Fig. 5, compare KSAA with SidM621; K637A/S638A). These data were all normalized to the relative steady state levels of protein, based on immunoblotting, so defective translocation was not due to decreased levels of the hybrids, which showed steady state levels close to that of the wild-type CyaA–SidM fusion (*Experimental procedures*).

### Identification of additional Icm/Dot translocated substrates based on the presence of a carboxyl terminal E block

The *z* score determination for the presence of E Blocks is consistent with there being IDTS that have not yet been identified, as a scan of genomic ORFs depleted of the known translocated substrates reveals more Glu-rich motifs than expected by random chance [Table 2, Genome-(Substrates)]. The existence of a number of IDTS that have carboxyl termini enriched in glutamate residues indicates that sequence similarity searches could be used to identify additional substrates. The consensus blocks identified the sequences EExxE, ExE or EEx as being found in IDTS (Fig. 3A and B), and the lack of translocation of SidM when Glu residues in this motif were mutated (EETRE) is consistent with it being part of a translocation signal (Fig. 5). Therefore, to identify more IDTS, eight blast searches were performed against the *L. pneumophila* Philadelphia 1 genome using sequence queries containing E-rich motifs, setting parameters appropriate for short sequences (*Experimental procedures*). Candidates that showed sequences similar to these motifs that were located more than 30 residues from the putative carboxyl terminus were eliminated from the analysis, resulting in 56 IDTS candidates that survived the search criteria (Table S4). Of these, 21 had been documented in the literature as IDTS in previous studies (Zusman *et al*., 2007; Altman and Segal, 2008; Kubori *et al*., 2008; Burstein *et al*., 2009), and 10 additional candidates were shown to have IDTS signals based on the SidC translocation assay described here (Table 1). Seven appeared unlikely to be translocated, based on sequence similarities to conserved hypothetical proteins found in non-pathogens (Table S4, asterisks). After accounting for the above proteins, there were 18 candidates from the blast search that were not present in the original group of IDTS used to identify the E block motif or which had not been previously identified as IDTS (Table 3).

**Table 3:** Candidate translocated substrates based on E Block search.

ORF	Gene	Features	Reference
Lpg0209	*mavR*	Similar to ceg6 (Lpg0208); 9e^−137^	Zusman *et al*. (2007)
Lpg0563		Similar to 2678 (1e^−13^)	This study
Lpg0645	*mavS*		This study
Lpg0717			This study
Lpg0921	*mavT*		This study
Lpg1663			This study
Lpg1798	*mavU*	RhoGAP domain (2e^−3^)	This study
Lpg2073			This study
Lpg2160		Lpg2638 paralog (1e-145)	This study
Lpg2395			This study
Lpg2420		GNAT protein (CD04301;6e-05)	This study
Lpg2455		Next to legA15	de Felipe *et al*. (2005)
Lpg2638	*mavV*	Lpg2160 paralog (1e-145)	This study
Lpg2552			This study
Lpg2678		Methyltransferase(CD02440; 1e-11)	This study
Lpg2806		Coiled-coil	This study
Lpg2874			This study
Lpg2907	*mavW*	Ubiquitin protease	Catic *et al*. (2007)

A blast search of the *L. pneumophila* Philadelphia 1 genome was performed, using queries of nine different Glu-rich motifs (*Experimental procedures*). Displayed are ORFs encoding proteins that have carboxyl terminal sequences similar to Glu-rich block observed in proteins translocated by the *L. pneumophila* Icm/Dot system. Removed from the list are ORFs that are members of the original pool of 182 ORFs that were used to identify the Glu-rich block as well as previously identified IDTS. Complete list of proteins is in Table S4.

These 18 candidate IDTS included a number of proteins that have sequence similarities to proteins found in eukaryotes or have motifs found primarily in eukaryotic cells [Table 3; RhoGAP domain, histone acetylase, a previously described ubiquitin protease (Catic *et al*., 2007), or coiled-coil regions]. The group also included paralogs within the *L. pneumophila* genome that showed no similarity to proteins outside the microorganism, a common property of IDTS (Cazalet *et al*., 2004; Luo and Isberg, 2004).

**Figure 6:**
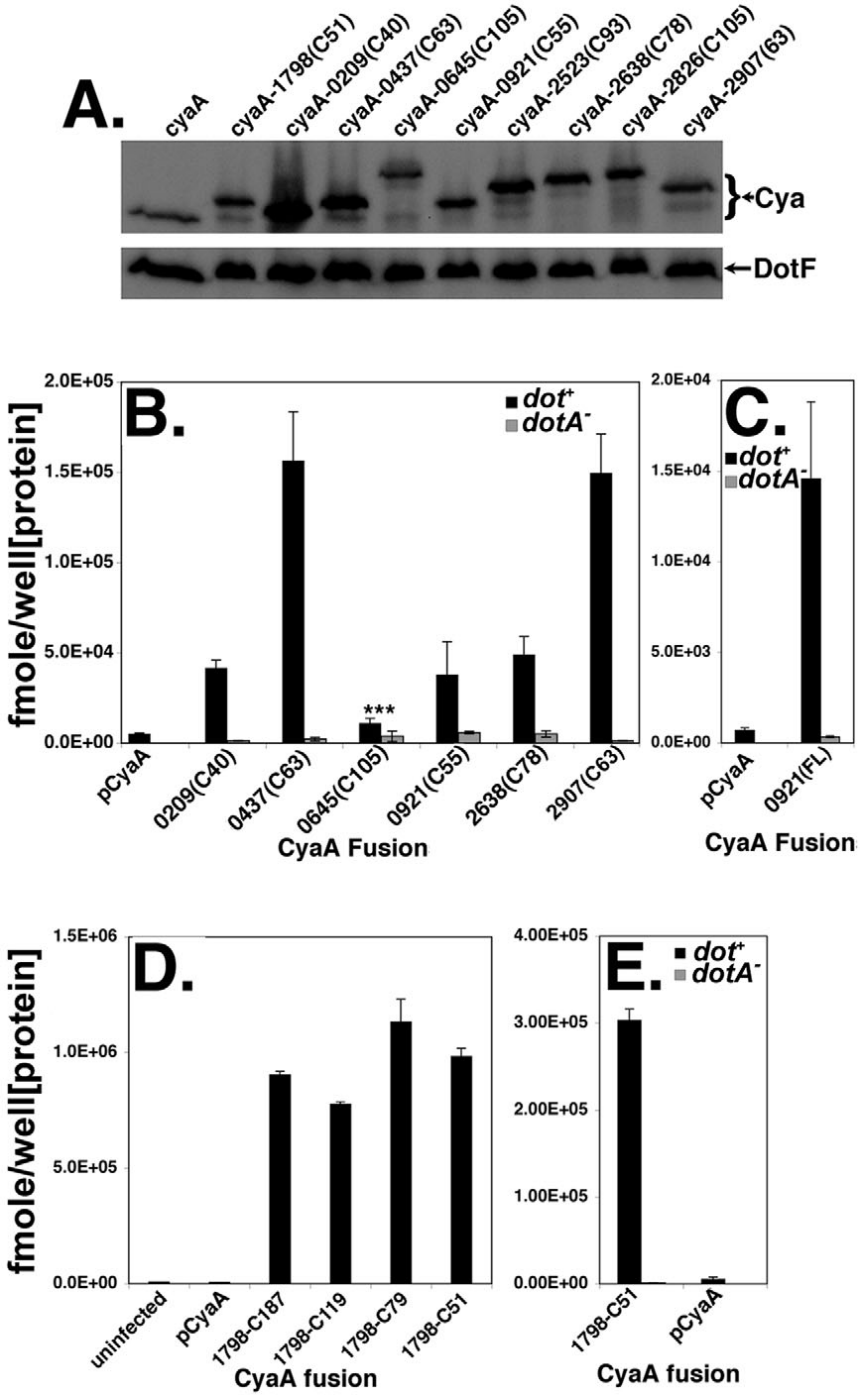
Identification of translocated substrates based on presence of E block. Gene fusions were constructed between the 3′ end of *cyaA* and the coding regions for the carboxyl termini of the nine indicated proteins, which were predicted to be translocated based on the presence of an E block. A. Western blot analysis of whole cell extracts of *L. pneumophila* strains expressing the indicated fusions, probed with anti-CyaA antibody. Numbers in parentheses indicate the length of the C terminus of each protein that was fused to cyclase. Lpg2638 shows aberrant behaviour on an SDS gel and migrates at a larger molecular weight than predicted. Anti-DotF is used as a loading control. B. Carboxyl termini having predicted E blocks promote translocation of CyaA. Cyclase fusions displayed in panel (A) were expressed in either a wild-type or *dotA^-^* strains and translocation in U937 cells was determined by assaying for calmodulin-dependent cyclase activity. C. Complete coding region of Lpg0921 (MavT) allows enhanced translocation based on cyclase assay. Translocation was determined as in panel (B) using full-length (FL) Lpg0921 fused to CyaA. D. A 51 amino acid carboxyl terminal fragment from Lpg1798 is sufficient to promote translocation of CyaA. U937 cells were challenged for 1 h with cyclase fusions to varying sized carboxyl terminal fragments of Lpg1798, and extracts were assayed for translocation by determining amount cAMP generated (*Experimental procedures*). Residues from carboxyl terminal tags are noted for each fusion, with numbers referring to the total number of residues present in each fusion. E. Translocation of CyaA-Lpg1798 fusion is Icm/Dot-dependent. Fusion having 51 amino acid carboxyl terminus was introduced into *dotA^-^* strain and translocation efficiency was assayed as in panel B. For each cyclase assay, data are from one of three independent experiments, and are the mean of three determinations ± SE. To calculate activities, the amount cAMP from each well was normalized to the steady state levels of each fusion protein, based on Western blot (fmole/well[protein]), to compensate for any differences in protein expression in the samples tested.

Nine of the IDTS candidates identified by the blast search for E blocks were analysed for the presence of carboxyl terminal translocation signals, constructing fusions to the 3′ end of the *cyaA* gene (Fig. 6). As previous fusions to SidC showed a range of steady state levels of the proteins (Fig. 2C), CyaA fusions were constructed at internal sites in the reading frames that were predicted to encode either turns or unstructured regions upstream of regions that had strong secondary structure predictions, based on the Robson-Garnier algorithm (Fig. 6A). The rationale was that CyaA fusions to intact and relatively compact domains would likely generate proteins that have high steady state levels of protein. This strategy worked, as *L. pneumophila* expressing these fusions had similar steady state levels of protein, based on immunoblotting with anti-CyaA (Fig. 6A; parentheses indicating the size of carboxyl terminal region added to CyaA). The one exception was a particularly small fusion that appeared to have much higher steady state levels than the other fusions [Fig. 6A; CyaA-Lpg0209(C40)]. Wild type or *dotA^-^ L. pneumophila* strains harbouring each of these fusions were then used to challenge U937 cells to determine if they contained translocation signals based on increases in cyclase activity, with cAMP accumulation normalized to the amounts of steady state levels of hybrid proteins found in each of the strains (Fig. 6B and C; *Experimental procedures*). Confirming previous results using a different reporter system (Burstein *et al*., 2009), incubation with bacteria harbouring hybrids composed of the carboxyl termini of Lpg2523 (Lem26) or Lpg2826 (Ceg34) yielded high levels of cyclase activity, dependent on an intact Icm/Dot System (data not shown). In addition, fusions to six of the seven others all showed at least a 10-fold increase in cyclase activity that was eliminated in a *dotA^-^* strain (Fig. 6B and E). The one exception was a particularly poorly translocated fusion to Lpg0645, although the observed increase in cyclase activity was statistically significant (Fig. 6B). Together with previous results, these data are consistent with at least 38 of the 56 candidates identified by the E Block blast having Icm/Dot translocation signals at their carboxyl termini.

All the fusions contained small regions of the carboxyl termini, and in some cases the apparent translocation efficiency was relatively low based on normalized cyclase activities. To address whether including other regions of the protein could enhance the translocation efficiency, the length of the fusion of Lpg0921 was extended to include its entire reading frame (Fig. 6C; predicted to encode 414 amino acids). The original fusion, containing the carboxyl terminal 55 residues of Lpg0921, only showed approximately sixfold higher cyclase activity than the unfused control [Fig. 6B; 0921(C55) vs. pCyaA]. In contrast, the full-length fusion showed 21-fold higher levels of cyclase activity relative to the unfused control (Fig. 6C), and was 40-fold higher when compared with a strain harbouring the full-length fusion but lacking an intact Icm/Dot system [Fig. 6C, 0921(FL), *dotA*]. Therefore, although the carboxyl terminal fragments identified by the E block search appeared to encode translocation signals, there may be other regions of these proteins that present translocation signals or which positively contribute to substrate recognition by the Icm/Dot system.

To determine if extending the length of fusions resulted in enhanced cyclase activity was a general rule of the proteins identified by the E Block search, translocation of Lpg1798 was analysed. Fusions were constructed along the length of the protein, ending at the junction between an N-terminal domain predicted to be a RhoGAP domain and the C-terminal region. Incubation of bacteria with cultured cells resulted in high levels of normalized cyclase activity for each of the fusions (Fig. 6D), dependent on the presence of an intact Icm/Dot system (Fig. 6E). In fact, the cyclase activity observed for strains encoding a 51 amino carboxyl terminal fragment of Lpg1798 was as high as any activity assayed for a large number of fusions constructed by our laboratory (data not shown), consistent with this carboxyl terminal region providing all the signals necessary for efficient translocation by the Icm/Dot system. Taken together, these data indicate that searching carboxyl terminal sequences for regions with high glutamate density is a good indicator of the presence of a translocation signal.

## Discussion

Using a targeted approach in which Icm/Dot translocation signals were identified by assaying for the presence of an exported antigen, we were able to enlarge the pool of known *L. pneumophila* translocated substrates by 49 additional proteins. This strategy had the advantage that it did not require that the proteins have predicted functions in eukaryotic cells or that they be expressed under any particular regulatory elements. The large number of proteins that encode translocation signals facilitated our ability to identify motifs found in the C-terminus of a subset of IDTS. This in turn, allowed the identification of more proteins that have translocation signals based on the presence of carboxyl termini that are rich in glutamate residues. The fact that a large number of uncharacterized proteins having translocation signals were identified indicates that this targeted approach was a highly effective strategy. Despite the overlap between many of the IDTS identified by this strategy and previously characterized substrates, the approach resulted in a significant expansion of the number of known Icm/Dot targets. As the strategy described here involved measuring translocation of a reporter into a known host cell of *L. pneumophila* rather than using transcriptional regulatory properties or yeast cell killing, it had the advantage that it directly measured the desired translocation activity. The primary disadvantage of this approach was that some fusions to SidC were either unstable or potentially resulted in poor presentation of carboxyl terminal sequences to the Icm/Dot system, preventing detection of translocation signals in these cases.

**Figure 7:**
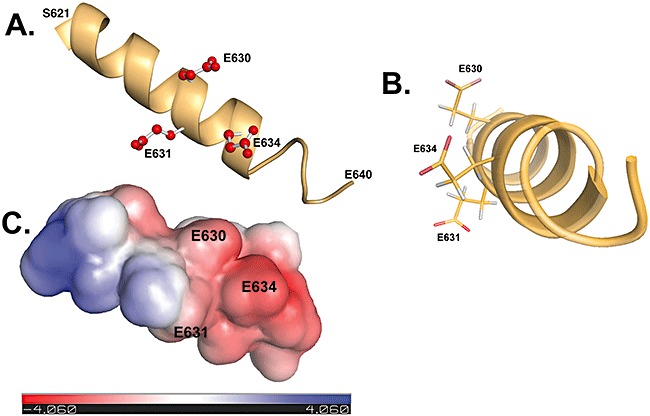
The presence of a glutamate triangle in the SidM carboxyl terminal translocation signal. A. The presence of a glutamate triangle on the α-helical surface of SidM(S621-E640). Shown is a ball and stick display of the three Glu residues across the face of the helix that were mutated in the translocation-defective CyaA-SidM derivative (Fig. 5). No structural information exists for the remaining SidM(Q641-K647) carboxyl terminal residues, which are presumably disordered (Zhu *et al*., 2010). B. The glutamate triangle is arrayed on a single face of the SidM carboxyl terminal α-helix. C. Surface potential of SidM(S621-E640) shows increased negative charge at the E634 tip of glutamate triangle. Blue: high positive charge density. Red: high negative charge density. Scale: amount of electrostatic potential in kT/e, where k is Boltzmann's constant, T is temperature °K (310°K), and e is charge of electron. Surface potential calculations were determined using APBS electrostatics plug-in on PyMol (DeLano Scientific, LLC). Structure deposited as MMDB: 78999; PDB: 3L0M, chain A (Zhu *et al*., 2010).

By performing searches for motifs within the carboxyl termini of proteins that have putative translocation signals, a subset of proteins was identified that had short stretches of residues that were rich in glutamates, called E Blocks. To determine if Glu residues contribute to recognition by the Icm/Dot system, we analysed a 27 amino acid carboxyl terminal fragment of SidM that allowed translocation of a CyaA reporter. According to one of the recently described crystal structures of SidM, this fragment largely forms an α-helix [Fig. 7A; (Zhu *et al*., 2010)]. We found that a triple Glu->Ala mutant in the helix (E630/E631/E634), located in a region hypothesized to be an E Block, eliminated translocation of the CyaA reporter (Fig. 5). These three Glu residues form a triangle of acidic side chains that line up on a hydrophilic face spanning one helical turn, in an EExxE arrangement of residues in the primary sequence (Figs 5, 7A and B). This may be a common translocation motif, as bioinformatic inspection reveals that 73 known IDTS have an (E/D)xx(E/D) sequence near the carboxyl terminus, in which the acidic residues are usually glutamates (Table S7). This sequence could allow a pair of acidic residues to form a recognition surface across one helical turn, similar to what is observed in SidM (Fig. 7A and B). Interestingly, the arrangement of Glu residues results in a gradient of negative charge density that increases across one face of the helix, peaking at the carboxyl terminal tip of the glutamate triangle (Fig. 7C, E634, high density of red colour), which may explain why profound defects in translocation were only observed in mutants including the E634A alteration. Ascribing a primary role for this surface potential gradient comes with the caveat that the region encoding the final seven residues of SidM is not included in any published models, and this presumably disordered region has three basic residues that could significantly alter the predicted electrostatic potential over the Glu triangle (Schoebel *et al*., 2009; Suh *et al*., 2010; Zhu *et al*., 2010).

As only about 50% of the known IDTS appear to have easily recognizable E Blocks, this raises the issue of whether there are two classes of sequences, with one class having a glutamate-independent signal. Multiple acidic residues, however, are present in carboxyl terminal sequences of many of the IDTS that seem to lack a clear E Block, arguing against a second class. To try to determine if we could find some other type of signal that might include acidic residue that was difficult to detect by doing primary sequence searches, we tried another approach based on the known secondary structure of the carboxyl terminal of SidM/DrrA. The carboxyl termini of a few of the IDTS that do not appear to have E Blocks were subjected to secondary structure analysis using Jpred3 (http://www.compbio.dundee.ac.uk/www-jpred/) to identify predicted α-helical regions similar to that displayed in Fig. 7. Glu residues could then be identified in helices that might arguably provide a similar face to that observed in SidM/DrrA (Fig. 7B). For instance, the carboxyl terminal of the IDTS SidC is predicted to have a short helical region encompassing the residues KQFREAMGEIT. This sequence was not identified by BLOCK searching routines, but the EAMGE peptide may place the two glutamates on a face of a helix that is similar to that seen in SidM/DrrA. Therefore, more complex searching routines may identify similarities not observed by relying solely on primary sequence analysis. The bias towards having at least one negatively charged residue in the carboxyl terminus has been previously pointed out (Burstein *et al*., 2009), so we think it likely that this charge preference is a general rule for IDTS, either stabilizing a helix or providing a recognition face for the Icm/Dot system.

Considerable attention has been devoted to the presence of a translocation signal at the carboxyl terminal end of IDTS, and the work described here has relied on the ability of carboxyl terminal fragments to promote detectable translocation of protein domains (Luo and Isberg, 2004; Nagai *et al*., 2005). These results, however, do not address whether other regions of translocated substrates contribute to recognition and movement across the Icm/Dot system. In fact, the ability of a carboxyl terminus to recapitulate the behaviour of a full-length protein appears to vary among the substrates. In the case of the IDTS Lpg1798, a 50 amino acid region was sufficient to promote translocation of CyaA, and successively larger regions of the protein do not increase the efficiency of movement (Fig. 6D). The carboxyl terminus of SidG similarly promotes very high translocation levels (Cambronne and Roy, 2007). With other IDTS, it can be seen from several examples that more than just the carboxyl terminus modulates the translocation efficiency. First, a deletion of the carboxyl terminal of SidC is not sufficient to cause total elimination of Icm/Dot-dependent translocation (Table 1). Vacuoles positively staining with anti-SidC can be readily observed in a fraction of cells after challenge of macrophages with strains harbouring the SidCΔ100 construction, indicating there exist translocation signals upstream from the carboxyl terminal that are sufficient for low-level translocation. Second, CyaA fusions to different lengths of the carboxy termini of either Lpg0921 (MavT) or SidM show translocation efficiencies that vary markedly (Fig. 6C; data not shown). Therefore, upstream regions may function to support the carboxyl terminal signal, have signals capable of promoting translocation independently of other sequences or make the translocation signal more accessible to the Icm/Dot system. Finally, there are regions of many IDTS that appear to antagonize translocation, and binding of these regions to the translocation chaperone complex IcmS/IcmW reverses this effect, stimulating translocation (Cambronne and Roy, 2007). Understanding how these various signals are coordinated by the Icm/Dot system will be key to uncovering the elements involved in molecular recognition during the translocation process.

Given that at least 193 different proteins from the *L. pneumophila* Philadelphia 1 isolates have translocation signals, this raises the question as to whether there are any more to be discovered. Based on the results from the SidC assay, approximately 50% of the known IDTS that are larger than 200 amino acids in length failed to give a clear positive translocation signal, either because the fusion proteins were unstable or they assumed translocation-incompetent conformations that interfered with the assay. We identified translocation signals in 49 previously uncharacterized proteins, so we assume that there must be approximately 50 IDTS of this size that have escaped identification. In addition, the simplifying strategy of eliminating genes that were shorter than 600 nucleotides resulted in the inability to identify some of the substrates. As about 10% of the proteins known to have Icm/Dot translocation signals are shorter than 200 amino acids, there may be more than 10 proteins that we missed because of the nature of the bank used. Therefore, we think it possible that there are more than 250 IDTS encoded by *L. pneumophila*. Nearly 10% of the genome encodes either substrates or components of the Icm/Dot system.

**Figure 8:**
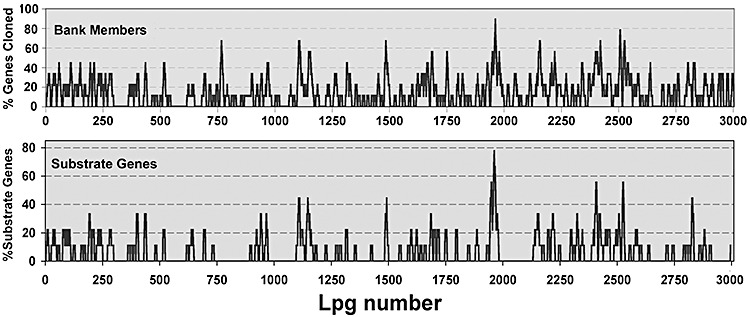
Distribution of known translocated substrates along the *L. pneumophila* Philadelphia 1 chromosome. Shown are linear representations of the single circular *L. pneumophila* genome, with 3005 annotated ORFs displayed along the chromosome on the *x* axis. Top panel shows the moving average of genes screened in the SidC translocation assay to identify IDTS. Density of genes is calculated as % of ORFs chosen for cloning in a nine gene moving window. The bottom panel displays the location on the chromosome of genes that have been identified as having Icm/Dot-dependent translocation signals in all studies to date. % substrate genes: percentage of genes in a window of nine genes that have sequences that encode Icm/Dot-dependent translocation signals.

The *L. pneumophila* chromosome does not appear to have distinct pathogenicity islands observed in other pathogens (Cazalet *et al*., 2004; Chien *et al*., 2004; Lavigne and Blanc-Potard, 2008). Even so, it was previously shown that there is a non-uniform distribution throughout the chromosome of translocated substrate-encoding genes [(Burstein *et al*., 2009); Fig. 8]. This appears to reflect the fact that there are chromosomal regions of increased plasticity with highly divergent gene compositions among different clinical isolates. The presence or absence of translocated substrate-encoding genes provides an important source of diversity among *Legionella* isolates (Cazalet *et al*., 2008; Zusman *et al*., 2008; Ninio *et al*., 2009). A similar non-uniform distribution of genes can be seen when the location of the 442 genes that were used for the fusion bank is plotted along the length of the chromosome (Fig. 8). The criteria for selection of bank members probably dictated this distribution, as we required that the genes encode proteins having no clear sequence similarities to other bacterial proteins. In fact, there was a general concordance between the representation of different regions of the genome that were analysed and regions that showed a high density of IDTS genes (Fig. 8). Therefore, identifying coding regions of low sequence similarity to bacterial proteins potentially uncovers gene clusters encoding translocated substrates.

In summary, a large number of genes harboured primarily by species related to the *Legionellaceae* encode proteins with translocation signals. Although it is likely that the SidC-based translocation assay did not exhaustively identify every substrate, it allowed a large increase in an already large pool of identified substrates, and facilitated the identification of a common motif found in a number of these proteins. Future work directed towards determining how these sequence determinants interface with the Icm/Dot system should allow investigation of the dynamics of the translocation process.

## Experimental procedures

### Cell culture and media

*Legionella pneumophila* strains were grown on buffered charcoal-yeast extract solid medium or ACES-buffered yeast extract (AYE) broth culture media (Feeley *et al*., 1979; Gabay and Horwitz, 1985; Berger and Isberg, 1993). The growth media were supplemented with thymidine at 100 εg ml^−1^ when appropriate. For *L. pneumophila*, kanamycin was used at a concentration of 20 εg ml^−1^. Antibiotics were used at the following concentrations with *Escherichia coli* strains: kanamycin, 40 μg ml^−1^; ampicillin 100 μg ml^−1^ and chloramphenicol, 30 μg ml^−1^. For challenge of host cells, *Legionella* was patched from a single colony onto buffered charcoal-yeast extract. After 2 days at 37°C, patches were used to inoculate AYE broth culture. Before challenge of mammalian cells, bacteria were grown overnight in AYE broth with appropriate additives and grown to post-exponential phase (A_600_ > 3.5) until greater than 50% of the bacteria were judged to be motile by microscopy.

Primary bone marrow-derived macrophages were isolated from the femurs of female A/J mice and maintained as described in L cell-conditioned medium (Swanson and Isberg, 1995). For assays in 96-well microtiter plates, the macrophages were removed after 1 week of bone marrow cell culture, diluted to a density of 5 × 10^5^ ml^−1^ in RPMI1640 + 10% fetal calf serum, and 100 μl of cells was introduced onto 96-well microtiter plates having optically clear bottoms (Costar, cat #3603). For adenylate cyclase assays, U937 cells were differentiated using 10 ng ml^−1^ 12-tetradecanoyl phorbol 13-acetate (TPA) for 48 h, after which cells were washed, replated in 24-well dishes with fresh media in the absence of TPA before bacterial challenge.

### Bacterial strains and plasmids

The *L. pneumophila* strains used are derivatives of the strain Lp02 [*thyA Δ(hsdR-lvh) rpsL*] (Berger and Isberg, 1993). The *L. pneumophila* derivative Lp02(*ΔsidC*) strain contains an in-frame deletion of *sidC* removing all but the regions encoding the amino and carboxyl terminal 15 codons of the gene (Luo and Isberg, 2004), while Lp02*ΔsidM* (referred to as *ΔsidM*) has a similar deletion of *sidM* (Machner and Isberg, 2007). The translocation deficient strain Lp03 contains the *dotA3* mutation (Berger and Isberg, 1993).

The plasmid pZL204 was constructed by inserting the open reading frame of the *sidCΔ100* sequence into SacI-BamHI site of pJB908 in which the region encoding the carboxyl terminal 100 amino acids of SidC was deleted and replaced with a premature stop codon. To generate reporter fusions to proteins predicted to contain carboxyl terminal translocation signals, fragments of genes were chosen that were predicted to have low levels of hydrophobicity, based on the Kyte-Doolittle algorithm, and then fused to the *cyaA* gene (Kyte and Doolittle, 1982; Sory and Cornelis, 1994). The exact endpoint of each fusion was chosen at a region predicted to have a turn or was located in between regions having predictions of strong helical structure. Fusions to the gene encoding adenyl cyclase were constructed at the 3′ end of the *cyaA* gene in the plasmid pJB2581 (kind gift from Dr J. Vogel, Washington University School of Medicine), using fragment sizes described in the text. PCR-amplified fragments were inserted in-frame in the BamHI and SalI sites of the plasmid to construct fusions.

Point mutations in the 3′ end of the *sidM* gene were constructed using QuikChange® Site-Directed Mutagenesis Kit (Stratagene Cat#200518) in plasmid pLH100, which contains the region encoding the carboxyl terminal 27 amino acids of SidM fused to the extreme 3′ end of the *cyaA* gene. Each mutant resulted in changing multiple residues for alanines, and the sites of mutations are described (Fig. 5).

### Construction of SidC fusion library

The annotation of the *L. pneumophila* Philadelphia 1 genome sequence (GenBank AE017354) was scanned for ORFs predicted to encode proteins that showed either sequence similarity to eukaryotic proteins or which showed no significant similarity to proteins encoded by organisms other than *Legionella* or *Coxiella* species. All ORFs predicted to encode proteins larger than 200 amino acids were then used for design of the fusion gene bank, amplifying regions encoding approximately 200 codons from the 3′ end of each gene. To determine the sites used for amplification by PCR, the 442 ORFs that survived filtering in this fashion were then scanned using a local copy of Primer3 (Rozen and Skaletsky, 2000) for pairs of oligonucleotides 18–25 bases in length that were located downstream of the stop codon and approximately 600 base pairs upstream from the stop codon that had similar base compositions. For each ORF, 25 pairs of oligonucleotides were chosen as potential oligonucleotide pairs for amplification of the 3′ end of each gene, ranking each pair based on melting temperature similarities. The highest ranking oligonucleotide pairs were then used to generate an *in silico* PCR product for a blast search against the *L. pneumophila* Philadelphia 1 genome to determine if there were any significant sequence similarities that could lead to inappropriate sites of priming. The highest ranking oligonucleotides having low probabilities of aberrant priming were retained for the construction of the bank. Unless otherwise noted (Table S1), the restriction sites BamHI and XbaI were added to the 5′ and 3′ amplification primers, respectively, to allow insertion of PCR products into the plasmid. If either BamHI or XbaI was found to be located in the region amplified, then alternate restriction sites were used. Table S1 lists the ORFs amplified, the sequences of the primers used, the restrictions sites added to each end and the annotations for each of the genes amplified.

Using the primers identified in this fashion, the carboxyl termini of the 442 ORFs used for the bank were PCR amplified using genomic DNA from the Lp02 strain, purified using a Qiagen 96 sample purification kit (QIAquick® 96 PCR Purification Kit, Cat#28181) digested with the appropriate combination of enzymes (noted in Table S1), and ligated individually into pZL204 before transformation into *E. coli*. Miniprep DNA samples of 2–4 isolates from each ligation were then screened for inserts and then electroporated into *L. pneumophila* Lp02*ΔsidC* using a multiwell electroporation system from Harvard Apparatus. One colony from each electroporation was purified and stored at −80°C until further use.

### Screen for fusions that rescue translocation defect of *sidCΔ100*

Translocation efficiencies of each carboxyl terminal fusion were determined for 85–96 clones per experiment. For each assay, bacteria were isolated on CYE plates and grown in AYE broth in 24-well plates with shaking at 37°C until bacteria were found to be highly motile (A_600_ > 3.2). Included in each experiment were positive (Lp02*ΔsidC*/pZL199-SidC^+^) and negative (Lp02*ΔsidC*/pZL204-*sidCΔ100*) control strains for measuring SidC translocation, and translocation efficiencies were determined in at least triplicate wells for each candidate and both controls. The bacteria were then diluted into RPMI1640 + 10% fetal calf serum and introduced onto bone marrow-derived A/J macrophage monolayers plated at a density of 5 × 10^4^ cells well^−1^ in 96-well plates with optically clear bottoms (Costar, cat #3603) at 37°C. Challenge of macrophages was initiated by centrifugation onto cell monolayers at 1000× r.p.m. for 5 min in an Eppendorf table top centrifuge, followed by 1 h incubation at 37°C, 5% C0_2_. The monolayers were then washed three times in PBS, fixed in 3.7% paraformaldehyde at room temperature for 10 min, permeabilized in 0.1% Triton for 10 min at room temperature and then blocked in PBS containing 5% goat serum. The monolayers were then incubated for 1 h at room temperature with a mix of rat anti-*L. pneumophila* serum diluted 1:10 000 and rabbit anti-SidC diluted 1:500 in PBS containing 5% goat serum. The wells were washed three times in PBS, and probed for 1 h. with 1:1000 dilutions of Texas Red conjugated anti-rat IgG and FITC conjugated anti-rabbit IgG in PBS contain 5% goat serum. After washing wells three times with PBS, 100 μl of PBS was added to each well before image analysis.

To determine the efficiency of SidC translocation, images of monolayers on microtiter wells were captured and analysed with a Molecular Devices ImageXpress. Four images from each microtiter well were captured with a Nikon 20× plan-Apo lens using the Texas Red and FITC filter sets to identify bacteria and exported SidC respectively. Thresholds were set for the captured images from each channel for each of the four fields, and the images processed in this fashion were overlayed using MetaExpress software. To determine the translocation efficiency, the number of bacteria B = the number of Texas Red-positive particles (each corresponding to an individual bacterium) in all four fields, while the number that were positive for translocation F_B_ = number of FITC-positive particles that were also Texas Red-positive. The absolute efficiency of translocation (E_a_) was determined by calculating the ratio E_a_ = F_B_/B. To determine the relative efficiency percentage (%E_r_) for each fusion, the mean translocation efficiency E_c_ of the positive full-length SidC control was determined for 3–6 wells, to allow the determination %E_r_ = (100)(E_a_/E_c_). All translocation percentages are expressed as %E_r_ to allow comparisons between experiments performed on different days.

For initial screening of the bank, all fusions were analysed as single samples, with the assays performed on three separate days to obtain triplicate data. Fusions that were observed to give a %E_r_ that was larger than 2× that of the negative control on at least one occasion were selected for further analysis, and the nucleotide sequences of each of these positive clones was determined. The sequenced plasmids were transformed into Lp02*ΔsidC*, and then assayed again in triplicate. For each candidate, translocation assays on the freshly transformed strains were performed in triplicate, repeating assays 2–4 times. If the %E_r_ < 45%, then data were subjected to a Two-Sample *t*-test assuming equal variances. The resulting *P*-values are displayed in Table 1 and, unless noted, if the translocation efficiency relative to the negative controls resulted in *P* ≤ 0.05, then the fusion was deemed to have a translocation signal.

### Determination of *z* score for the presence of a Glu-rich motif at carboxyl termini of IDTS

*Z* scores were calculated to assess the possibility that the enrichment of EE, ExE and ExxE in the pool of 182 genes with known signals was due to chance. The expected frequency of each of these sequences was determined by construction of random sets of 18mers (the length of the region containing most of the predicted signals) based on the amino acid composition of the 18-residue signal region (−8 to −25 relative to the carboxy termini) of the whole genome. Sets of 182 18mers were used for the known pool. Sets of the number of proteins in the genome minus 182 represented the proteins in the genome not known to have signals. The number of occurrences of each of the three target motifs was counted in each set. Randomizations were repeated 10 000 times to calculate the expected mean and standard deviations. Duplicate runs of 10 000 iterations produced almost identical results. These numbers were compared with the observed occurrence of the motifs in the two sets of genes. The *z* score is the number of standard deviations separating the mean from the expected value.

### Bioinformatic identification of a translocation signal

A table of 182 putative Icm/Dot substrates was generated that included: (i) proteins identified in this work, (ii) proteins that had been previously identified as being translocated, (iii) proteins hypothesized to be translocated based on eukaryotic sequence similarities and (iv) paralogs of members of each of these three classes (Table S2). The carboxyl terminal 75 amino acids of each protein was then subjected to motif searching using the BLOCKS server (http://blocks.fhcrc.org/blockmkr/) using the Gibbs and Motif alignment algorithms (Henikoff *et al*., 1995). Using this strategy, the two different algorithms identified overlapping sequence blocks in each of approximately 50 of the members of the library. For most of the regions identified, the amino acid blocks were found to be in the carboxyl terminal 30 amino acids of each protein. Therefore, the BLOCKS search was then repeated on these 50 members, this time limiting the blockmaking routine to the carboxyl terminal 30 amino acids. From these 50 proteins, a consensus COBBLER sequence was determined (Fig. 3), which was primarily rich in Glu residues. Then the 182 members of the library were scanned visually for six amino acid regions in their carboxyl termini that had at least two Glu residues or a Glu and Asp. Ninety-eight members of the library were found to have such Glu-rich regions in the carboxyl termini (Table S3). A new search library was then made from the members shown in Table S3, in which sequence starting at the first Glu and extending to the end of the protein, and subjected to a round of BLOCK searching. The consensus sequence shown in Fig. 3 was derived from this search.

### Identification of potential translocated substrates having E Blocks

The consensus sequences showed a preference for Glu in the carboxyl termini of translocated substrates. To obtain better-defined sequences that would allow blast search identification of proteins that have this motif, the 98 carboxyl terminal sequence bank (Table S3) was blast searched against itself, and a sequence similarity tree was built to determine if there were subgroups of similar sequences. The tree constructed had four major subgroups, each with slightly different motifs that were rich in glutamates. The four motifs as well as two examples from each of the consensus motifs sequences described in Fig. 3 (EExEENxNS, EExETNS, EEVETNS, ExSEKMk, EEEEQEKN, EKSxDLqn, EKEEDKxT, EDxETxNST) were then used to blast search against the *L. pneumophila* Philadelphia 1 genome sequence, and proteins that were found to have sequence similarity in their carboxyl termini were analysed further. From this group, proteins that were clearly associated with housekeeping functions were discarded. Several of those discarded proteins were involved in nucleic acid biogenesis, perhaps because these proteins have largely acidic carboxyl termini. Proteins already in the search bank were also discarded, and the new proteins were subjected to blast searching using the Nonredundant Database to obtain the annotations described in Table 3.

### Adenylate cyclase translocation assays

12-tetradecanoyl phorbol 13-acetate treated U937 cells were incubated in 24-well plates and challenged with *L. pneumophila* strains at MOI = 1.0. CyaA protein expression in *L. pneumophila* was induced by growing bacteria to A_600_ = 2.0, adding 100 μM IPTG, and growing until the bacteria were in post-exponential phase and largely motile. To assay for cyclase activity, after 1 h. incubation with bacteria, U937 cells were washed three times with PBS and extracts were prepared by adding 200 μl of lysis buffer (50 mM HCl, 0.1% Triton X-100) and incubating on ice for 10 min. Lysates were collected and boiled immediately for 5 min, then neutralized by addition of 12 μl of 0.5 M NaOH. The extracts were precipitated by adding 400 μl of cold 95% EtOH (65% final) and incubated on ice for 5 min. Insoluble material was removed by spinning samples in a microfuge for 5 min at 4°C at 13 000 r.p.m. Supernatants were dried under vacuum and resuspended in assay buffer. The cAMP concentration was measured using Amersham Biotrak cAMP ELISA Kit. Data were normalized to amount of fusion protein, determined by Western blotting that was present in each *L. pneumophila* strain analysed. Western blotting was performed using either rabbit anti-SidC antiserum or rabbit anti-CyaA, as described (Luo and Isberg, 2004), on gel fractionated extracts of *L. pneumophila* strains that were used to challenge U937 cells. Data were expressed as fmoles/well[fusion protein], using the relative steady state levels of fusion protein expressed in each strain as the normalization value.

## Notes

**Table S1.** Bank of C-terminal fragments. **Table S2.** Pool for block searches. **Table S3.** Location of E Blocks. **Table S4.** blast Results for E BLOCK Query. **Table S5.** C termini used in Fig. 4A. **Table S6.** Carboxyl termini of translocation competent fusions to SidC that have identifiable E Block. **Table S7.** ExxE motif in E Block proteins.
