# Supplementary material for: The E Block motif is associated with Legionella pneumophila translocated substrates
Source: Cell Microbiol. 2010 Nov 3;13(2):227–45. doi: 10.1111/j.1462-5822.2010.01531.x (PMC3096851; doi:10.1111/j.1462-5822.2010.01531.x)
Supplement: Supplementary file 1 — Table S1. Bank of C-terminal fragments. Table S2. Pool for block searches. Table S3. Location of E Blocks. Table S4. blast Results for E BLOCK Query. Table S5. C termini used in Fig. 4A. Table S6. Carboxyl termini of translocation competent fusions to SidC that have identifiable E Block. Table S7. ExxE motif in E Block proteins. [file cmi0013-0227-sd1.pdf]

Supplemental Table 1: Bank of C terminal fragments

ORF	Annotation	5'EndoR	3"EndoR	PRIMER_LEFT_SEQUENCE(5')	PRIMER_RIGHT_SEQUENCE(3')
lpg0008	ORF	BamHI	XbaI	ctagGGAtccctgTTTgccGCTaaaGTCg	CCTCTCTAGAGGGCAAAAGATATGGCTGC
lpg0012	ORF	BamHI	XbaI	ctagGGAtccCTtGGGgatGGGaaaTTTgat	CCTCTCTAGAAGCGCTTCATGATCACCTCT
lpg0016	ORF	BamHI	XbaI	ctagGGAtccTGTtttACTgtgGAGgcgG	CCTCTCTAGATCCATTATCAAAACCGGCA
lpg0017	ORF	BamHI	XbaI	ctagGGAtccCTtGCCggtTTTgatAATgga	CCTCTCTAGATTAAAATCACGCCACCAGG
lpg0030	ORF	BamHI	XbaI	ctagGGAtccTTTgcgCCTaaaATAcccC	CCTCTCTAGACGGTGGCGGATTCTAACTT
lpg0033	ORF	BamHI	XbaI	ctagGGAtcccaaACCattCCTtttCGCa	CCTCTCTAGAGGGGACTGAGAAAGACACCA
lpg0038	RIKEN cDNA 4921520G13 gene	BglII	XbaI	ctagAGAtctCcaAACacgCCTttgCATct	CCTCTCTAGAATCAGGGCGCTTTACCTTG
lpg0041	ORF	BamHI	XbaI	ctagGGAtccTTCaggGTAaccGTAcgcA	CCTCTCTAGACCAGCGGGTAGACCAGATT
lpg0042	ORF	BamHI	XbaI	ctagGGAtccCtgGAAtctCATgccAGTttg	CCTCTCTAGACGCATTGTTGGTTAGTGGC
lpg0054	ORF	BamHI	XbaI	ctagGGAtccCTGactGGAggcTTCatgATAa	CCTCTCTAGAATCCAGTCTTCTTGCGCCT
lpg0056	ORF	BamHI	XbaI	ctagGGAtccCTtGATgatGAAtctTGCggg	CCTCTCTAGACTCGGCTCATTTTGGAGGT
lpg0059	ORF	BamHI	XbaI	ctagGGAtccCTgCGAcaaGCCaagTACgaa	CCTCTCTAGATGATTGCGGAGTCAAGGAG
lpg0062	ORF	NotI	XbaI	ctgaGCGgccGCcGCAtggCAGgaaGTTaga	CCTCTCTAGACGTAAAGGCATGGTTGCTG
lpg0065	ORF	BamHI	XbaI	ctagGGAtccCTtGGTggaACAccaGAAatcA	CCTCTCTAGACAATGAAAGAACAGCGCCA
lpg0080	ORF	BamHI	XbaI	ctagGGAtccCGCgatCCTctgGTTggaTT	CCTCTCTAGAATCCAAGGCCACAATCCTC
lpg0086		BamHI	XbaI	ctagGGAtcctggCATcagAAGaagCAGg	CCTCTCTAGACCAGGATGTGAGCGAAGAA
lpg0081	ORF	BamHI	XbaI	ctagGGAtccCTtAACaagCTGatgGCTcgc	CCTCTCTAGATTTGCTGAGGCGATTTGAC
lpg0090	ORF	BamHI	XbaI	ctagGGAtccCTAtacGATgcgCTGgaaGC	CCTCTCTAGAAAGTCATGCATCGGGTTGA
lpg0096	ORF	BamHI	XbaI	ctagGGAtccCGCccgACAgacTATgctGA	CCTCTCTAGAATTCTGGAATGGCCGGATA
lpg0101	ORF	BamHI	XbaI	ctagGGAtccCTTcttTCCgctTCAgaaCCC	CCTCTCTAGAAACCTGCATACCCCAGACC
lpg0107	hypothetical (membrane protein required for meiotic division?)	BamHI	SalI	ctagGGAtccCtcTGTgtgGCCaaaACCat	CCTCGTCGACTGATCCATTCCTGCATCGT
lpg0110	ORF	BamHI	SalI	ctagGGAtccCTAgcgCTTgcgGTAaagATT	CCTCGTCGACTGTCTGCAACACGCAAAAG
lpg0111	squalene and phytoene synthases (farnesyl-diphosphate farnesyltranst	BamHI	XbaI	ctagGGAtccCgtGCCgtcCCTcttTTGtt	CCTCTCTAGAGCACTGCCGTTCAATTTGT
lpg0112	hypothetical protein	BamHI	SalI	ctagGGAtccctcTGTgagCCAggaACCa	CCTCGTCGACTCGGGCAAAAATAATCGGT
lpg0126	ninein (GSK3B interacting protein)	BamHI	XbaI	ctagGGAtccAGCaggTGCcaaACAgttG	CCTCTCTAGAGAACCGTCGAGCTTCACAA
lpg0130	ORF	BamHI	XbaI	ctagGGAtccCTGccaGGTtcaGAGggaATC	CCTCTCTAGAACCCGTATTACTGTCGGGC
lpg0135	SdhB	BamHI	XbaI	ctagGGAtcctccGAAgatCCCtcgAATc	CCTCTCTAGATGTTCACGGGGTAAGTCCA
lpg0140	ORF	BamHI	XbaI	ctagGGAtccCGGatgGGCgaaAAAgaaAA	CCTCTCTAGAGCGGTGAAATGGCAAAGTA
lpg0148	ORF	BamHI	XbaI	ctagGGAtccCTgGGAacgGCTcctGTTtat	CCTCTCTAGACAATTGAACGTTTCGGGTG
lpg0149	ORF	BamHI	XbaI	ctagGGAtccTTAgcaTATgcaGCAgccC	CCTCTCTAGATTAAATCCCCCAATGGGAA
lpg0151	ORF	BglII	SalI	ctagAGAtctCggATTgacAAAtacCAAatcCAA	CCTCGTCGACGGCAGTGGCTGCGTAGTAA
lpg0154	ORF	BamHI	XbaI	ctagGGAtccCCTgagGCAactTTGcttGG	CCTCTCTAGAACGAAAAACCCCAAGGTCA
lpg0160	ORF	BamHI	XbaI	ctagGGAtccacgTTGgaaAGGgatCTGg	CCTCTCTAGAAATTCGCGCCAAAGAAAAC
lpg0170	probable ABC transport system permease protein	BamHI	XbaI	ctagGGAtccCatTCGcttGAGctcGCTtt	CCTCTCTAGATCACCAATCTTCACGCCTC
lpg0172	ORF	BamHI	XbaI	ctagGGAtccCTtCGTgccTTAtttGAAgcg	CCTCTCTAGACCGAAATTGATTGATAATAGACCA
lpg0181	ORF	BglII	XbaI	ctagAGAtctCTtTGGgggAATggaTGTttt	CCTCTCTAGAGCCCTCGCCTCGTTAGTAG
lpg0191	ORF	BamHI	XbaI	ctagGGAtccCATgccAAGgatAAAccgGA	CCTCTCTAGAGCCGGCTTGAAATTTTGAT
lpg0195	ORF	BamHI	XbaI	ctagGGAtccCgaGGAattGACttgCCGgt	CCTCTCTAGAAAAATGGAAGGCGAGATGG
lpg0196	ORF	BamHI	XbaI	ctagGGAtccCTtGACccaGCCtatCAGcaa	CCTCTCTAGATTATGCTGGAACTCAACCGA
lpg0198	hypothetical	BglII	XbaI	ctagAGAtctCacATTcgcCTGgaaAAAgc	CCTCTCTAGAGGAAAACTGCCAGTCCAAGA
lpg0208	serine/threonine-protein kinase	BamHI	XbaI	ctagGGAtccCCGatgGTCaagTGCgtaG	CCTCTCTAGAAGCAAGGTTATCGCTGCCT
lpg0209	ORF	BglII	XbaI	ctagAGAtctctagAGctagcGAGgggCTTgaa	CCTCTCTAGATATCACTCGCCAAAAAGCG
lpg0210	ORF	BamHI	XbaI	ctagGGAtccgcaGCAattATCaaaAATcgaA	CCTCTCTAGATCCAAGGTCAAGGCAAAAA
lpg0214	murein hydrolase exporter (LrgA family protein)	BamHI	XbaI	ctagGGAtccCTGccaAGCagcATTctcTC	CCTCTCTAGAACGGAACCACACAAGAGCA
lpg0227	ORF	BamHI	XbaI	ctagGGAtcccgaAGGggtTCTctgGAAa	CCTCTCTAGAGGGTAACTTGCCTGCTTCC
lpg0229	heme oxygenase	BamHI	SalI	ctagGGAtccAAAcccGGCcaaCTTacaG	CCTCGTCGACCCACCAGCTGATGCAACTAA
lpg0234	SidE	BamHI	XbaI	ctagGGAtccGCCcagGAAaaaGAAgcaC	CCTCTCTAGACCTTAATGGGCTACCGTGG
lpg0236	ORF	BamHI	XbaI	ctagGGAtccCTcGCTgacGAAtacCTGgaa	CCTCTCTAGATGCCCGTAATAAGTTGCCA
lpg0240	DNA repair protein	BamHI	XbaI	ctagGGAtccCTGcgcAACttgCTTgaaAAA	CCTCTCTAGAATGCATCAAGCGAACAGGT
lpg0246	ORF	BamHI	XbaI	ctagGGAtccTTTagcCGTatgCGTtggA	CCTCTCTAGAGCGGGAACCAATAACATCC
lpg0247	ORF	BglII	SalI	ctagAGAtctttgCTGatgGCAgctTACc	CCTCGTCGACCTTCGCTTTTATCCTGCCC
lpg0258	ORF	BglII	XbaI	ctagAGAtctCGTggtGATggaGATggcAG	CCTCTCTAGATGGATGAGCGCTTTTTACG
lpg0259	hypothetical protein	BamHI	XbaI	ctagGGAtccCatTATgaaCGCcacACCga	CCTCTCTAGAGGCCAATATGAATCAGCCC
lpg0264	unknown protein	BglII	XbaI	ctagAGAtctCTTgctTTCattAGCgccTC	CCTCTCTAGAGGCATCTGGGCTAACCATT
lpg0275	SdbA	BamHI	XbaI	ctagGGAtccCTTattTTTcttTTCggcGGG	CCTCTCTAGACGCTTTTTGGAATTGCATC
lpg0276	ORF	BamHI	XbaI	ctagGGAtccCTcTCGgtcAGCgatTTTgaa	CCTCTCTAGAGAGTCGAAACGCAAAACCA
lpg0282	conserved hypothetical protein	BamHI	XbaI	ctagGGAtccCTaGCGactGTGgctGAAgaa	CCTCTCTAGATTCCAAGCCCATAAAAGGC
lpg0284	ORF	BglII	SalI	ctagAGAtctCTTgaaGACgatGCTtcaCTGg	CCTCGTCGACCGGGGTTGGTTCTCATTCT

Supplemental Table 1: Bank of C terminal fragments

ORF	Annotation	5'EndoR	3"EndoR	PRIMER_LEFT_SEQUENCE(5')	PRIMER_RIGHT_SEQUENCE(3')
lpg0285	ORF	BglII	XbaI	ctagAGAtctCATggcAACataAAAgcgTG	CCTCTCTAGAAGTCGTGCTCCTTGTGCAG
lpg0292	ORF	BamHI	XbaI	ctagGGAtccCtgCAAagaCTGgttTGGaa	CCTCTCTAGAGCCTATTAGTGCGGCCAGT
lpg0365	ORF	BamHI	XbaI	ctagGGAtccCCCaacCCAcctACCtttG	CCTCTCTAGATCAATCCGGCTGTAGATGC
lpg0369	carboxylesterase/phospholipase	BamHI	XbaI	ctagGGAtccATAtggATGcatGGGttgG	CCTCTCTAGATGCTTTCCGCACAATAAGG
lpg0376	SdhA; GRIP coiled-coil protein GCC185	BamHI	XbaI	ctagGGAtccCTTgctTTCtgcAACcgtCAT	CCTCTCTAGAGTTGGCAAATCCGGAGAGT
lpg0380	ORF	BamHI	XbaI	ctagGGAtccCCAaccAGCttaCACgccAT	CCTCTCTAGAAGGCATGTACTATCGGGGC
lpg0390	ORF	BamHI	XbaI	ctagGGAtccCTTaaaGTCgggCCAgaaGG	CCTCTCTAGACGGGTTAATTCTGGGGATG
lpg0392	zinc metalloprotease (metal-dependent hydrolase)	BamHI	XbaI	ctagGGAtccCcaCCCgatGGGttaGTGaa	CCTCTCTAGATTATGGGCGATTAAGTCCG
lpg0393	ORF	BamHI	XbaI	ctagGGAtcctttCTGcagTGGccaAAAt	CCTCTCTAGACAAAAGCACCAAGGCAAAA
lpg0401	ORF	BamHI	XbaI	ctagGGAtccCTGcctAAAgcaTTTaccCATtt	CCTCTCTAGACCACAGAGAAGAGCGCATT
lpg0402	putative ankyrin-repeat containing protein	BamHI	XbaI	ctagGGAtccCCGcaaTTCgtgGCTtctTA	CCTCTCTAGATAAGTCGAGGGGGTTCGAG
lpg0403	similar to RIKEN cDNA 1700007B22	BamHI	XbaI	ctagGGAtccCGAttgACCgtgAATaccG	CCTCTCTAGATCCGATGCGAAAACATCAT
lpg0431	ORF	BamHI	XbaI	ctagGGAtccCccACGaatTTTacgGCAttt	CCTCTCTAGAAAAATGAGCATGGCAACCA
lpg0436	ORF	BamHI	XbaI	ctagGGAtccCtgCGTgaaTTGatgAAGgc	CCTCTCTAGATGCCAAGAAACGCCAAATA
lpg0437	ORF	BamHI	XbaI	ctagGGAtccCATcatGCTggaGCGcatAA	CCTCTCTAGATAGGGAATGGGAACCCAAA
lpg0439	ORF	BamHI	XbaI	ctagGGAtccggcACCtcaATCgcaGTTa	CCTCTCTAGACAAGTGGGTACGCATGGTT
lpg0468	lipase A	BamHI	XbaI	ctagGGAtccCCCttgCACggtTGGttaGA	CCTCTCTAGATTGAGTGCTGCCAAAGACC
lpg0483	ORF	BamHI	XbaI	ctagGGAtccCTAgacGCTcttCGTgggATT	CCTCTCTAGACCATGGTGTCTGTCGAAGG
lpg0502	phosphatidylcholine hydrolyzing phospholipase	BamHI	XbaI	ctagGGAtccCCCgggGGTggtTGTaatAG	CCTCTCTAGATTTTGGATTTGGTCCTATCCC
lpg0515	protein of ancient origin (eukaryotic?) (CG14688 gene product)	BamHI	XbaI	ctagGGAtccCTTgctGAGcagGATattGCC	CCTCTCTAGAATGGCAACCCAAATCCAAT
lpg0516	conserved hypothetical protein	NotI	XbaI	ctgaGCGgccGCTgcaAGCgagAAGtcaATCct	CCTCTCTAGATGGCACGACAAATTTGGAT
lpg0518	ORF	BamHI	XbaI	ctagGGAtccCGGctGGTttgGCAttaGT	CCTCTCTAGAAAAGACAACAGGCGGGATT
lpg0519	ORF	BamHI	XbaI	ctagGGAtccgcaGCAattGTTtcaACAgc	CCTCTCTAGAGTAGCCTCGTGTATGGCGA
lpg0525	unknown virulence protein	BamHI	SalI	ctagGGAtccCTCggcGATatcGAAatcAAA	CCTCGTCGACTGCATCAATGGACCCAAAC
lpg0541	protein product; similar to probable membrane protein YdgA of Escher	BamHI	XbaI	ctagGGAtccCTCctgGGTcaaGCCagtTTT	CCTCTCTAGACATTGCCACGTTTCTCACC
lpg0621	SidA	BamHI	XbaI	ctagGGAtcccgcGCTgagCAAttaAAAa	CCTCTCTAGATCGCTGAATGCTTGATCGT
lpg0625	expressed protein	BamHI	XbaI	ctagGGAtccaaaGCCaagGAAatgGCTg	CCTCTCTAGAAACAGACGAATGCGGAATG
lpg0634	ORF	BamHI	XbaI	ctagGGAtccCTaGCTttaAGTcaaGCCcgc	CCTCTCTAGACGTAACAGTCTTGATTTTCATTCG
lpg0642	ORF	BamHI	XbaI	ctagGGAtccCGAggtTATgcaGGCgaaCA	CCTCTCTAGATTGCCAACTCATCTGGAGC
lpg0645	partial RtxA	BamHI	XbaI	ctagGGAtccCATcatGAAatgGCGcaaGA	CCTCTCTAGATCGCGAGGATATTTTCCAA
lpg0685	Fe-S oxidoreductase (2-methylthioadenine synthase)	BglII	XbaI	ctagAGAtctTATcagCAGgtcGAAtggC	CCTCTCTAGATCGCAAAATTTGGAGGACA
lpg0693	LigA (L. pneumophila infectivity gene) (interaptin)	BamHI	XbaI	ctagGGAtccCTTccaGAGcaaTGCcaaAG	CCTCTCTAGACGGGATTTAATGTTGTGCG
lpg0695	hypothetical (inversin? alpha- or delta- latroinsectotoxin) (ankyrin rep	BamHI	XbaI	ctagGGAtccCTCgaaGACaggCTGggaAAT	CCTCTCTAGACATTGTTTGGGGAAAACCA
lpg0696	ORF	BamHI	XbaI	ctagGGAtccCTAacaGGCcatGTGgaaACA	CCTCTCTAGATGCTCGGTAATCTTCCTGG
lpg0716	ORF	BamHI	XbaI	ctagGGAtccTGCgttGTTaacCTGaccC	CCTCTCTAGATTTTGGGTCAAAGACACGC
lpg0717	small ORF (153aa)	BamHI	XbaI	ctagGGAtccccaTTGccgCAGaatAAAa	CCTCTCTAGAGCAGCGCACATTAGAAAGC
lpg0733	ORF	BamHI	XbaI	ctagGGAtcctggGTAgatGCAacaGGGa	CCTCTCTAGACAGGCTGCACCAGGAATAA
lpg0747	hypothetical	BamHI	XbaI	ctagGGAtccCcaATGgccAATttcACAgg	CCTCTCTAGATGAGCAATCAAGGGTCGAG
lpg0748	LPS biosynthesis protein (pseudaminic acid biosynthesis and flagellin a	BamHI	XbaI	ctagGGAtccCTtGGAgaaAATgaaGCGgaa	CCTCTCTAGAATCCCTCCAGATGCACTCC
lpg0764	hypothetical	BamHI	XbaI	ctagGGAtccCTTtatGGCtttCACacgGGA	CCTCTCTAGAAGCGCATAATTTGCACCTG
lpg0765	tetratricopeptide repeat domain 8	BamHI	XbaI	ctagGGAtccCcgGAAgcaATTaaaTGCgatA	CCTCTCTAGAGTAGTTGCACAGGCCGAAA
lpg0766	hypothetical	BamHI	XbaI	ctagGGAtccCTTgccAAAaccGAGgtgATA	CCTCTCTAGACAGGAGAAGAACGGCACAA
lpg0767	hypothetical	BamHI	XbaI	ctagGGAtccCggCAAtatCAGcagAGCga	CCTCTCTAGAACCAACATCCCGTGCAGTA
lpg0770	hypothetical	BglII	XbaI	ctagAGAtctCCAaagCAAccaTGGacaA	CCTCTCTAGATTGCAGTGCATTATAGGGGA
lpg0771	hypothetical	BamHI	XbaI	ctagGGAtccGCAacaACAaacGGTcagG	CCTCTCTAGAATAACCCAACCCAATCCCA
lpg0774	hypothetical (Legionella pneumophila)	BamHI	XbaI	ctagGGAtccGGTgatCCAcctGGGtttC	CCTCTCTAGAAAGCAAAGGGAATGTGGAGA
lpg0788	ORF	BglII	SalI	ctagAGAtctCTGcaaACActtTCAtcaACGc	CCTCGTCGACCGCTCCAGGTACCGTAAAAA
lpg0796	ORF	BamHI	SalI	ctagGGAtccCTtCCTtacAATcaaAATggaACTtt	CCTCGTCGACGGTGGCGGAATTGGTAGAC
lpg0815	ORF	BamHI	XbaI	ctagGGAtccATGgttGGGgatGAAcctG	CCTCTCTAGATGAATGGTCAGCAGCAACA
lpg0827	agglutination protein, COG1538: membrane protein	BamHI	XbaI	ctagGGAtccCTTgctTATgttGCCtctGCC	CCTCTCTAGAGGTAGGATTGCGCCAATTT
lpg0855	signal peptide peptidase SppA (protease IV) (SohB protein)	BamHI	XbaI	ctagGGAtccCaaGATaaaACCaagCGCcc	CCTCTCTAGACCTATGGAATGCTGCCTGA
lpg0871	ORF	BglII	XbaI	ctagAGAtctctagCAAcagCTGgacTGCgat	CCTCTCTAGAAGCTCATCTTCCGGATTGG
lpg0880	ORF	BamHI	XbaI	ctagGGAtccgagCCCaccTCTcaaGACc	CCTCTCTAGATTAACTGAGATGGCGCCTG
lpg0889	acyltransferase (endonuclease?)	BamHI	XbaI	ctagGGAtccCTAaacTGGcaaTCCcttTGC	CCTCTCTAGATCAGCGACTCTGAATTGGC
lpg0898	hypothetical	BamHI	SalI	ctagGGAtccCTtGCTgttTCGcgtGTCata	CCTCGTCGACTGGAAGAACAAGCCGTGAG
lpg0901	hypothetical protein NMA0899	BamHI	XbaI	ctagGGAtccgcgTCAcaaTTAccgGAAg	CCTCTCTAGACACCGGCCTCCTGATTTAT
lpg0902	ORF	BamHI	XbaI	ctagGGAtccataAATcagGAGgccGGTg	CCTCTCTAGAAATGGTAAGCCCAAACCCA
lpg0921	ORF	BamHI	XbaI	ctagGGAtccGGAgggCAGtacCTGgagA	CCTCTCTAGACGCAGGCAGGAAGCTATTT

Supplemental Table 1: Bank of C terminal fragments

ORF	Annotation	5'EndoR	3"EndoR	PRIMER_LEFT_SEQUENCE(5')	PRIMER_RIGHT_SEQUENCE(3')
lpg0926	ORF	BamHI	XbaI	ctagGGAtccCTcACCaaaGGCtatCCCgac	CCTCTCTAGAATCATTGATGAAGCGCCAA
lpg0940	LidA (translocated substrate of Dot/Icm system) (Uso1 protein)	BamHI	XbaI	ctagGGAtccGATgcgCATtacGTGataGGT	CCTCTCTAGATTTGATGATGAGGTGGGGA
lpg0941	ORF	BamHI	XbaI	ctagGGAtccCtaGCAgagCGTccaTCCac	CCTCTCTAGAGCATGATTGGAGCCGTTTT
lpg0944	ORF	BamHI	XbaI	ctagGGAtccCTTcaaCCCccaCAGcaaTTA	CCTCTCTAGAATGGACCGGCCTGAGATAG
lpg0956	hypothetical protein	BamHI	XbaI	ctagGGAtccCTCctgGAGgttGAAaccGAT	CCTCTCTAGAAATAGCCAACGCTATGCCC
lpg0963	ORF	BamHI	XbaI	ctagGGAtccCTTttaTTCggaGCCgctTTT	CCTCTCTAGATCTTGGCCAACAATGGTGT
lpg0968	ORF	BamHI	XbaI	ctagGGAtccGCTcaaCAAtgtGATgccC	CCTCTCTAGAGTATGGTGGTGCATTTCGC
lpg0969	ORF	BamHI	XbaI	ctagGGAtcctcgGAAtgtAGGaagTTGgg	CCTCTCTAGAAGTGTTTTCTGGCGTGCAG
lpg0971	ecto-ATP diphosphohydrolase II (ectonucleoside triphosphate diphosph	BamHI	XbaI	ctagGGAtccCGTgtgATGgatATTggcGG	CCTCTCTAGAGAGAGGAGTCGAACCTCCG
lpg0974	ORF	BamHI	SalI	ctagGGAtccCTTtggGAAatgAAAcccCAC	CCTCGTCGACGCGTCATTCTGAAGCTGGA
lpg0981	ORF	BamHI	XbaI	ctagGGAtccCgtGATgtgCCAtccTCCtg	CCTCTCTAGATTCTGCGCTTTCCAGTGAT
lpg1005	LvrA	BamHI	XbaI	ctagGGAtccCAGcaaCTCgctGAGgctTT	CCTCTCTAGATCTTTGATGCTGTCAGCCC
lpg1016	unknown Legionella protein (putative secreted protein?)	BglII	XbaI	ctagAGAtctCATgccACCcagATTttgA	CCTCTCTAGAGATCCGATCATGCCCTTCT
lpg1066	ORF	BamHI	XbaI	ctagGGAtccTTGactTGGccaTTGgttC	CCTCTCTAGACCCCCTTGTCAGCCATTAT
lpg1073	ORF	BamHI	XbaI	ctagGGAtccCTaAATggaATTccgTCTggc	CCTCTCTAGAATGCGGTTGTTGATTTGGA
lpg1083	conserved hypothetical protein	BamHI	SalI	ctagGGAtccTTTaccGACgatTTCctgC	CCTCGTCGACTCAACCGGGGGATAAACAT
lpg1101	ORF	BamHI	XbaI	ctagGGAtccCTtGAAaggATGcgcTATaaaAA	CCTCTCTAGATCAACCAATAAAATGCCGC
lpg1102	ORF	BglII	XbaI	ctagAGAtctGCCgaaAAAgacGCAcatA	CCTCTCTAGATGCGGCATTTTATTGGTTG
lpg1104	hypothetical protein	BamHI	XbaI	ctagGGAtccCTTcccCAAatcATTgcaGAA	CCTCTCTAGATGACGCATTGGATCATGTG
lpg1106	ORF	BglII	XbaI	ctagAGAtctCctGGCaacAACcctCTCaa	CCTCTCTAGATGCCTTTGTTTTTCCAGCA
lpg1108	putative lipase	BamHI	XbaI	ctagGGAtccGACaacAACacaGGGacgG	CCTCTCTAGACAAAACCCATGAATGGACG
lpg1109	ORF	BamHI	XbaI	ctagGGAtcctggGCTtctGTAcctGCTg	CCTCTCTAGAATCGGGGGCAGTTATTGTC
lpg1110	ORF	BamHI	XbaI	ctagGGAtccCttGAAatcAAAgatTCAgcaGG	CCTCTCTAGATGGAACGCTCAACACCAAT
lpg1111	ORF	BamHI	XbaI	ctagGGAtccACTgtaCTGgatGAAaacACGa	CCTCTCTAGACGCCCGACATTTACAACAG
lpg1116	chitinase (exochitinase) aminosugars metabolism?	BamHI	XbaI	ctagGGAtccggcGGTtccTTTtctCAAc	CCTCTCTAGACGCATTACCCTGTTTGGTG
lpg1120	hypothetical protein	BamHI	SalI	ctagGGAtccgctCGCtctGCAgttTTTg	CCTCGTCGACGGCGCATGAAAGGGATACT
lpg1121	ORF	BamHI	XbaI	ctagGGAtccgcaTTGgaaAAAgaaGCGa	CCTCTCTAGACATCGGATGGCTTGATGAG
lpg1129	ORF	BamHI	XbaI	ctagGGAtccTATgtcTTCatcGCGgcaT	CCTCTCTAGAGGGAACCCTTTATCACACCA
lpg1137	ORF	BamHI	XbaI	ctagGGAtccCTtGCTgtgGCTgtaACCcat	CCTCTCTAGATGCCTAATGCAATACATTCCC
lpg1145	ORF	BamHI	XbaI	ctagGGAtccCcaAAAcaaTCCataCGCgg	CCTCTCTAGAATCCCAGGAAACAATGGCT
lpg1148	ORF	BamHI	XbaI	ctagGGAtccCTtCCGcaaGGTgatGCTtac	CCTCTCTAGATGCCAGCGGTATCCATAAA
lpg1149	ORF	BamHI	XbaI	ctagGGAtccCctTCGctaTGGtggCCTtt	CCTCTCTAGATGGTGAAGCCGAAAATCAG
lpg1151	ORF	BamHI	XbaI	ctagGGAtcctatGATgaaGACacgGGGc	CCTCTCTAGACAACGCTAATGGCGAACTG
lpg1152	ORF	BamHI	XbaI	ctagGGAtccCTgGCTcttCCTgaaACCcaa	CCTCTCTAGAAAGCATGGGAAGAAGTGGG
lpg1154	ORF	BamHI	XbaI	ctagGGAtccCATcggGATgaaGAAgacGC	CCTCTCTAGAACAAACAAACTGGTCGGCA
lpg1157	lipase B (conserved hypothetical protein-putative ATP/GTP-binding pro	BamHI	XbaI	ctagGGAtccCTcAAGgctTATgctTACtccCC	CCTCTCTAGAAAGCCAATACTAGCCGCTGA
lpg1158	ORF	BamHI	SalI	ctagGGAtcctggTTGataATTggcATGaaa	CCTCGTCGACCCGTTGCTCGTCTATGCTC
lpg1162	ompA-like transmembrane domain protein	BamHI	XbaI	ctagGGAtccCctTTTgtcCATgctGGTgg	CCTCTCTAGAGGCCAAATACTTGACCCCA
lpg1166	hypothetical protein	BamHI	XbaI	ctagGGAtccCTAaaaACCcggGTCaatGAA	CCTCTCTAGATCCTTGGCTTCCTCAACAA
lpg1183	ORF	BamHI	XbaI	ctagGGAtccCTTgcaCAGagaAAGaacCCG	CCTCTCTAGAGAAAAGCAGGATATTGGCGA
lpg1189	ORF	BamHI	XbaI	ctagGGAtccactGACtggCTGgatGTGc	CCTCTCTAGAAAGGGCAAGGCCATAAGTG
lpg1227	unknown	BamHI	XbaI	ctagGGAtccACAattGGGgttTCTtgcG	CCTCTCTAGACCTCCTAAGGGGCAGGTC
lpg1233	ORF	BamHI	XbaI	ctagGGAtccCTgACTcctGATggcCAAcct	CCTCTCTAGACCGACGCGTTTAGAGTCAA
lpg1238	ORF	BamHI	XbaI	ctagGGAtccGATggcAATataACCccgC	CCTCTCTAGAAGGCATTTTTCTGGCTTGG
lpg1259	LvrA	BamHI	XbaI	ctagGGAtccCTAaggCGCatcAGTcatCAG	CCTCTCTAGACAGCGTAGGCGGTAACTCA
lpg1263	ORF	BamHI	XbaI	ctagGGAtcctttGCAaagCTTctaACAaaaA	CCTCTCTAGATTCAAAAATGATGGATTGGC
lpg1265	ORF	BamHI	XbaI	ctagGGAtcccctGCAcctAAAtcgCCTt	CCTCTCTAGAGAGGCAGCCCTATACCCAA
lpg1268	ORF	BamHI	XbaI	ctagGGAtccCCAgtgGTTgagCAGggtGT	CCTCTCTAGATGGCGGGGTCCATACTATT
lpg1273	ORF	BamHI	XbaI	ctagGGAtccCTaAAGtgtTTCacaACGccg	CCTCTCTAGACCAGAAACGGCAAGAATGA
lpg1290	hypothetical protein	BamHI	XbaI	ctagGGAtccCgcTTAgctAGGcatTGGgc	CCTCTCTAGAGCAAAGACTGCGACCGATA
lpg1312	erythrocyte membrane-associated antigen	BamHI	XbaI	ctagGGAtccCtgGAGcatGTAaatAGCctgG	CCTCTCTAGATTCCATTACCGAATCGCAA
lpg1316	ORF	BamHI	XbaI	ctagGGAtccCTtGGCttcCAAtacCACacc	CCTCTCTAGACCTTTACCATCGCCTGGAT
lpg1317	ORF	BamHI	XbaI	ctagGGAtccCCTggaGCAccaGAAcatGG	CCTCTCTAGACGGCGCCTCGTAATAATTT
lpg1318	ORF	BglII	XbaI	ctagAGAtctctagTGGgatCATgctGGGatt	CCTCTCTAGAGTGGCGATACGTCACTTGC
lpg1325	siderophore biosynthetic enzyme FrgA	NotI	XbaI	ctgaGCGgccGCCaccACAcacCCAtccATT	CCTCTCTAGATATCGATCAGCTGCCAAGC
lpg1328	conserved repeat domain protein	BamHI	XbaI	ctagGGAtccCTaAATccaGGTtgcCCTttg	CCTCTCTAGATCAGATGCGTGAGGATGGT
lpg1331	protease DO (periplasmic serine endoprotease)	BamHI	XbaI	ctagGGAtccCTTgtaGGTatcGGCtttGCG	CCTCTCTAGACTTTGGAGGATAGGCGGAA
lpg1354	ORF	BamHI	XbaI	ctagGGAtcccttTTTcatCACggaACGg	CCTCTCTAGAGTATTGGAACCGGAATGGG

Supplemental Table 1: Bank of C terminal fragments

ORF	Annotation	5'EndoR	3"EndoR	PRIMER_LEFT_SEQUENCE(5')	PRIMER_RIGHT_SEQUENCE(3')
lpg1355	SidG, LepC, Vng0394c	BamHI	XbaI	ctagGGAtccCTgCTGaacCCCaaaCAAggt	CCTCTCTAGATGTGCTGGATTCTTTTTGGA
lpg1368	ORF	BamHI	XbaI	ctagGGAtccCTAatgATGgatACGgtcGCC	CCTCTCTAGATCAAGGGGATAGCGCTAAAA
lpg1387	ORF	BamHI	XbaI	ctagGGAtccCTAaagGGGcaaTTAgccAAG	CCTCTCTAGATGCCTGAAACTCAAGGCAG
lpg1406	glycosyltransferase (dolichol phosphate mannosyltransferase) (polypre	BamHI	XbaI	ctagGGAtccGTAgttGTGgggAGCcgaT	CCTCTCTAGACAGGACTTATGCCCCCAAT
lpg1426	hypothetical (serine protease?)	BamHI	XbaI	ctagGGAtccCTTttgGGAgatTTGcttGCTt	CCTCTCTAGAGCCCGCATTATTAACCAGG
lpg1436	ORF	BamHI	XbaI	ctagGGAtccaaaTACcttCCCattGCCg	CCTCTCTAGAATAGGGGGCATAAGCGTTG
lpg1449	ORF	BamHI	XbaI	ctagGGAtcccaaGGAaagCCAcgcAATa	CCTCTCTAGATTCCAGACTCTTCACCGGA
lpg1452	lipase A? (hydrolase, alpha/beta fold family), kraken-like (serine hydrc	BamHI	XbaI	ctagGGAtccCTcCCTgaaGGTgttGTCcct	CCTCTCTAGAGACAAGCAGCCTTTCTCCC
lpg1455	phospholipase C (phosphatidylcholine hydrolyzing)	BamHI	XbaI	ctagGGAtccCTcGACtcgGCTattAGCaagG	CCTCTCTAGATGGCGCATGATACAATGAA
lpg1465	ORF	BamHI	XbaI	ctagGGAtccCtcTCActtCAAatcAAAgctGC	CCTCTCTAGATAGATGCCCTGTGCGCTAT
lpg1481	ORF	BamHI	XbaI	ctagGGAtccCCCcatTTCggtCCAttaCC	CCTCTCTAGAAGGCTGCCAATGGAAAATC
lpg1482	ORF	BamHI	XbaI	ctagGGAtccCtcGGAgttGAAagaGCCaa	CCTCTCTAGAAGGCTTAAGGTCGGCAAGA
lpg1483	ovarian-specific serine/threonine-protein kinase	BamHI	XbaI	ctagGGAtccCctGACccgGAAttgGAAct	CCTCTCTAGAAACAACCCGGATTAGACGG
lpg1484	ORF	BamHI	XbaI	ctagGGAtccCTtAGAgagGCGctgCAAaga	CCTCTCTAGAGTGTTGGGTATTGCGGTTG
lpg1488	surface antigen of Plasmodium and Entamoeba	BamHI	XbaI	ctagGGAtccggaCTTtccTGGcttCCTg	CCTCTCTAGATGCTTGGGCAGCAAAAATA
lpg1489	ORF	BamHI	XbaI	ctagGGAtcctcaACTaaaGCAaaaGGGgaaA	CCTCTCTAGATGCAGAAACGGGCAAATAA
lpg1491	ORF	BamHI	SalI	ctagGGAtccCTgGCTctaTCTcccAGCagc	CCTCGTCGACTGGACATGACTTGCTTTCACA
lpg1496	ORF	BamHI	XbaI	ctagGGAtccCTAgcaGCTtggGAAagCAT	CCTCTCTAGAAGAAGCACCCTTTGCATTC
lpg1498	ORF	BamHI	XbaI	ctagGGAtccgcaGGGgccAAAtctATTc	CCTCTCTAGAATTGCCTAAAACTTGGGGG
lpg1525	CAAX amino terminal protease family protein	BamHI	XbaI	ctagGGAtccCTGcagCAAgctCTCaaaGAA	CCTCTCTAGAGGGGGCTATCATTGGTTTG
lpg1549	ORF	BamHI	SalI	ctagGGAtccgaaAGCagcAAAgagCCTg	CCTCGTCGACTTTTGCCATCATGCACTCC
lpg1551	ORF	BamHI	XbaI	ctagGGAtccCTCgaaCAAattCGAaaaACTga	CCTCTCTAGAATGGCGGTTCTGTTGATGA
lpg1563	ORF	BamHI	XbaI	ctagGGAtccCTcACCatgCGAgcaAGTacc	CCTCTCTAGACAGCAAACGAGTTGCTGGT
lpg1588	ORF	BamHI	XbaI	ctagGGAtccCTgCATttgAAGgagCAAacg	CCTCTCTAGAATACGCCAGGCTTTGGATT
lpg1598	ORF	NotI	XbaI	ctgaGCGgccGCTACAaacCCGgagGTCactC	CCTCTCTAGATCCTTGTTAGGGCCTCTGC
lpg1602	FLJ00180 protein	BamHI	XbaI	ctagGGAtccatgGTTgctGCTgctTTTg	CCTCTCTAGACGGTCTATTCCGAAGGCAT
lpg1621	ORF	BamHI	XbaI	ctagGGAtccTTTgttGGCtcgTCCtcaG	CCTCTCTAGATTTACTCAAGGCGCAATCG
lpg1622	ORF	BamHI	XbaI	ctagGGAtcctccATGgcaTTGagtGTGg	CCTCTCTAGAGTTATCAAAAGCCCCCGAA
lpg1627	putative secreted protein	BamHI	XbaI	ctagGGAtccAATtccCATcatCACacgG	CCTCTCTAGATGTGAAACGCAGAAGGGTC
lpg1633	ORF	BamHI	XbaI	ctagGGAtccCaaGCAgtcGCAaatCGCta	CCTCTCTAGATCGAGCACAAGTCAAACGA
lpg1639	ORF	BamHI	XbaI	ctagGGAtccCTtCTGgaaTTGggaCAAcga	CCTCTCTAGAGTTGCAGCAGGTTTAGGCA
lpg1642	SidB, RTX (repeat in toxin) cytotoxin	BamHI	XbaI	ctagGGAtccCTtCATtctCTTggtGCAggc	CCTCTCTAGACGGCAATCAGCAAACAAAT
lpg1644	ORF	BamHI	XbaI	ctagGGAtccTTGcctGTTcagTCAttcTTG	CCTCTCTAGACAACTTTTGCTTCGCGGTA
lpg1649	myo-inositol catabolism protein IolE	NotI	XbaI	ctgaGCGgccGCTaCTgagGCCaatGCTtttGA	CCTCTCTAGATGCTGTGTTTGATGCAATGA
lpg1654	ORF	BglII	XbaI	ctagAGAtctCTGaatCAAtctGCTgggGA	CCTCTCTAGAACGCAAATTCTTGCTGCAC
lpg1655	class 4 metalloprotease (zinc metalloprotease) (virulence metalloprote	BamHI	XbaI	ctagGGAtccCTGcctTTGcgaGCCtatACC	CCTCTCTAGAGGTTGTGAGCCGGAAAGTT
lpg1660	ORF	BglII	XbaI	ctagAGAtctctcACCtggCTCagtGCAt	CCTCTCTAGAGAGGTAAGGTTGGTTGGGC
lpg1665	hypothetical (lipoprotein)	BamHI	SalI	ctagGGAtccCgcGCTtacCCAcaaAATga	CCTCGTCGACTTTGTCGGCATGAGCAAAT
lpg1666	ORF	BamHI	XbaI	ctagGGAtccCTGcaaAATccaGAGttcGCA	CCTCTCTAGAAGTATGGTTTTGCCCTGCC
lpg1668	ORF	BamHI	XbaI	ctagGGAtccCatCAGgatGCAgtgAAGcg	CCTCTCTAGATCAGATTGAACGAAAGCGTG
lpg1670	ORF	BamHI	XbaI	ctagGGAtccCccAGGgcaAAAatgCTCtt	CCTCTCTAGACCCATAGAATCGATCACCG
lpg1683	ORF	BamHI	XbaI	ctagGGAtccCTAggcAATtgcGGGtctTAT	CCTCTCTAGAAATTGCCAAATACCGAGCC
lpg1684	ORF	BamHI	SalI	ctagGGAtccCTtGTAtccAGAcgcCATcgt	CCTCGTCGACCCCGCCCAGGTTTTTACTA
lpg1685	ORF	BamHI	XbaI	ctagGGAtccCTtGCTtatTTTgctGCGctt	CCTCTCTAGATCAGCCCTGCTTGATATGG
lpg1687	ORF	BamHI	XbaI	ctagGGAtccGCTttgCGCcatTTTatgA	CCTCTCTAGATGCCTGATAGACCGATGGA
lpg1689	23.7 kDa protein	BamHI	XbaI	ctagGGAtccCtcAGCtgcCATaaaTCTcagG	CCTCTCTAGAGGGAGGCGATTAATTCCTTT
lpg1692	ORF (SidC, SdcA homolog)	BamHI	XbaI	ctagGGAtccCTtGATgggACAcgaAAGgaa	CCTCTCTAGAGACGCGAACAGCAAATGAT
lpg1701	kinectin 1 (kinesin receptor)	BamHI	XbaI	ctagGGAtccCTGacaGCGgcaAATgaaGAA	CCTCTCTAGATGGGTAAGTTTGACGAGTTCC
lpg1702	Tpr	BamHI	XbaI	ctagGGAtccCTAaggAGGaatGAGcgcCT	CCTCTCTAGAAGTCCCGTTCTCCTCGCTA
lpg1718	huntingtin interacting protein B	BamHI	XbaI	ctagGGAtccCTGgctGCCtgtTATcttGG	CCTCTCTAGAAACAGCCCCAATCAGGTTC
lpg1728	unknown	BamHI	XbaI	ctagGGAtccCatTGGcgtGGAattGGTtt	CCTCTCTAGATGCCATGAGCAAAATGGAT
lpg1738	ORF	BamHI	XbaI	ctagGGAtccATTgccGGGcttTATgttC	CCTCTCTAGATTTCGTGCCTCCGATATGA
lpg1748	inositol-1-monophosphatase (extragenic suppressor protein SuhB)	BamHI	XbaI	ctagGGAtccCTtATTgctGTCaatGCTgcg	CCTCTCTAGATCTCAGCTTCGAGAGAGCG
lpg1751	ORF	BamHI	XbaI	ctagGGAtccCTTgtaGGGaagGATgagGGG	CCTCTCTAGATTACGGGGCAATGTTTCAA
lpg1752	ORF	BamHI	XbaI	ctagGGAtccCCCatgCTAgagTTTgccGA	CCTCTCTAGACGGGCGGAGTAAAAGTCAT
lpg1754	ORF	BamHI	XbaI	ctagGGAtccAAAataTTCtgcCTTgttCAGaga	CCTCTCTAGACTTTGGCAGCAAGCAAATC
lpg1755	transmembrane protein (protease?)	BamHI	XbaI	ctagGGAtcctttGAAgggGAAtggCTTc	CCTCTCTAGAAATTCTCCGGGGTTTCCTT
lpg1773	protein associating with small stress protein PASS1	BamHI	SalI	ctagGGAtcccccATTcgtATTtttGGGc	CCTCGTCGACTGATTTGCTCCTGTTTTTACGA

Supplemental Table 1: Bank of C terminal fragments

ORF	Annotation	5'EndoR	3"EndoR	PRIMER_LEFT_SEQUENCE(5')	PRIMER_RIGHT_SEQUENCE(3')
lpg1797	ORF	BamHI	SalI	ctagGGAtccgcgAAAgcaGTCgcaTATc	CCTCGTCGACATTGAGATCTCTTGGGCGG
lpg1798	ORF	BamHI	XbaI	ctagGGAtccCtgCATacaGCAgggGTTtt	CCTCTCTAGACAACACATTTTACCGCCCA
lpg1803	hypothetical	BamHI	XbaI	ctagGGAtccCCCtatGCAgaaTGGctgCT	CCTCTCTAGATTTAGCTTGGTAACGGGGC
lpg1817	ORF	BamHI	XbaI	ctagGGAtccCTAattGACggcCAAaccAA	CCTCTCTAGATACCTTGGCAATGCTCTCG
lpg1820	ORF	BamHI	XbaI	ctagGGAtccCTGatcCTGgtgGTGaggAA	CCTCTCTAGACGTCAATGCCCGAATATAGG
lpg1822	ORF	BamHI	XbaI	ctagGGAtccTTGtttCTTggaGCGgttG	CCTCTCTAGATCCTGTTGCATAAACGAGCTT
lpg1836	coiled coil domain protein	BglII	XbaI	ctagAGAtctTATgccAAAgaaCGCcaaA	CCTCTCTAGAGCAAATTTCGGGGAGGATA
lpg1851	ORF	BamHI	XbaI	ctagGGAtccCTGtcaTTTgaaTTGgtcGCA	CCTCTCTAGAGGTGGAATTATGGCTTGGG
lpg1881	ORF	BamHI	XbaI	ctagGGAtccaccAGGttaAAAcggCCTg	CCTCTCTAGAGCACCATTGCAGCAAAAAT
lpg1884	Microtubule binding protein, putative	BamHI	XbaI	ctagGGAtccCTGtctTTTgagCCTctcCGC	CCTCTCTAGACCGCACGTGATAAGCAATC
lpg1889	lipase (triacyglycerol lipase)	BamHI	XbaI	ctagGGAtccCTAcctGGCggtAAGgttGAC	CCTCTCTAGAAATAACGGCAATCCCCAAA
lpg1907	ORF	BamHI	XbaI	ctagGGAtccCTtGGCctgGCTcctTATacc	CCTCTCTAGACCATGTGCGAAAAATAGCG
lpg1909	ORF	BamHI	XbaI	ctagGGAtccCTaCCGcttGTTcaaAATgga	CCTCTCTAGACAGTGAAAAGGATGAGCTTGC
lpg1924	hypothetical protein	BamHI	XbaI	ctagGGAtccCAGataCACttcCCGcagC	CCTCTCTAGATCAAGCCCACCATGACATT
lpg1925	ORF	BamHI	XbaI	ctagGGAtccCttAGCaaaGCCcatCTGga	CCTCTCTAGAATTCACCCGGTGCAAGAAT
lpg1930	ORF	BamHI	XbaI	ctagGGAtccCAAgccGCGactGTTacaT	CCTCTCTAGAATTAAAATCCGAGGGTGCG
lpg1931	ORF	BamHI	XbaI	ctagGGAtccCTtCGAacgCATgtaATTgagC	CCTCTCTAGAATGTTGGTGTCCGGCTTTT
lpg1933	ORF	BamHI	XbaI	ctagGGAtccCTCcctCAAgatGTAatcGCC	CCTCTCTAGATGATTGGTGTGGCCTGTCT
lpg1947	hypothetical protein	BamHI	XbaI	ctagGGAtccCTcGACattAACcctGTCaacCT	CCTCTCTAGAGGGGAAGGCGTGATGTAAT
lpg1948	FLJ00180 protein	BamHI	XbaI	ctagGGAtccCttGGAgaaAAAtctGGGgc	CCTCTCTAGAACATCAAGCCTGGCCAAAT
lpg1949	hypothetical protein	BamHI	XbaI	ctagGGAtccgagGCGcagCAActaGTCa	CCTCTCTAGACGCATAGCTCCATGGAATG
lpg1950	guanine nucleotide exchange protein (RalF) (ADP ribosylation factor gu	BamHI	XbaI	ctagGGAtccCTTgacCGGttaGTGcaaAGC	CCTCTCTAGAGAGGCCATTTCACCCAGAT
lpg1953	ORF	BamHI	XbaI	ctagGGAtccCTgGAAgagTCCattGTGcga	CCTCTCTAGATGATGCTTGATGCACAGGA
lpg1958	FLJ00180 protein	BamHI	XbaI	ctagGGAtccCTgGAAttgGCAatgGCTttt	CCTCTCTAGAAAAATGGCACCACGTGAAA
lpg1959	ORF	BglII	XbaI	ctagAGAtctGTGcggAGGattCCTgaaG	CCTCTCTAGACCTCCAACGCGCTAATTTT
lpg1960	ORF	BamHI	XbaI	ctagGGAtccGGTgaaTTAttaCAAaatGAGccc	CCTCTCTAGATGCCTGATGAGTATGGGGA
lpg1961	ORF	BamHI	XbaI	ctagGGAtccCTtGGCaatCTGatgGATgct	CCTCTCTAGATCTCATGCCAAAACCCAAA
lpg1963	hypothetical protein	BamHI	XbaI	ctagGGAtccTTAtccGAAccgGAAaaaTG	CCTCTCTAGAAGATGCCCACTGATCCACA
lpg1964	ORF	BamHI	XbaI	ctagGGAtccgcgTTTtgtGAAattGAAcatT	CCTCTCTAGAATCAAGCTGATTGGGCCTT
lpg1965	hypothetical protein	BglII	XbaI	ctagAGAtctCTtCCGgttGTAacgCTTttg	CCTCTCTAGAGCCGCATCTATACGACCAA
lpg1966	ORF	BamHI	XbaI	ctagGGAtccCTcAGAtggTTGtgtCCCgaa	CCTCTCTAGATTCGATTTTATCTTTTGCCCA
lpg1969	putative	BamHI	XbaI	ctagGGAtccCagGAAatgGCGgaaAAAga	CCTCTCTAGACTGCCTTTAAATCAGCCCC
lpg1975	ORF	BamHI	XbaI	ctagGGAtccCTcAAAcctGATgcaACCcag	CCTCTCTAGATTGCGGCCGATTATATTGA
lpg1976	UVB-resistance protein UVR8	BamHI	SalI	ctagGGAtccCTtCAAtatAATcggCCGcaa	CCTCGTCGACTATAAGTGGGAGGGGGCTG
lpg1978	hypothetical Teichoic Acid Biosynthesis Protein)	BamHI	XbaI	ctagGGAtccCTTgagGAAatcGCAcagGAG	CCTCTCTAGACCCCTGGATGAGAGCATTT
lpg1979	ORF	BamHI	XbaI	ctagGGAtccCTGgagCAAgtcAATgctCCA	CCTCTCTAGAACCCGGCTCATCTTTTGAC
lpg1983	protein product; some similarities with 3-hydroxy-3-methylglutaryl coe	BamHI	XbaI	ctagGGAtccCAAacaGCCattGAAgcgT	CCTCTCTAGAGTGTCGTACCGCATCAACC
lpg1986	ORF	BamHI	XbaI	ctagGGAtccCTGagtGGCccaAGTcacCTT	CCTCTCTAGATCCCCTAAAATCAGATGAGGAA
lpg1990	ORF	BamHI	XbaI	ctagGGAtccCTAagtGCCaatCTGgtgTTTg	CCTCTCTAGACCCCTATTAAGTTGGAGCATGA
lpg1996	ORF (PilW?)	BamHI	XbaI	ctagGGAtccgccAGTcaaTTAttcGCTcc	CCTCTCTAGAGTGAACGAGCGAGTTGCAT
lpg2017	ORF	BamHI	XbaI	ctagGGAtccCCAttcCGTttaATGagcG	CCTCTCTAGAATCGAGCTCCCTGGTTGTT
lpg2019	serine metalloprotease (alkaline?) (acidic protease?)	BamHI	XbaI	ctagGGAtccggaCCAgaaGGAttgCGTt	CCTCTCTAGATTAATACCTGCGCGAATGC
lpg2044	conserved domain protein	BamHI	XbaI	ctagGGAtccCAAgtcCCAgtgACCaacGA	CCTCTCTAGAAACGATCCGGCTTTCATTC
lpg2048	ORF	BamHI	XbaI	ctagGGAtcccagGCGgaaACAgaaCTCa	CCTCTCTAGACAAAGCTCCAATCAGAAGGG
lpg2050	ORF	BamHI	XbaI	ctagGGAtccCTGcgcTTTactGAGcttGGA	CCTCTCTAGAGACGCTGCTGGCCTATGTA
lpg2053	ORF	BamHI	XbaI	ctagGGAtccGCAggaTTAtggTGGcaaA	CCTCTCTAGAACCTGAGCTAACGGCCCTA
lpg2060	ORF	BamHI	XbaI	ctagGGAtcccaaTCTtgcCCTgctGAAa	CCTCTCTAGAGGCCTAGGTATAGGGGCGT
lpg2073	ORF	BamHI	XbaI	ctagGGAtccCatGAGgtcAGAgatCGGgg	CCTCTCTAGAACTGAAACGGTCTGAGGGG
lpg2102	sterol desaturase	BamHI	XbaI	ctagGGAtccCccACCgtcCTTttcACCtt	CCTCTCTAGAGGCAGCCAAGTTTTTATTGC
lpg2112	24 kDa macrophage-induced major protein (alkylhydroperoxidase Ahp	BamHI	XbaI	ctagGGAtccCTtATCgctAACgcaCAAggc	CCTCTCTAGATCTTCCAGTTCAACCGCTG
lpg2115	hypothetical (phage AbiD protein)	BamHI	XbaI	ctagGGAtccCTgAAAggtGCCttgCCAttt	CCTCTCTAGATAATCCAGAGCCTCCTGCC
lpg2116	transposase, IS4 family TnpA (Tn5)	BamHI	XbaI	ctagGGAtccCGCtggGCTactTCTttgGG	CCTCTCTAGACACGGTAACCAAGCCATTG
lpg2128	ORF	BglII	XbaI	ctagAGAtctCTGcccCAGactTTGgatAG	CCTCTCTAGATACCAGGCTCAACTCGTGG
lpg2137	calmodulin-dependent protein kinase	BamHI	XbaI	ctagGGAtccCACcccAACccaGTAgaaGC	CCTCTCTAGAGAAAAACCCCAAGGTCGAG
lpg2143	ORF	BglII	XbaI	ctagAGAtctagGCactCACgatAAGcccA	CCTCTCTAGAACGGCATAAATGCCTCCTC
lpg2147	ORF	BamHI	XbaI	ctagGGAtcctgtTGGacaGCAaccCATt	CCTCTCTAGACCGGCGATTCCAGTTTTAT
lpg2148	ORF	BamHI	XbaI	ctagGGAtccCacGCAattGGAgacTCAgc	CCTCTCTAGAGCTTTTTCGCGACTCATCA
lpg2153	SdeC, SdeB, AliA, SdeA, SdeD homology	BamHI	SalI	ctagGGAtccGATatcCTAttgCGCgctG	CCTCGTCGACTTGCCTTCTCATCTGCCAC

Supplemental Table 1: Bank of C terminal fragments

ORF	Annotation	5'EndoR	3"EndoR	PRIMER_LEFT_SEQUENCE(5')	PRIMER_RIGHT_SEQUENCE(3')
lpg2154	SdeC, SdeB, AliA, SdeA, SdeD homology	BamHI	XbaI	ctagGGAtccCttGTGgctGGAcgaGAAag	CCTCTCTAGATGGGTGGTGTCAGTGGTTT
lpg2155	ORF	BamHI	XbaI	ctagGGAtccCaaTCGcgaGCActgACAct	CCTCTCTAGAAAGAGTCAGGGGCAGGGTA
lpg2156	SdeC, SdeB, SdeA, SidE, SdeD, AliA homology, C. elegans NMY-2 prote	BamHI	PstI	ctagGGAtccCTCaggGCTaacTTTaagGAAgga	CCTCCTGCAGTGAGCGGAAAAGAGGTTGA
lpg2157	SdeA, IMH1; Encodes a protein implicated in protein transport; induce	BamHI	XbaI	ctagGGAtccCcaACCatcCGTgtgTCCat	CCTCTCTAGACGGGTCAATAAGACCCGAC
lpg2160	ORF	NotI	SalI	ctgaGCGgccGCGcatAGCagcGGAccaTTA	CCTCGTCGACAAACCCGTTCAGCCAGTTC
lpg2162	ORF	BamHI	XbaI	ctagGGAtccCttTTGctgAGAgaaGCGga	CCTCTCTAGACGTTTGTGCCTTGCCTAAA
lpg2166	hypothetical protein	BamHI	XbaI	ctagGGAtccaaaAGTgaaCCAatcGGGc	CCTCTCTAGACCCAGGCTGATGACAACAC
lpg2170	ORF	BamHI	SalI	ctagGGAtccCTgGACgcaACGacaACAaac	CCTCGTCGACCATGCTCAGGAAATGGTTCA
lpg2176	sphingosine-1-phosphate lyase I (group II decarboxylase, pyridoxal de	BamHI	XbaI	ctagGGAtccAGTgagGATtcgCCTgctT	CCTCTCTAGAAGGAACGCGCGTAATTTTT
lpg2179	peptide synthetase (non-ribosomal) (microcystin synthetase) (similar	BglII	XbaI	ctagAGAtctCTgGATcctGGGcttGATagaA	CCTCTCTAGAGAGCAATGTCAGATCCGCT
lpg2186	polyketide synthase, type I	BamHI	XbaI	ctagGGAtccCTcGCAtgcCAAattGTTttc	CCTCTCTAGACAGCCAGGTGAGGTCAAGA
lpg2199	ORF	BamHI	XbaI	ctagGGAtccCtgGGTatgGCTtgcCTTtt	CCTCTCTAGATGGTGCAATCAGAATCGGT
lpg2205	ORF	BamHI	XbaI	ctagGGAtccacaCCAtggCTCtggAACa	CCTCTCTAGAAGACCACTTGCCTCGACCT
lpg2206	ORF	BamHI	XbaI	ctagGGAtccCTGgccAATgtcCCTtttGAG	CCTCTCTAGATTTATTCGGTGGCTAGGGC
lpg2210	hypothetical	BamHI	XbaI	ctagGGAtccCATtcaGAAattGCCctgCC	CCTCTCTAGAGCGATCCTGCAACAAACAC
lpg2212	acetylpolyamine aminohydolase	BamHI	XbaI	ctagGGAtccCAAatgGGAagcGGTtttTG	CCTCTCTAGACGGAAAACAAACTGGCCTT
lpg2217	chitinase domain (two component system sensor kinase) (sugar hydro	BamHI	XbaI	ctagGGAtccgggGTGaaaCTCaccGTTt	CCTCTCTAGATGGAGAAATACCCGCTAAATC
lpg2220	ORF	BamHI	XbaI	ctagGGAtccCccATTccaTCAgcgTTTtg	CCTCTCTAGATTTGTCATGCACAGAGCCA
lpg2223	ORF	BamHI	XbaI	ctagGGAtcccttGGTgggCTTtttGGTt	CCTCTCTAGATATGCAGTAAACGCGCACA
lpg2224	UVB-resistance protein UVR8	BglII	XbaI	ctagAGAtctCTGgctGCTtgtGGGctagAAT	CCTCTCTAGACAAATGTAAACAGGCCCTGAA
lpg2225	expressed protein (GH3 homolog) (auxin-regulated protein)	BglII	XbaI	ctagAGAtctCgtGAGtatCCCgctCAGca	CCTCTCTAGAGAAGCGCCTAAGCAAAACA
lpg2235	sterol desaturase	BamHI	XbaI	ctagGGAtccCTtCATggcAAAaacCCTcaa	CCTCTCTAGATCCTTAGCGGAATGGTTCA
lpg2239	ORF	BglII	XbaI	ctagAGAtctCTcCATgagAGGaaaCCCcat	CCTCTCTAGAGTTAACATCGCTCAACGCA
lpg2244	ORF	BamHI	XbaI	ctagGGAtccctaAACaagGAAgccCCCa	CCTCTCTAGAATTAGCCATCCAGGCTCCA
lpg2248	ORF	BamHI	XbaI	ctagGGAtccCCCgagCGAcacACTcttA	CCTCTCTAGATGGATAGATTCCCGCCTTC
lpg2257	O-sialoglycoprotein endopeptidase	BamHI	XbaI	ctagGGAtccAATgccGTTattTGCtacGC	CCTCTCTAGACCGTACCAGTCGCACCTAA
lpg2268	ORF	BamHI	XbaI	ctagGGAtccCTTtcgATCtcaAATccaCGG	CCTCTCTAGACTGAAGGATGGTTCCGCTT
lpg2271	ORF	BamHI	XbaI	ctagGGAtccCCTtacAACaatGCCcggCT	CCTCTCTAGAATCGGAGCGGCTGTTTAGA
lpg2275	hypothetical	BamHI	XbaI	ctagGGAtccCctCCTtttGTTtggGCTga	CCTCTCTAGACGCCATCGTATTTTCTGGA
lpg2298	inclusion membrane protein A	BamHI	XbaI	ctagGGAtccCTtAACattGACaggCTGggc	CCTCTCTAGAGAAGGCGGGAATCTATCCA
lpg2300	ankyrin repeat domain protein	BamHI	XbaI	ctagGGAtcccagTGGtggGCAaaaATTg	CCTCTCTAGATGTCTTACAGGCTGGACGC
lpg2311	interaptin	BamHI	XbaI	ctagGGAtccATAccgGAAgggCAGattG	CCTCTCTAGACCCATGCCTGAATCGAATA
lpg2322	cardiac ankyrin repeat protein	BamHI	XbaI	ctagGGAtccCTCggtCTTtttGAGgatGGG	CCTCTCTAGAGCTAAAGTGCCAGCCATGA
lpg2327	CG18304 gene product	BamHI	XbaI	ctagGGAtccCTCaagGGTggaAATgagCAA	CCTCTCTAGACTATGAAAATCAGGCGCGA
lpg2339	ORF	BamHI	XbaI	ctagGGAtcctcgGATcaaCACtggCTGt	CCTCTCTAGACACTATGGTTGCAAACGCC
lpg2342	ORF	BamHI	XbaI	ctagGGAtccCTtGTTtccAATgtaTTTgcgG	CCTCTCTAGACCGAATGCAAACAAACAGG
lpg2343	lysophospholipase A (glycerophospholipid-cholesterol acyltransferase)	BamHI	XbaI	ctagGGAtccCTAggtCGTtttTCCaatGGC	CCTCTCTAGACCGTTCTTATCATTGCGGA
lpg2344	ORF	BamHI	XbaI	ctagGGAtccAGCagaAATgtcGTCagcC	CCTCTCTAGATGGTCCATCTTGAACGGAA
lpg2351	ORF	BamHI	XbaI	ctagGGAtccCCCatgGATgctCTGgctAA	CCTCTCTAGAAATTTTTCAGGGCAGGGTG
lpg2371	ORF	BamHI	XbaI	ctagGGAtccCTTgccCAActaCCCaaaGAA	CCTCTCTAGAAGCTGAGTAATCCATTGGTCG
lpg2372	ORF	BamHI	XbaI	ctagGGAtccTACaacAGCacgAGGgagC	CCTCTCTAGATGACGTGCCTACCGATCAT
lpg2373	hypothetical protein	BamHI	XbaI	ctagGGAtccCTGgctGAAagtAAAaccGGC	CCTCTCTAGATGCCAAACCCATCCAGTAA
lpg2382	ORF	BamHI	XbaI	ctagGGAtccCTaGAAgccGTGtggGAAgac	CCTCTCTAGACCTCATGAGCGATTCCAAA
lpg2385	hypothetical protein	BglII	XbaI	ctagAGAtctCCTtttACCtggCTGcgtCA	CCTCTCTAGATGGTCGGAGCGTAACTGAG
lpg2391	sdbC	BamHI	XbaI	ctagGGAtccggaAATctgGGTgcgTTCt	CCTCTCTAGAAAACCTGCCGCGAAAATAC
lpg2392	leucine rich repeat protein family	BamHI	XbaI	ctagGGAtccggtGCAgaaTTGgcaCAAc	CCTCTCTAGAGAACGACGGTTGCTCAAGA
lpg2397	secreted peptidase (cell wall endopeptidase)	BamHI	XbaI	ctagGGAtccTTGgatGGAaagCATtggA	CCTCTCTAGAACGGCATAAATGCCTCCTC
lpg2399	unknown virulence protein	NotI	XbaI	ctgaGCGgccGCTaCGaaaGTGcaaGAAaccGA	CCTCTCTAGATGAGGCATGCCAAATTGAT
lpg2400	unnamed protein product	BamHI	XbaI	ctagGGAtccCTAgccATCcctTTCaccATC	CCTCTCTAGAAATACCCCGGTTTTTGCTG
lpg2401	putative secreted esterase	BamHI	XbaI	ctagGGAtccCTtGAAacaCTGgccCAAaaa	CCTCTCTAGAGCAATTTTCGCAGTAGCCA
lpg2404	ORF	BamHI	XbaI	ctagGGAtccCacCAGggaTTTgatCGAgg	CCTCTCTAGAGTTCCATGGGTGGTTTTCC
lpg2406	ORF	BamHI	XbaI	ctagGGAtccCAGccaGGGtttGCTtcaAT	CCTCTCTAGACCCGGCGTTCTCTTTTATC
lpg2408	hypothetical protein	BamHI	XbaI	ctagGGAtccCTtGGTtccAATaccGAGgaaG	CCTCTCTAGAGCTCGCACAAGGTGTGTTT
lpg2409	ORF	BglII	XbaI	ctagAGAtctCTGcaaACCgatGAGttgTGG	CCTCTCTAGAAGCTTCCGGCCTTATTCAA
lpg2411	ORF	BamHI	XbaI	ctagGGAtccCgcTACcaaTTGcagGCAga	CCTCTCTAGAAGGCGCTGCCCAATAATAA
lpg2416	ankyrin repeat containing protein?	BamHI	XbaI	ctagGGAtccCtcGTGaaaGTGatcCAGgc	CCTCTCTAGATCTTCTTGTAGCCCAGGATTTT
lpg2417	ORF	BamHI	XbaI	ctagGGAtccCTTtcgGTCcctTCTtatGGC	CCTCTCTAGAGGGATGGCTGCCACTATTT
lpg2419	ORF	BamHI	XbaI	ctagGGAtccCCGggaACAatgGGAgaaGT	CCTCTCTAGAACTCCTGCGACAACACCAC

Supplemental Table 1: Bank of C terminal fragments

ORF	Annotation	5'EndoR	3"EndoR	PRIMER_LEFT_SEQUENCE(5')	PRIMER_RIGHT_SEQUENCE(3')
lpg2422	hypothetical protein	BamHI	XbaI	ctagGGAtccCTActcCGGgaaCACcttGAG	CCTCTCTAGATTTGTCTGCAGCTTGGAGC
lpg2423	hypothetical protein-transmembrane region and signal peptide predicti	BamHI	XbaI	ctagGGAtccCTcCGCacgAAAcatTACgac	CCTCTCTAGAACATCGTCATCGCCACAAT
lpg2424	ORF	BamHI	XbaI	ctagGGAtccTTGcgtACAggaAGCttgG	CCTCTCTAGACCTCCTGGGTCACGCTATT
lpg2425	ORF	BamHI	XbaI	ctagGGAtccCccGGTcgaTCAgagCCTat	CCTCTCTAGAATTAAGGCGGTGTTTTGGC
lpg2433	ORF	BamHI	XbaI	ctagGGAtccGACctgGCActtGAAgcaA	CCTCTCTAGATTTCCAGCCAGCACAATAGA
lpg2435	ORF	BamHI	XbaI	ctagGGAtccCttAATaccGCCcccGATta	CCTCTCTAGATGCCATGCTAGAAAACCCA
lpg2444	ORF	BamHI	XbaI	ctagGGAtccCtgGGGttaTCAgctCAGca	CCTCTCTAGACGAGAAATCCGCTGGAAAT
lpg2452	hypothetical protein	BamHI	XbaI	ctagGGAtccCTGgcgCGAtgcTATgatAAA	CCTCTCTAGATCCATAGAACCCTTTGCCC
lpg2455	ORF	BamHI	XbaI	ctagGGAtccCTGgttGAAccaGATgccAAA	CCTCTCTAGACCTTGATCTTTGGGGGTGT
lpg2456	ORF	BglII	XbaI	ctagAGAtctgccAATgccTTCcagTTTt	CCTCTCTAGAACTCGATTATGGCGAGGCT
lpg2461	ORF	BamHI	XbaI	ctagGGAtccCTCcagGCTtttGATgaaAACaa	CCTCTCTAGAACTACGGCGCTCGAAAATC
lpg2464	ORF (sidM)	BamHI	XbaI	ctagGGAtccCTgGGAgcaGCTtcgGATtat	CCTCTCTAGAGCGATGCAGTATGGTGGAG
lpg2465	SidD	BamHI	XbaI	ctagGGAtccCctAAAgtcATGcccGGTga	CCTCTCTAGACGACGCTGTCCAAATCAGT
lpg2482	SdbC	BamHI	XbaI	ctagGGAtccCccCAAtctCCAtggCTAcc	CCTCTCTAGAGAGGCAACTGACTGGCAAA
lpg2490	LepB, non-muscle myosin heavy chain B (endosomal protein?) (interap	BamHI	XbaI	ctagGGAtccTCGctcAAAcaaAATgagGA	CCTCTCTAGATTCCCTTGGCACAATTTGA
lpg2498	ORF	BamHI	XbaI	ctagGGAtccCTtTTTgatGGCtggGGAagt	CCTCTCTAGATCCAAGCCAGGGATGTTTT
lpg2504	ORF	BamHI	XbaI	ctagGGAtccCGTcgaATGgatGACatgGG	CCTCTCTAGAGTGGCTTTGAGCATGGCTA
lpg2505	ORF	BglII	XbaI	ctagAGAtctctagGCCatgCTCaaaGCCac	CCTCTCTAGAGCAAAATACAGCGACCTGG
lpg2507	ORF	BamHI	XbaI	ctagGGAtccCTGcctGAAgagACGagcACA	CCTCTCTAGAGCAGGCGGTGATATTTCCT
lpg2508	ORF	BamHI	XbaI	ctagGGAtccCTgGTTtggGAGcatTTAgcc	CCTCTCTAGAGGTCAGTGCCCTGATGTTG
lpg2509	SdeD	BamHI	XbaI	ctagGGAtccCTCcgcAAGcatTTGactCAT	CCTCTCTAGAAGTAGGTGGCGTTGAGGTG
lpg2510	SdcA	BamHI	XbaI	ctagGGAtccCTtGAGgctCGTaagACCcaa	CCTCTCTAGATACGCCTCAACACAACCGT
lpg2511	SidC, interaptin	BamHI	XbaI	ctagGGAtccGACattTCTgccGAAtccA	CCTCTCTAGATGCCCAAGGGTGTCTGTAG
lpg2523	SidE paralog__E box at C terminal	BamHI	XbaI	ctagGGAtccgaaAAAggcAAAgacCCCa	CCTCTCTAGATCTGGATTGGGCATTCATT
lpg2524	transcriptional regulator, LuxR family	BamHI	XbaI	ctagGGAtccCTtGATcatGTCataGCGcct	CCTCTCTAGAGCTGTTTTTGAGTCAGTAGGGG
lpg2525	ORF/SidC fusion +	BamHI	XbaI	ctagGGAtccCTcATTgttGCAgaaGCTgatGA	CCTCTCTAGAGGCATTTTTAAATTGGCAGG
lpg2526	ORF/SidC fusion +; moderate defect in yeast	BamHI	XbaI	ctagGGAtccCTTcccGGTataGGAacaGGG	CCTCTCTAGAGCCAATTTAAAAATGCCTCG
lpg2527	contains coiled coil domain_E box at C terminal	BamHI	XbaI	ctagGGAtccCTGgatGTTttgACAgagCCG	CCTCTCTAGAGCGCTGGTTATGCAAGAGA
lpg2529	ORF/SidC fusion +; moderate defect in yeast	BamHI	XbaI	ctagGGAtccagcCCAactGCTgtgACAa	CCTCTCTAGATGCGGTTGCTATTTTGCTT
lpg2535	limited homology to myoglobin (hemoglobin, cyanoglobin)	BamHI	XbaI	ctagGGAtccttgTTTgagCGAttaGGCg	CCTCTCTAGATCGGGTAATCCTTCTTGCC
lpg2538	ORF	BamHI	XbaI	ctagGGAtccCTtGGTcaaCGTaccAGTggg	CCTCTCTAGATCAAACCAAACGAAGCAAAA
lpg2541	ORF	BamHI	XbaI	ctagGGAtccCcaAGCgagGATtgtCTGga	CCTCTCTAGACAGCAACTGCCAAATCCAT
lpg2543	ORF	BamHI	XbaI	ctagGGAtccCTGgcgAAAgagCAAtgtTT	CCTCTCTAGATGATGTTCTGCCAAAGGGA
lpg2546	hypothetical protein	BamHI	XbaI	ctagGGAtccTCAgggAATtgtTGCcaaG	CCTCTCTAGACAAATCCGGCTGGAATAGAA
lpg2552	ORF	BamHI	XbaI	ctagGGAtccCTtTTCaatGCCttaGGTgcg	CCTCTCTAGATCATTGCTGGAACCGTGAT
lpg2555	ORF	BamHI	XbaI	ctagGGAtccggtGCAgtaGAAgcgCTGa	CCTCGTCGACTAAATCCCGAGGACAAGCC
lpg2556	putative protein kinase	BamHI	XbaI	ctagGGAtccgcaGCGcctGAAatgTTTa	CCTCTCTAGATTTGTTAGCGATGCGTTCC
lpg2567	phage repressor	BamHI	XbaI	ctagGGAtccCGGgtTGGactCAAgaaC	CCTCTCTAGAGATTTAACGCCGTGGGAAT
lpg2572	ORF	BamHI	SalI	ctagGGAtccCTcTCAcgtGCTttcGCAaat	CCTCGTCGACTGCTTCCACTTCTTGTTTGCT
lpg2577	ORF	BamHI	XbaI	ctagGGAtccCaaACGcaaAAAgatGAAcgtG	CCTCTCTAGAAAGGCTTCGAGACGATGCT
lpg2584	SidF, inhibitor of growth family, member 3	BamHI	XbaI	ctagGGAtccgccCTTcatCATactGCCa	CCTCTCTAGACCTTTGGGAAGCGAAAAAC
lpg2586	cysteine protease (papain C1 family)	BamHI	XbaI	ctagGGAtccCTTggtTGGatGGCtcaTTA	CCTCTCTAGAATGAACCAGGATGAGGGGA
lpg2588	acid sphingomyelinase-like phosphodiesterase	BamHI	XbaI	ctagGGAtcctcgGTTcaaTGGaccAAGa	CCTCTCTAGACGACAAGAAGCGAGGATGA
lpg2603	ORF	BamHI	XbaI	ctagGGAtccCttGATggtGGCtttCGGta	CCTCTCTAGAGGCTCCATTTTTATGGGGA
lpg2622	hypothetical protein	BamHI	XbaI	ctagGGAtccCTAgccATAgatGCTgccCTT	CCTCTCTAGACAACGACCCGAGGTTTCTT
lpg2635	integral membrane protein (putative virulence factor) MviN, role in mo	BamHI	XbaI	ctagGGAtccCTtGCGacaGTTatcTTGccc	CCTCTCTAGAATTTGCCCTTAAACTGGCG
lpg2637	ORF	BamHI	XbaI	ctagGGAtccTTGgcaTCGgatTTTtgtC	CCTCTCTAGAATCGAGAGTTTCCGTCGCT
lpg2638	ORF	BglII	XbaI	ctagAGAtctCGTgaaCATcccACAgtgCC	CCTCTCTAGAACCAACCCAAGCTGGAATC
lpg2643	ORF	BamHI	XbaI	ctagGGAtccagtAATtcgAGCcttGCGa	CCTCTCTAGAaggcgtttttcggtggtta
lpg2693	hypothetical (SmtA SAM-dependent methyltransferase?)	BamHI	XbaI	ctagGGAtccTGGtctGCAaccAATtatGC	CCTCTCTAGAGCGTTGGAGTCTGCTTGAA
lpg2694	hypothetical (SnoK?) (eukaryotic proteins (PhyH (bacterial dehydroger	NotI	XbaI	ctgaGCGgccGCTtttTCCacgCAAaccAAC	CCTCTCTAGATCCATTCGGGATTTCTTCC
lpg2718	ORF	BamHI	XbaI	ctagGGAtccCTAatgGCAgcgTCTcagTTG	CCTCTCTAGAGAAGCATTTTGTGCGCATT
lpg2720	hypothetical protein	BamHI	XbaI	ctagGGAtccCcaGCGgttGCTaagGACaa	CCTCTCTAGAAGCAGCCATACCAAAACCC
lpg2733	hypothetical (transmembrane protein)	BamHI	XbaI	ctagGGAtccCTTaacCAAcccCCAgtgGAT	CCTCTCTAGAGGGCGAATACGCTTTGTTT
lpg2744	ORF	BamHI	XbaI	ctagGGAtccCacATGggtAGTgcgCCTtt	CCTCTCTAGATCCCAATGTAAATTCCGCA
lpg2745	ORF	BamHI	XbaI	ctagGGAtccTATcagCTGgtaGAGgcgG	CCTCTCTAGAAGGCCAAGACAATCGATCC
lpg2759	ORF	BamHI	XbaI	ctagGGAtccCcaGTAcgtATGcgcCTTgg	CCTCTCTAGAGAAACGTTTGCAATGCAGC
lpg2761	ORF	BamHI	XbaI	ctagGGAtccCTtTTGcttCGAtctGTTgaaAAA	CCTCTCTAGAGCGCTCAAAACGAAAACAG

Supplemental Table 1: Bank of C terminal fragments

ORF	Annotation	5'EndoR	3"EndoR	PRIMER_LEFT_SEQUENCE(5')	PRIMER_RIGHT_SEQUENCE(3')
lpg2762	ORF	BamHI	XbaI	ctagGGAtccCTgCATcagGCTggtGATttg	CCTCTCTAGATCGCTAACTGGCCTCCTTT
lpg2793	LepA, interaptin	BamHI	XbaI	ctagGGAtccCTtCAAcacCAGgaaTCTgcc	CCTCTCTAGAACCTTCTGGCACTGAGCCT
lpg2800	FrgA (siderophore biosynthetic protein, iron repressed gene)	BamHI	XbaI	ctagGGAtccgcaGTTccaTTAcctGCCc	CCTCTCTAGAAGATTGGAACAAGAGCGCA
lpg2803	ORF	BglII	XbaI	ctagAGAtctcagCCTgatTTGtatGGCg	CCTCTCTAGACTTCGGATTTGCTCTCGAA
lpg2804	ORF	BglII	XbaI	ctagAGAtctcttGCCtctagTGtcaGCGg	CCTCTCTAGACAATTCCCCACTGGTTCCT
lpg2806	ORF	BglII	XbaI	ctagAGAtctTCGgttGTCcagGAGgacT	CCTCTCTAGATAGAACCCAAATCGCTCCC
lpg2813	ORF	BglII	SalI	ctaAGAtctCTcATCggcCCGactTTAgat	CCTCGTCGACTTCGCTGATCACAACCCAT
lpg2815	ORF	BamHI	XbaI	ctagGGAtccTTGtttCTGgggCATttgA	CCTCTCTAGAATCCCACCTGGAAGGACTG
lpg2828	ORF	BamHI	XbaI	ctagGGAtccCTCgaaGCGagaAGAccaCAG	CCTCTCTAGATCGCTACGCCTATGGAGACT
lpg2829	SidH (myosin-like protein)	BamHI	XbaI	ctagGGAtccCcaGGGcttGAAaccCAGtt	CCTCTCTAGATTTCGAAAATCTGGCCTTG
lpg2830	ORF U-box domain, putative role in ubiquitination	BamHI	XbaI	ctagGGAtccCTGgcgACAcgaAATcctTTT	CCTCTCTAGAGCCACATCAACCAGCAAAT
lpg2831	unknown	BamHI	XbaI	ctagGGAtccGGGgaaGTCacaACCacaA	CCTCTCTAGACACACTTGAGCCAGGAATGA
lpg2837	lysophospholipase A (GDSL-motif lipase/hydrolase?)	BamHI	XbaI	ctagGGAtccATAcggCATccaTTGggaA	CCTCTCTAGATGCTTAAAAACCGCTCTGG
lpg2851	protoporphyrinogen oxidase	BamHI	XbaI	ctagGGAtccCTCgcgGAAcaaTTTgatGT	CCTCTCTAGAGAAATTCACGACCAAGGGAA
lpg2862	ORF	BamHI	XbaI	ctagGGAtccTCGctgATTggcAACtacC	CCTCTCTAGACCCATATTAGTTGCCGCCT
lpg2874	ORF	BamHI	XbaI	ctagGGAtccCTGccgAGAgatAAAgggGAA	CCTCTCTAGAAGGGATCTCCTTGCTCCAA
lpg2877	hypothetical	BamHI	XbaI	ctagGGAtcctctGCTccaCATgctCACa	CCTCTCTAGAGGGGGCAGCAAGTAACATT
lpg2879	ORF	BamHI	XbaI	ctagGGAtccCTTcgcTTTgtaGCGgatGTC	CCTCTCTAGAAAAGCGCCCCATTTACAAC
lpg2884	ORF	BamHI	XbaI	ctagGGAtccaaaCAAtttGCAactGGCg	CCTCTCTAGAAGCCGCGTTTTTAATGACC
lpg2888	ORF	BamHI	XbaI	ctagGGAtccCtcGCTtttGCAttaCTGgc	CCTCTCTAGATCTGTACCAGCATGCCCTC
lpg2900	CapM protein, capsular polysaccharide biosynthesis (glycosyltransferas	BamHI	XbaI	ctagGGAtccCTtCAAccgGATtgcATCatt	CCTCTCTAGACGCACCTTGCCTGATAAGA
lpg2907	ORF	BamHI	XbaI	ctagGGAtccCTGcttGACagtTTGggaGCA	CCTCTCTAGATCAGTCGATCGTTTTGGCT
lpg2911	serine carboxypeptidase (protective protein for beta-galactosidase)	BamHI	XbaI	ctagGGAtccCacGGGctgATTgacAACaa	CCTCTCTAGAGCAGCAAGCTTTCTCCAAA
lpg2912	ORF	BamHI	XbaI	ctagGGAtccGAGgcaTTGcgaCAAcatT	CCTCTCTAGAAGGCAGACATTTGGGGTTT
lpg2938	ORF	BamHI	XbaI	ctagGGAtccGCAactTCAttaAGAaacGCTc	CCTCTCTAGAGCCGTGAAACTGGGAATTT
lpg2939	hypothetical (BNR/Asp box repeat protein)	BglII	XbaI	ctagAGAtctCTGcgcAAGaatAGCcatCAA	CCTCTCTAGACAATGCTAGGCATTCCCAA
lpg2942	ORF	BamHI	XbaI	ctagGGAtccgcaCAGggtGATctcATGg	CCTCTCTAGATGCATGGTTCACCTCCACT
lpg2952	ORF	BamHI	XbaI	ctagGGAtccCGGgcaCGGaagTCAgaaAT	CCTCTCTAGATGATCGTTGACAATCCGCT
lpg2953	ORF	BamHI	XbaI	ctagGGAtccgaaGATgacTTGgctCGCa	CCTCTCTAGATGCGCAACTCTTTTCTTGC
lpg2954	Hypothetical protein	BamHI	XbaI	ctagGGAtccAAGgcaAGAaaaGAGttgCG	CCTCTCTAGACATCAGGCGCTGACAATTT
lpg2975	ORF	BamHI	XbaI	ctagGGAtccCcaGGGgagGGTgatACAgg	CCTCTCTAGACGTCAGCAAAATACCCTGC
lpg2976	ORF	BamHI	XbaI	ctagGGAtccTTGcttCGCttgTTTttgC	CCTCTCTAGATGCCCAAAGCAATAAGGGT
lpg2977	zinc metalloprotease (virulence metalloprotease, hemagglutinin/protea	BamHI	XbaI	ctagGGAtccCatGCTgatGGCagaACAgg	CCTCTCTAGAGCAACGCTGCAATAAAACG
lpg2995	lipoprotein (exported protein)	BamHI	XbaI	ctagGGAtccCTTggtCAAtggAAGcaaGGA	CCTCTCTAGAAACATATTCGGCACCGTCA
lpg2999	CG6763 gene product (eukaryotic homologs?)	BglII	XbaI	ctagAGAtctTCAtggATTgccAGTttgTT	CCTCTCTAGATATGGCCTTAGCCACTCCC
lpg3000	ORF	BglII	XbaI	ctagAGAtctCTCgacGTAtgcATGgctTT	CCTCTCTAGATCCAAGAATGGTCAGCCAA

Supplemental Table 2: Pool for BLOCK searches

Lpg number	Annotation	C terminal sequence
>lpg0008	ORF	NSQMEPIVSDSEEEEEKQNCWTSFCGLFGK
>lpg0012	ORF	DGLKKHSILAAKQTHSAIQEEEEVRLNLGV
>lpg0016	ORF	DQELPAFKETHELEILYQLILSWNYLCADG
>lpg0030	ORF	SHPASRYKQEDVVNQLDQWIAQNSTVNFLK
>lpg0038	"legA10"	FRPKLINWLEKQNDLEPQVTYRFSGQSSSA
>lpg0059	Orf	KLFQPVNKTALRPNDEETDELSCKPVLLQR
>lpg0086	ORF	PDSPSIPHSKSESALNQLIPSQNSTILGLA
>lpg0096	ceg4	PSILSRRTPTKGKEEQCQEEYVLTNPLHNS
>lpg0103	VipF	KKALNLYIQLGFHIQNACDYWSINVNQLAK
>lpg0107	Orf	LESRHGHHLEIIIIVLIAVEIIIAVMNFHF
>lpg0126	lpg0126	GRWRLFPVPTESEKIVKDFSLSMQPLSVSA
>lpg0135	sdhB	KEELHTIKNTDGASPRKGEGQEGEIEFTKL
>lpg0160	ORF	LFSFWCEKNESFVESRKIIEEHRICRLNNL
>lpg0171	"legU1"	FAQPSQSAAQQNLEEENLSADPKACQCEPL
>lpg0172	ORF	NIVIIKKLTQSISQGPAVLDEAVDNIPTLS
>lpg0191	ceg5	IISLSTDNEVLAAEDAISLVQPNLGKPQLK
>lpg0195	ORF	AKIKTKDQVIIRGINEPEVAEQISKLSKKM
>lpg0196	ORF	KFQDTVKEKSEKAVREQDESEENRIGLTIS
>lpg0197	Orf	ANGIIAMRYDTGEIGQAGTEVLCYGTAVII
>lpg0210	ORF	LQDSLIVNNSDIYNVAEDNTMDWISNCPQP
>lpg0227	ceg7	HVFGKAQTGLESDEALAAQLQEAELRRILY
>lpg0234	sidE	DELLNEFGKQAPRTEMIKTVEEKQGTLLRL
>lpg0246	ceg9	YKYDILDLIKRVHKDKFEEMEDENLIRMSI
>lpg0275	SdbA	IPEVREPFCSKEDLHHNELSSQSLVSVSAG
>lpg0276	legG2	REPMDIEVMDMDVMDYDENDLRDQTVMITV
>lpg0294	ceg10_Translocated	QEPKALETKREEIRQEIESGAEAPTTQSIR
>lpg0376	sdhA	AISSESRAITPVSSVENNGTIELNQLAPSA
>lpg0390	VipA	FLKELNAKIKERKERPKDEPHTTPVEKKNL
>lpg0401	ceg11	FLTSTKTTTKPLSRSHDDEKKTGMDGHGFH
>lpg0402	"legA9"	AKPCDGSESWVPEYIKSCYEKSGHKSSSLF
>lpg0403	"legA7"	FKFSETKGTLFQRGVKTISYQSQQPRFGMK
>lpg0422	"legY"	LTWSYVSVLRAIHLREQLENRIKTTGWNTY
>lpg0436	"legA11"	FMTGGEIPPSRKVPESRFEEAGIDTPKNAL
>lpg0439	ceg15	FFHKMKSASDGITTLEKTLALEDDKINPFA
>lpg0483	"legA12"	RIVLNEMFNPIPRVHEDIESQQSSPRVVLK

Supplemental Table 2: Pool for BLOCK searches

Lpg number	Annotation	C terminal sequence
>lpg0515	"legD2"	KALSFQFIETYEVDYPETNWLYPPNKVYLY
>lpg0518	near "legD2"	TRLGTTWVSACLKPEVTETEHENTTAYQFN
>lpg0581	hypothetical	LQEIKKHQRIFKMRDDKDYKIDQSEIWEEF
>lpg0621	SidA	EHTKIAEQKTTDTTNEQVPTTFDSISKPTR
>lpg0634	ORF	FKKAPSMSEEDEEDDLKDQNTAEETSKPTV
>lpg0642	WipB	GGSLDKRIKFIELQIIAQRCDSPEPQFNLK
>lpg0693	ligA	ILSYGEEAFSNPGEELQTDSVMQPPMVFNI
>lpg0695	LegA8	KLLIKNQDISRKVDKKSIQEAVGTSLKLKW
>lpg0696	ORF	ENQNKLYKAFHKKIELQLLDSEIENKNELK
>lpg0898	ceg18	ASQTKSTEQKTLEIEHLPTEEIQPPSLAIH
>lpg0926	ORF	DVSDLPMVKSSETTDTQPNQSFSMSNGHGD
>lpg0940	LidA	PSVTSIRSKTDEVLEQRFNPTPSLSPFKTS
>lpg0941	ORF	PKSQQLHFPGLFHGTTQTPDTMPQEHTLTI
>lpg0944	ORF	GTLKEQIGFIKIQEQNPVEEIENLSRPNLT
>lpg0945	Gala protein	KFLQKFTFLSCMSPCQETAESDKGLNKKPN
>lpg0952	VipD paralog	DFHKAKEAIHEGENCVLREKNNILQLVGLE
>lpg0968	ORF	QVSDEITKIKLKLEDTLGSLQKVQEESLSL
>lpg0969	ORF	VTITPEVKILFYQLVKEHFHSPETEIKLDI
>lpg1098	VrrB	NVLQADGQSASHEHQKHKGHGGYGGGVGGR
>lpg1101	sdmA	TGIKTDSQRAVEEIFSKALKDLQSPKLAMR
>lpg1106	ORF	SQLLINLINAIDLGLIDMELGSLSITSAKL
>lpg1108	Putative lipase	EEVKISKLGELTLIEDYYPVKSEPDSLFNP
>lpg1109	ORF	YRRDRDLNRRAISHLLTEPLENSYKASFSL
>lpg1111	ORF/paralog LidA	LFFNILLVPENQGHEPEQENTGEHTHGHSH
>lpg1120	hypothetical	LFSKPTNINPNQELDIDLNQKLNLETKTKN
>lpg1121	ORF	TTMKPLLEDEHIGNELKDLLSIKTDNVNII
>lpg1129	ORF	FFSDSPKNPDEHKTEKDKTISPSRNPTKGG
>lpg1145	ORF	ILKDLTEVSYVLNKLKGQTPANNSDPDFGL
>lpg1148	ORF	HSPTLFFTLATQERNTNDMADELSSSGPKT
>lpg1152	ORF	YQLVRNRFFSSKPEKEELEQENILQKTIQQ
>lpg1154	ORF	KGKIKLYLAKIMDIQNEILTSKNGEFVPGL
>lpg1158	Orf	EEVAKKIPGDMKDEFTDIEPSSTKPSSTGS
>lpg1166	hypothetical	YDVEALQDTYQELEDLYEEYCNPKKELLNF
>lpg1183	ORF	FSIWAAKQENLDFGIEEVESVIKQRYVMKS
>lpg1227	vpdB	LLKGLPGLNNTSSHCTEEFNEQSTPSLYRA

Supplemental Table 2: Pool for BLOCK searches

Lpg number	Annotation	C terminal sequence
>lpg1290	Myo like	KTEKTEKTEKTEKTEKTENEQSRNTRGFPI
>lpg1312	legC1	QIEKVPEKNVNQNNKQKSLEEGEPPCCTII
>lpg1316	ORF	SFFKEMQRNAKNQLESSKPQDDTPVLRKSY
>lpg1317	hypothetical	GKNNCNAYRQYFKEIQKEAADTVSKFDLKK
>lpg1328	"legT"	SGQPNSTSYTIRFCPGGITGLPAGVNEGRG
>lpg1355	sidG	EPSMSSTIDEEENIDSEHQIETGTESTMRI
>lpg1426	vpdC	MEPVQTVKGGVHHDDLNNGSHERYRFGLGV
>lpg1483	"legK1"	TGRLGLFSQTRSEKLVEEMKSSVLGIAVGN
>lpg1488	legC5	GFFALKSSLAVSQSQNLEGAINSELSQKNC
>lpg1489	ORF	NKELSGIPRLVNKAPEHEILPSDDKTSLHH
>lpg1525	CAAX protease	WLFHRQKSLLGVSVSHVILGVWSLFIVGLR
>lpg1551	ORF	FDKLKKDDQHGLNHFLTEYDPDLQFRPSRP
>lpg1588	"legC6"	FFATTRAKSAEAVEKAESELELNQLGSLPK
>lpg1602	"legL2"	VASNNLRFAREVQKLLPDEVTERIVKNILG
>lpg1621	ceg23_translocated	GYGLYSFFSSSPKVSERDSTNGLSQKLKIN
>lpg1642	SidB, RTX	RSGKSAQTFFREFVQRTEADHAVKSIPEIN
>lpg1660	legL3	APLFFISPKSRQEENKNELSSLPSHSVHLH
>lpg1665	hypothetical	VPPAYAEAMYNPNTKARGSSSVITVSREED
>lpg1683	ORF	QKQIKTDLLDLREEDKTGLKKPLHGGIKVK
>lpg1687	ORF	KLLPHFWNRHGKSSSSYWEEVFTPSCLNNK
>lpg1692	SidC, SdcA paralog	AVWRLVEDAKANIIEPEQEQVPDLRTSGPS
>lpg1701	"legC3"	STHSEESDEGLNDSNDSLQALEEETVNEIA
>lpg1718	"legAS4"	LRESGLHLPEDLLEQLGIMDSSITLESKFF
>lpg1752	ORF	ALRHSREKQETSFYDIYKGIMRTLGLNSVF
>lpg1797	ORF	KSRLQAVVSERNAHKDDTEEVTINNHNSLR
>lpg1851	ORF	KPKSSQGGLFSVPSGKEKVTDTHEVANHRL
>lpg1884	legC2/ylfB	KLGIMATEEKESPTPVSAEENTIPTQTLTG
>lpg1890	legLC8	AIPVMFQDFAKPGKNETDDNTINSITAIPK
>lpg1948	legLC4	EKKESFFYYEGGRRDVMSLISAKMPSIQII
>lpg1949	hypothetical	LAVKGEMESAREIYTTVDREKSNLQSHKIN
>lpg1950	RalF	TIERNLALKEGVPKDPDAEMQKEKGRQLKF
>lpg1953	"legC4"	LEGSFGMLAGVLNQFLEGSTSESESFDIKL
>lpg1958	legL5	AKNKEHRSIQDYPSLLPKELTDSISSLLKC
>lpg1963	hypothetical	TDEKNIFKLSDEKHLSENKEDIRGKGITNI
>lpg1966	ORF	NAFFKLSKETNKENVIDQVNQTDVFGFCRN

Supplemental Table 2: Pool for BLOCK searches

Lpg number	Annotation	C terminal sequence
>lpg1976	"LegG1"	NPAMFFPPRQDKSAINNLMKISKFSPCNLL
>lpg1978	glucosyl transferase	ETGQFYKKVAAIDLQTTIAAEYDNNHGLRI
>lpg2131	"legA6"	SPSLISAIHWKKNLLETRCCFSNEFISAVS
>lpg2137	"legK2"	DTKRKLQEMKSSANQDKQPIFTPSLMRGPS
>lpg2144	"legAU13", ceg27	AFKDKEKKQIRALIRGMQEEKIAQSKCLVC
>lpg2147	paralog of 2148	PSEQLAEIKAESETAQKAWDEQYGLVLRDK
>lpg2148	ORF	PESSPSLFWKNVDKKEQEMTDSSRYKKGPS
>lpg2153	sdeC	VSTKIGKEPVKQDHTITIDEEESDDIRYGF
>lpg2154	SdeC paralog	SLLRHVFWCCPSNQKRDDENTDVLEKKAQL
>lpg2155	sidJ	FWREARKNLSEKSDIDSEKPESERTTDKRL
>lpg2156	sdeB	KIGTEPVKLESTHTVDEDERDTYHRESGYF
>lpg2157	SdeA, IMH1	DISTKIGREPEHLKFVMVEEDESNKKTIGF
>lpg2176	"legS2"	ASPGSLRIFTNVPVEEDEGLRNRKTEKYKV
>lpg2199	hypothetical	LRLCQYNPKIYLDVATRLEEPAEQSNTLDF
>lpg2200	hypothetica	YQGGDFKASKEDFTSFKMPEREEPKMGMQL
>lpg2206	WipC	SMPNVFDLDNLFGKGEDFYQGPYAVHITHS
>lpg2210	hypothetical	KWWYVPNPDYSDRMLNEEDKKLLSMINQAK
>lpg2215	"legA2"	RAEESGVKKSFFAFPKKLVGVEQLQIPKLY
>lpg2248	ORF	LQYLSRHFTSSLNKLASVLEVQLFANRYVP
>lpg2257	ORF	YAIKKSDMTKENRDKWIKNLKNSFIKISTP
>lpg2271	ORF	KTSNSTSSVRNKEAEVEESSQRLYPNLQTV
>lpg2298	"legC7/ylfA"	SHGIYSTPKGKVTQPKVEVVEDRQTIALVN
>lpg2300	"legA3"	LTRKGNNLLKILDDCGINPNIGVNPVATGL
>lpg2311	ceg28	KELNPVHKTTERTIEQEGPAPESPSTGMRK
>lpg2322	"legA5"	DLKRLSLFDSRDSSTLDNVFLDISLSKNKI
>lpg2327	CG18304 gene product	FFAYARPDSAKAVEKAEEDLGLRQAASSPK
>lpg2344	ORF	FENPYVLFPFVAATVAVAATAASVLFGNKP
>lpg2344	ORF	FENPYVLFPFVAATVAVAATAASVLFGNKP
>lpg2351	ORF	MYLSEEYKNRHQHPNKDPDLKTNESLKPGL
>lpg2391	sdbC	PSIAGFFSRSTPVIEEVETEKQEQKSIELK
>lpg2392	"legL6"	ILFTQRHQKDSDNKIIPKELEESIRTFNSR
>lpg2400	legL7	LIFAQKHQTNIEDLNIPDELRESIQTCKPL
>lpg2409	ceg29_translocated	ISSLVHLGLFRDTQIQYEEKEESLHNKYRK
>lpg2410	vpdA	RILEFIKSDDISGLMTILEDKKALPNNKPN
>lpg2416	"legA1"	LSIPSVLALTKANNYYINETGGRLDLRAVA

Supplemental Table 2: Pool for BLOCK searches

Lpg number	Annotation	C terminal sequence
>lpg2423	hypothetical	IMASWVTLLAIHDEKALDDLEGLLKCQNAS
>lpg2424	ORF	RLKHRHRHNLFKPQIEEQKINEQNQSLFLG
>lpg2425	ORF	LNSWVHIKNQLSQLAGKEVSQERQSLVAPM
>lpg2433	ORF	LGNGLSIYTPEKPSVKTLPPLKGVRLTSLP
>lpg2444	ORF	ALKWGLFGGAALAITYAVSKLSDKSHTNSL
>lpg2456	"legA15"	ADPKLKQTLETALKQASESSMTLSQPGKTI
>lpg2461	ORF/rhoptry protein	HETVTELYPEFTDPENPMSGYIYSTFDSPS
>lpg2464	sidM	KTSSVSSFEKMVEETRESIKSQERQTIKIK
>lpg2465	sidD	HNPAGSDIHSTLNLWTADKIKAATNSSLTI
>lpg2482	sdbB	FNTVVDLITEEQQLRHDRFNDNTIGKSISM
>lpg2490	LepB	TFFSQTNSGKKLDALEKAISSTHSETLVYG
>lpg2498	ORF	ILWFAPQDKKTIPKPSSQEELKQNQLSFLN
>lpg2504	ceg32	CPELGFFGSRRGDLEAKQENNKLKESILVF
>lpg2505	ORF	AVKTFENRFKDSMSTAGKDDERSITRPIIL
>lpg2507	ORF	ESPQRLIESFHFIQTHQLGMWEQFSNALFN
>lpg2508	sdjA	RFWREHKKENQSLEKGFDFDRNTSSSQSPL
>lpg2509	SdeD	FVEKLLKPFMIWKKPEVQTTQPTTEKTNKP
>lpg2510	sdcA	ITEMARKYRETINEMTGRNESLRETVRNTI
>lpg2511	SidC	TSKQFREAMGEITGRNEPPTDTLYTGIIKK
>lpg2525	Orf	SQNLYINQDETIELFNKYTEEKTNNFCSIL
>lpg2526	ORF	RSMFSLFSHSTTTEPSPTVKEEESKKSGPQ
>lpg2529	ORF	DDVILFVQEMDKIIKQQESISENSSKRAAM
>lpg2556	"legK3"	HASEANIIRKINGFLEDIEYQNLTNSGFEL
>lpg2577	ORF	MEKDLVGEEYKSSLPNPFDISRGPKLPDWI
>lpg2584	SidF	KPKDMTSKVDLTAAMEDDNRSESTPTPVNF
>lpg2588	"legS1"	VCNNLEVGLFYGVKSTRVPYQGCIKTYQIN
>lpg2603	sdmB	LNLKTSSRKKVMVIFKEAEERILNSPNVSI
>lpg2694	"legD1"	RHAYTLHLVDISLPYPEKNWLQWPNGIPCL
>lpg2718	WipA	LSLLGKPKAVSGSLSVDQNISIPNQASLGK
>lpg2720	"legN"	LFNLICGSIISLARSLKKLDVRLNIEKYNQ
>lpg2793	hypothetical	SKRPCFFKTRSHRLLEEVDTILHSMAPTSS
>lpg2813	VipE	TKGTFKFWKSHGEALSDNIEEVSKQQPGMK
>lpg2815	ORF	HRLFNQKGEKTRTQLFIEELPERINLSSGK
>lpg2829	SidH	QNIKGPEPVATPMETPENEAPLVNANITRF
>lpg2830	"legU2"	GFGFFSLNFLTSWLWGTEEKKEKTSSDMTY

Supplemental Table 2: Pool for BLOCK searches

Lpg number	Annotation	C terminal sequence
>lpg2831	VipD	IAEVRREHTDPSPSLQEKERVGLSTTFGGH
>lpg2832	hypothetical	DIPVMKEICYQTVVNSIETMQERRTHLSQE
>lpg2862	"legC8"	ITSSEEVERTQSLRTDGLSWMPSEQARLSK
>lpg2879	hypothetical	ISPDSLFSTRRRRVNPDQEKVAITELSMTK
>lpg2884	ORF	HTTKVFAQPKDVTPEVVENPTSTVSLTGSK
>lpg2975	ORF	DYFKEVKNYAEPISHRQETTLNSNVTSLVV
>lpg2999	"legP"	IPLVDGVEIGQRTHLSEKDIAAVKAMYPET

Supplemental Table 3: Location of E Blocks

GENE	C terminal Sequence (E Block)
>lpg0008	NSQMEPIVSDS EEEEEK QNCWTSFCGLFGK
>lpg0012	DGLKKHSILAAKQTHSAIQ EEEEVR LNLGV
>lpg0016	DQELPAFK ETHELE ILYQLILSWNYLCADG
>lpg0059	KLFQPVNKTALRPND EETDEL SCKPVLLQR
>lpg0096	PSILSRRTPTKGK EEQCQEE YVLTNPLHNS
>lpg0126	GRWRLFPVPT ESEKIV KDFSLSMQPLSVSA
>lpg0135	KEELHTIKNTDGASPRKG EGQEGEIE FTKL
>lpg0160	LFSFWCEKNESFVESRKII EEHRIC RLNNL
>lpg0171	FAQPSQSAAQQNL EEENLS ADPKACQCEPL
>lpg0195	AKIKTKDQVIIRGIN EPEVAE QISKLSKKM
>lpg0196	KFQDTVKEKSEKAVR EQDESEEN RIGLTIS
>lpg0227	HVFGKAQTGLESDEALAAQLQ EAELRR ILY
>lpg0234	DELLNEFGKQAPRTEMIKTV EEKQGT LLRL
>lpg0246	YKYDILDLIKRVHKDKF EEMEDE NLIRMSI
>lpg0294	QEPKALETKR EEIRQEIE SGAEAPTTQSIR
>lpg0390	FLKELNAKIK ERKERP KDEPHTTPVEKKNL
>lpg0422	LTWSYVSVLRAIHLR EQLENRI KTTGWNTY
>lpg0436	FMTGGEIPPSRKVPESRF EEAGID TPKNAL
>lpg0483	RIVLNEMFNPIPRVH EDIESQ QSSPRVVLK
>lpg0518	TRLGTTWVSACLKP EVTETEHE NTTAYQFN
>lpg0634	FKKAPSMS EEDEEDD LKDQNTAEETSKPTV
>lpg0693	ILSYG EEAFSNPGEE LQTDSVMQPPMVFNI
>lpg0898	ASQTKSTEQKTL EIEHLPTEE IQPPSLAIH
>lpg0940	PSVTSIRSKT DEVLEQ RFNPTPSLSPFKTS
>lpg0944	GTLKEQIGFIKIQ EQNPVEEIE NLSRPNLT
>lpg0945	KFLQKFTFLSCMSPCQ ETAESD KGLNKKPN
>lpg0952	DFHKAKEAIH EGENCV LREKNNILQLVGLE
>lpg1101	TGIKTDSQRAV EEIFSK ALKDLQSPKLAMR
>lpg1109	YRRDRDLNRRAISHLLT EPLENS YKASFSL
>lpg1111	LFFNILLVPENQGH EPEQEN TGEHTHGHSH
>lpg1129	FFSDSPKNP DEHKTE KDKTISPSRNPTKGG
>lpg1152	YQLVRNRFFSSKP EKEELEQE NILQKTIQQ
>lpg1158	EEVAKKIPGDMK DEFTDIE PSSTKPSSTGS
>lpg1166	YDVEALQDTYQ ELEDLYEE YCNPKKELLNF
>lpg1183	FSIWAAKQENLDFGI EEVESV IKQRYVMKS
>lpg1227	LLKGLPGLNNTSSHCT EEFNEQ STPSLYRA
>lpg1290	KTEKTEKTEKTEKT EKTENE QSRNTRGFPI
>lpg1312	QIEKVPEKNVNQNNKQKSL EEGEPP CCTII
>lpg1317	GKNNCNAYRQYFK EIQKEAA DTVSKFDLKK
>lpg1355	EPSMSSTI DEEENI DSEHQIETGTESTMRI
>lpg1483	TGRLGLFSQTRS EKLVEE MKSSVLGIAVGN
>lpg1588	FFATTRAKSAEAV EKAESELE LNQLGSLPK
>lpg1602	VASNNLRFAREVQKLLP DEVTER IVKNILG
>lpg1660	APLFFISPKSR QEENKNE LSSLPSHSVHLH

>lpg1683 QKQIKTDLLDLR EEDKTG LKKPLHGGIKVK
>lpg1687 KLLPHFWNRHGKSSSSYW EEVFTP SCLNNK
>lpg1692 AVWRLVEDAKANII EPEQEQ VPDLRTSGPS
>lpg1701 STHSEESDEGLNDSNDSLQAL EEETVNE IA
>lpg1718 LRESGLHLP EDLLEQ LGIMDSSITLESKFF
>lpg1752 ALRHSR EKQETS FYDIYKGIMRTLGLNSVF
>lpg1797 KSRLQAVVSERNAHK DDTEEV TINNHNSLR
>lpg1884 KLGIMAT EEKESP TPVSAEENTIPTQTLTG
>lpg1953 LEGSFGMLAGVLNQFL EGSTSESE SFDIKL
>lpg1963 TDEKNIFKLSDEKHLS ENKEDI RGKGITNI
>lpg1966 NAFFKLSK ETNKEN VIDQVNQTDVFGFCRN
>lpg2144 AFKDKEKKQIRALIRGMQ EEKIAQ SKCLVC
>lpg2148 PESSPSLFWKNVDKK EQEMTD SSRYKKGPS
>lpg2153 VSTKIGKEPVKQDHTITI DEEESDD IRYGF
>lpg2154 SLLRHVFWCCPSNQKR DDENTD VLEKKAQL
>lpg2155 FWREARKNLSEKSDIDS EKPESE RTTDKRL
>lpg2156 KIGTEPVKLESTHTV DEDERD TYHRESGYF
>lpg2157 DISTKIGREPEHLKFVMV EEDESN KKTIGF
>lpg2176 ASPGSLRIFTNVPV EEDEGL RNRKTEKYKV
>lpg2199 LRLCQYNPKIYLDVATRL EEPAEQ SNTLDF
>lpg2200 YQGGDFKASKEDFTSFKMP EREEPK MGMQL
>lpg2210 KWWYVPNPDYSDRMLN EEDKKL LSMINQAK
>lpg2271 KTSNSTSSVRNK EAEVEE SSQRLYPNLQTV
>lpg2298 SHGIYSTPKGKVTQPKV EVVEDR QTIALVN
>lpg2311 KELNPVHKTT ERTIEQE GPAPESPSTGMRK
>lpg2327 FFAYARPDSAKAV EKAEED LGLRQAASSPK
>lpg2391 PSIAGFFSRSTPVI EEVETE KQEQKSIELK
>lpg2392 ILFTQRHQKDSDNKIIPK ELEESI RTFNSR
>lpg2400 LIFAQKHQTNIEDLNIP DELRES IQTCKPL
>lpg2409 ISSLVHLGLFRDTQIQY EEKEES LHNKYRK
>lpg2424 RLKHRHRHNLFKPQI EEQKINE QNQSLFLG
>lpg2425 LNSWVHIKNQLSQLAGK EVSQER QSLVAPM
>lpg2461 HETVTELYP EFTDPE NPMSGYIYSTFDSPS
>lpg2464 KTSSVSSFEKMV EETRES IKSQERQTIKIK
>lpg2482 FNTVVDLIT EEQQLRHD RFNDNTIGKSISM
>lpg2498 ILWFAPQDKKTIPKPSSQ EELKQN QLSFLN
>lpg2504 CPELGFFGSRRGDL EAKQEN NKLKESILVF
>lpg2508 RFWREHKK ENQSLE KGFDFDRNTSSSQSPL
>lpg2525 SQNLYINQDETIELFNKYT EEKTNN FCSIL
>lpg2526 RSMFSLFSHSTTTEPSPTVK EEESKK SGPQ
>lpg2529 DDVILFVQEMDKIIKQQ ESISEN SSKRAAM
>lpg2556 HASEANIIRKINGFL EDIEYQ NLTNSGFEL
>lpg2577 MEKDLVG EEYKSS LPNPFDISRGPKLPDWI
>lpg2584 KPKDMTSKVDLTAAM EDDNRSE STPTPVNF
>lpg2603 LNLKTSSRKKVMVIFK EAEERI LNSPNVSI
>lpg2793 SKRPCFFKTRSHRLL EEVDTI LHSMAPTSS
>lpg2813 TKGTFKFWKSHGEALSDNI EEVSKQ QPGMK
>lpg2815 HRLFNQKGEKTRTQLFI EELPER INLSSGK

>lpg2829 QNIKGPEPVATPM ETPENE APLVNANITRF
>lpg2830 GFGFFSLNFLTSWLWGT EEKKEK TSSDMTY
>lpg2831 IAEVRREHTDPSPSLQ EKERVG LSTTFGGH
>lpg2832 DIPVMKEICYQTVVNSI ETMQER RTHLSQE
>lpg2862 ITSS EEVERT QSLRTDGLSWMPSEQARLSK
>lpg2884 HTTKVFAQPKDVTP EVVENP TSTVSLTGSK

Supplemental Table 4. BLAST Results for E BLOCK Query

Orf	Gene Name	Features	Original characterization(Ref)
lpg0008	*ravA*		SidC fusion
lpg0012	*cegC1*		SidC fusion; (Altman and Segal, 2008)
lpg0038	*legA1*	Ankyrin Repeat	Eukaryotic homology; (de Felipe *et al.*, 2008)
lpg0096	*ceg4*		SidC fusion;(Burstein *et al.*, 2009)
lpg0126	*cegC2*	lpg1120 paralog	(Altman and Segal, 2008)
lpg0135	*sdhB*		(Laguna *et al.*, 2006)
lpg0209		near *ceg6*	E BLOCK-BLAST
lpg0279		near *legG2*	E BLOCK-BLAST
lpg0294			E BLOCK-BLAST
lpg0403	*legA7*	ankyrin repeat	(de Felipe *et al.*, 2008)
lpg0437	*ceg14*		(Altman and Segal, 2008)
lpg0550			E BLOCK-BLAST
lpg0563			E BLOCK-BLAST
lpg0634			E BLOCK-BLAST
lpg0645		Near *wipB*	E BLOCK-BLAST
lpg0717			E BLOCK-BLAST
lpg0921			E BLOCK-BLAST
lpg1144		Genbank:eukaryotic	E BLOCK-BLAST
lpg1152	*ravP*		SidC fusion
lpg1166	*ravR*		SidC fusion

Supplemental Table 4. BLAST Results for E BLOCK Query, cont

Orf	Gene Name	Features	Original characterization(Ref)
lpg1183	*ravS*		SidC fusion
lpg1377			E BLOCK-BLAST
lpg1663			E BLOCK-BLAST
lpg1683	*ravZ*		SidC fusion
lpg1701	*legC3*	Coiled-coil	Eukaryotic homology (de Felipe *et al.*, 2008)
lpg1798		RhoGAP domain	E BLOCK-BLAST
lpg1925			SidC fusion
lpg1959		Next to *legL5*	E BLOCK-BLAST
lpg2073			E BLOCK-BLAST
lpg2160		lpg2638 paralog	E BLOCK-BLAST
lpg2207		Next to *wipC*	E BLOCK-BLAST
lpg2210			E BLOCK-BLAST
lpg2266			E BLOCK-BLAST
lpg2370			E BLOCK-BLAST
lpg2391	*sdbC*	lipase homology	Cre-lox; (Luo and Isberg, 2004)
lpg2392	*legL6*	leu-rich repeat	SidC fusion; (de Felipe *et al.*, 2008)
lpg2395			E BLOCK-BLAST
lpg2409	*ceg29*		pmrA regulated; (Altman and Segal, 2008)
lpg2420			E BLOCK-BLAST
lpg2455		next to *legA15*	E BLOCK-BLAST

Supplemental Table 4. BLAST Results for E BLOCK Query, cont

Orf	Gene Name	Features	Original characterization(Ref)
lpg2465	*sidD*	next to *sidM*	Cre-lox; (Luo and Isberg, 2004)
lpg2523			E BLOCK-BLAST
lpg2527			E BLOCK-BLAST
lpg2552			E BLOCK-BLAST
lpg2584	*sidF*	Death inhibitor	Cre-lox
lpg2603	*sdmB*	*sidM* paralog	SidC fusion ; (Burstein *et al.*, 2009)
lpg2638		lpg2160 paralog	E BLOCK-BLAST
lpg2678			E BLOCK-BLAST
lpg2793	*lepA*	Coiled-coil	(Chen *et al.*, 2004)
lpg2806		neurofilament like	E BLOCK-BLAST
lpg2826	*ceg34*		pmrA regulated; (Altman and Segal, 2008)
lpg2830	*legU2*	Ubox containing	Eukaryotic homology; (de Felipe *et al.*, 2008)
lpg2874		myosin-like	E BLOCK-Blast
lpg2884	*mavP*		SidC fusion
lpg2907			E BLOCK-BLAST

Shown are orfs encoding putative proteins that have C termini showing sequence similarity to Glu-rich motifs observed in translocated substrates. Orfs were identified by performing 8 Blast queries versus the *L. pneumophila* philadelphia 1 genome using the following sequences as queries: `EExEENxNS; EExETNS; EEVETNS; ExSEKMk; EEEEQEKN; EKSxDLqn; EKEEDKxT; EDxETxNST.`

References.

Altman, E. and Segal, G. (2008) The response regulator CpxR directly regulates expression of several *Legionella pneumophila icm/dot* components as well as new translocated substrates. *J Bacteriol*. **190**: 1985-1996.

Burstein, D., Zusman, T., Degtyar, E., Viner, R., Segal, G. and Pupko, T. (2009) Genome-scale identification of *Legionella pneumophila* effectors using a machine learning approach. *PLoS Pathog*. **5**: e1000508.

Chen, J., de Felipe, K.S., Clarke, M., Lu, H., Anderson, O.R., Segal, G. and Shuman, H.A. (2004) *Legionella* effectors that promote nonlytic release from protozoa. *Science*. **303**: 1358-1361.

de Felipe, K.S., Glover, R.T., Charpentier, X., Anderson, O.R., Reyes, M., Pericone, C.D. and Shuman, H.A. (2008) Legionella eukaryotic-like type IV substrates interfere with organelle trafficking. *PLoS Pathog*. **4**: e1000117.

Laguna, R.K., Creasey, E.A., Li, Z., Valtz, N. and Isberg, R.R. (2006) A *Legionella pneumophila*-translocated substrate that is required for growth within macrophages and protection from host cell death. *Proc Natl Acad Sci U S A*. **103**: 18745-18750.

Luo, Z.Q. and Isberg, R.R. (2004) Multiple substrates of the *Legionella pneumophila* Dot/Icm system identified by interbacterial protein transfer. *Proc Natl Acad Sci U S A*. **101**: 841-846.

Supplemental Table 5: C termini used in Fig. 4A

lpg number	C terminal sequence	Site
>lpg1701	STHSEESDEGLNDSNDSLQAL EEETVNE IA	9
>lpg0227	HVFGKAQTGLESDEALAAQLQ EAELRR ILY	9
>lpg0234	DELLNEFGKQAPRTEMIKTV EEKQGT LLRL	10
>lpg2526	RSMFSLFSHSTTTEPSPTVK EEESKK SGPQ	10
>lpg0012	DGLKKHSILAAKQTHSAIQ EEEEVR LNLGV	11
>lpg0160	LFSFWCEKNESFVESRKII EEHRIC RLNNL	11
>lpg1312	QIEKVPEKNVNQNNKQKSL EEGEPP CCTII	11
>lpg2200	YQGGDFKASKEDFTSFKMP EREEPK MGMQL	11
>lpg2525	SQNLYINQDETIELFNKYT EEKTNN FCSIL	11
>lpg2813	TKGTFKFWKSHGEALSDNI EEVSKQ QPGMK	11
>lpg0135	KEELHTIKNTDGASPRKG EGQEGE IEFTKL	12
>lpg0436	FMTGGEIPPSRKVPESRF EEAGID TPKNAL	12
>lpg1687	KLLPHFWNRHGKSSSSYW EEVFTP SCLNNK	12
>lpg2144	AFKDKEKKQIRALIRGMQ EEKIAQ SKCLVC	12
>lpg2153	VSTKIGKEPVKQDHTITI DEEESD DIRYGF	12
>lpg2157	DISTKIGREPEHLKFVMV EEDESN KKTIGF	12
>lpg2199	LRLCQYNPKIYLDVATRL EEPAEQ SNTLDF	12
>lpg2392	ILFTQRHQKDSDNKIIPK ELEESI RTFNSR	12
>lpg2498	ILWFAPQDKKTIPKPSSQ EELKQN QLSFLN	12
>lpg0196	KFQDTVKEKSEKAVREQ DESEEN RIGLTIS	13
>lpg0246	YKYDILDLIKRVHKDKF EEMEDE NLIRMSI	13
>lpg1109	YRRDRDLNRRAISHLLT EPLENS YKASFSL	13
>lpg1602	VASNNLRFAREVQKLLP DEVTER IVKNILG	13
>lpg2155	FWREARKNLSEKSDIDS EKPESE RTTDKRL	13
>lpg2298	SHGIYSTPKGKVTQPKV EVVEDR QTIALVN	13
>lpg2400	LIFAQKHQTNIEDLNIP DELRES IQTCKPL	13
>lpg2409	ISSLVHLGLFRDTQIQY EEKEES LHNKYRK	13
>lpg2425	LNSWVHIKNQLSQLAGK EVSQER QSLVAPM	13
>lpg2529	DDVILFVQEMDKIIKQQ ESISEN SSKRAAM	13
>lpg2815	HRLFNQKGEKTRTQLFI EELPER INLSSGK	13
>lpg2830	GFGFFSLNFLTSWLWGT EEKKEK TSSDMTY	13
>lpg2832	DIPVMKEICYQTVVNSI ETMQER RTHLSQE	13
>lpg0945	KFLQKFTFLSCMSPCQ ETAESD KGLNKKPN	14
>lpg1227	LLKGLPGLNNTSSHCT EEFNEQ STPSLYRA	14
>lpg1963	TDEKNIFKLSDEKHLS ENKEDI RGKGITNI	14
>lpg2154	SLLRHVFWCCPSNQKR DDENTD VLEKKAQL	14
>lpg2210	KWWYVPNPDYSDRMLN EEDKKL LSMINQAK	14
>lpg2603	LNLKTSSRKKVMVIFK EAEERI LNSPNVSI	14
>lpg2831	IAEVRREHTDPSPSLQ EKERVG LSTTFGGH	14
>lpg1953	LEGSFGMLAGVLNQFL EGSTSESE SFDIKL	14
>lpg0059	KLFQPVNKTALRPND EETDEL SCKPVLLQR	15
>lpg0422	LTWSYVSVLRAIHLR EQLENRI KTTGWNTY	15
>lpg0483	RIVLNEMFNPIPRVH EDIESQ QSSPRVVLK	15
>lpg1183	FSIWAAKQENLDFGI EEVESV IKQRYVMKS	15
>lpg1797	KSRLQAVVSERNAHK DDTEEV TINNHNSLR	15
>lpg2148	PESSPSLFWKNVDKK EQEMTD SSRYKKGPS	15
>lpg2156	KIGTEPVKLESTHTV DEDERD TYHRESGYF	15
>lpg2424	RLKHRHRHNLFKPQI EEQKINE QNQSLFLG	15
>lpg2556	HASEANIIRKINGFL EDIEYQ NLTNSGFEL	15
>lpg2584	KPKDMTSKVDLTAAM EDDNRSE STPTPVNF	15
>lpg2793	SKRPCFFKTRSHRLL EEVDTI LHSMAPTSS	15
>lpg0195	AKIKTKDQVIIRGIN EPEVAE QISKLSKKM	15
>lpg0518	TRLGTTWVSACLKP EVTETE HENTTAYQFN	16

>lpg1111	LFFNILLVPENQGH	EPEQEN	TGEHTHGHSH	16
>lpg1290	KTEKTEKTEKTEKT	EKTENE	QSRNTRGFPI	16
>lpg1692	AVWRLVEDAKANII	EPEQEQ	VPDLRTSGPS	16
>lpg2176	ASPGSLRIFTNVPV	EEDEGL	RNRKTEKYKV	16
>lpg2391	PSIAGFFSRSTPVI	EEVETE	KQEQKSIELK	16
>lpg2504	CPELGFFGSRRGDL	EAKQEN	NKLKESILVF	16
>lpg2884	HTTKVFAQPKDVTP	EVVENP	TSTVSLTGSK	16
>lpg0096	PSILSRRTPTKGK	EEQCQEE	YVLTNPLHNS	17
>lpg0171	FAQPSQSAAQQNL	EEENLS	ADPKACQCEPL	17
>lpg0944	GTLKEQIGFIKIQ	EQNPVEE	IENLSRPNLT	17
>lpg1152	YQLVRNRFFSSKP	EKEELE	QENILQKTIQQ	17
>lpg1317	GKNNCNAYRQYFK	EIQKEAA	DTVSKFDLKK	17
>lpg1588	FFATTRAKSAEAV	EKAESE	LELNQLGSLPK	17
>lpg2327	FFAYARPDSAKAV	EKAEED	LGLRQAASSPK	17
>lpg2829	QNIKGPEPVATPM	ETPENE	APLVNANITRF	17
>lpg0898	ASQTKSTEQKTL	EIEHLP	TEEIQPPSLAIH	18
>lpg1158	EEVAKKIPGDMK	DEFTDIE	PSSTKPSSTGS	18
>lpg1483	TGRLGLFSQTRS	EKLVEE	MKSSVLGIAVGN	18
>lpg1683	QKQIKTDLLDLR	EEDKTG	LKKPLHGGIKVK	18
>lpg2271	KTSNSTSSVRNK	EAEVEE	SSQRLYPNLQTV	18
>lpg2464	KTSSVSSFEKMV	EETRES	IKSQERQTIKIK	18
>lpg0008	NSQMEPIVSDS	EEEEEK	QNCWTSFCGLFGK	19
>lpg1101	TGIKTDSQRAV	EEIFSK	ALKDLQSPKLAMR	19
>lpg1166	YDVEALQDTYQ	ELEDLYEE	YCNPKKELLNF	19
>lpg1660	APLFFISPKSR	QEENKNE	LSSLPSHSVHLH	19
>lpg0126	GRWRLFPVPT	ESEKIV	KDFSLSMQPLSVSA	20
>lpg0294	QEPKALETKR	EEIRQE	IESGAEAPTTQSIR	20
>lpg0390	FLKELNAKIK	ERKERP	KDEPHTTPVEKKNL	20
>lpg0940	PSVTSIRSKT	DEVLEQ	RFNPTPSLSPFKTS	20
>lpg2311	KELNPVHKTT	ERTIEQE	GPAPESPSTGMRK	20
>lpg0952	DFHKAKEAIH	EGENCV	LREKNNILQLVGLE	20
>lpg1129	FFSDSPKNP	DEHKTE	KDKTISPSRNPTKGG	21
>lpg1718	LRESGLHLP	EDLLEQ	LGIMDSSITLESKFF	21
>lpg2461	HETVTELYP	EFTDPE	NPMSGYIYSTFDSPS	21
>lpg2482	FNTVVDLIT	EEQQLR	HDRFNDNTIGKSISM	21
>lpg0016	DQELPAFK	ETHELE	ILYQLILSWNYLCADG	22
>lpg0634	FKKAPSMS	EEDEED	DLKDQNTAEETSKPTV	22
>lpg1355	EPSMSSTI	DEEENI	DSEHQIETGTESTMRI	22
>lpg1966	NAFFKLSK	ETNKEN	VIDQVNQTDVFGFCRN	22
>lpg2508	RFWREHKK	ENQSLE	KGFDFDRNTSSSQSPL	22
>lpg1884	KLGIMAT	EEKESP	TPVSAEENTIPTQTLTG	23
>lpg2577	MEKDLVG	EEYKSS	LPNPFDISRGPKLPDWI	23
>lpg1752	ALRHSR	EKQETS	FYDIYKGIMRTLGLNSVF	24
>lpg2147	PSEQLA	EIKAESE	TAQKAWDEQYGLVLRDK	24
>lpg0693	ILSYG	EEAFSNPGEE	LQTDSVMQPPMVFNI	25
>lpg2862	ITSS	EEVERT	QSLRTDGLSWMPSEQARLSK	26

Supplemental Table 6: Carboxyl termini of translocation competent fusions to SidC that have identifiable E block

Gene	C terminal sequence	Distance from C terminus
lpg0008	NSQMEPIVSDS EEEEEK QNCWTSFCGLFGK	19
lpg0012	DGLKKHSILAAKQTHSAIQ EEEEVR LNLGV	11
lpg0030	TSHPASRYKQ EDVVNQ LDQWIAQNSTVNFLK	21
lpg0059	KLFQPVNKTALRPND EETDEL SCKPVLLQR	15
lpg0086	PDSPSIPHSKS ESALNQ LIPSQNST I LGLA	19
lpg0096	PSILSRRTPTKGK EEQCQEE YVLTNPLHNS	17
lpg0160	LFSFWCEKNESFVESRKII EEHRIC RLNNL	11
lpg0191	**IISLSTDN EVLAAE DAISLVQPN L GKPQLK**	22
lpg0195	AKIKTKDQVIIRGIN EPEVAE QISKLSKKM	15
lpg0196	KFQDTVKEKSEKAVREQ DESEEN RIGLTIS	13
lpg0210	TSALQDSLIVNNSDIYNVA EDNTMD WISNCPQP	14
lpg0401	FLTSTKTTTKPLSRSH DDEKKT GMDG H GFH	14
lpg0439	GFFHKMKSASDGITTL EKTLALE DDKINPFA	15
lpg0518	TRLGTTWVSACLKP EVTETE HENTTAYQFN	16
lpg0634	FKKAPSMS EEDEED DLKDQNTAEETSKPTV	22
lpg0696	FGENQNKLYKAFHKKIELQLL DSEIE NKNEL	10
lpg0733	NIATLFGNRAQNM EQHEGQ HSKPNDSLSIRSI	19
lpg0898	ASQTKSTEQKTL EIEHLP TEEIQPPSLAIH	18
lpg0926	DVSDLPMVKSS ETTDTQ PNQS F SMSNGHGD	19
lpg0944	GTLKEQIGFIKIQ EQNPVEE IENLSRPNLT	17
lpg0968	ITKIKLKL EDTLGS LQK V QEESLSL	17
lpg0969	PVTITPEVKILFYQLVK EHFHSPETE IKLD I	14
lpg1109	YRRDRDLNRRAISHLLT EPLENS YKASFSL	13
lpg1111	LFFNILLVPENQGH EPEQEN TGEHTHGHSH	16
lpg1120	GLFSKPTNINPNQ ELDIDL NQKLN L ETKTKN	18
lpg1121	TGVLGTTMKPLL EDEHIGNE LKDLLSIKTDNVNI	22
lpg1129	FFSDSPKNP DEHKTE KDKTISPSRNPTKGG	21
lpg1148	HSPTLFFTLATQ ERNTND MADELSSSGPKT	18
lpg1152	YQLVRNRFFSSKP EKEELE QENILQKTIQQ	17
lpg1154	EKGKIKLYLAKIMDIQ EILTSKNGE F VPGL	14
lpg1158	EEVAKKIPGDMK DEFTDIE PSSTKPSSTGS	18
lpg1166	YDVEALQDTYQ ELEDLYEE YCNPKKELLNF	19
lpg1183	FSIWAAKQENLDFGI EEVESV IKQRYVMKS	15
lpg1290	KTEKTEKTEKTEKT EKTENE QSRNTRGFPI	16
lpg1316	ESFFKEMQRNAKNQL ESSKPQDD TPVLRKSY	16
lpg1317	GKNNCNAYRQYFK EIQKEAA DTVSKFDLKK	17
lpg1551	KKDDQHGLNHFLT EYDPD LQFRPSRP	13
lpg1588	FFATTRAKSAEAV EKAESE LELNQLGSLPK	17
lpg1602	VASNNLRFAREVQKLLP DEVTER IVKNILG	13
lpg1683	QKQIKTDLLDLR EEDKTG LKKPLHGGIKVK	18
lpg1687	KLLPHFWNRHGKSSSSYW EEVFTP SCLNNK	12
lpg1752	ALRHSR EKQETS FYDIYKGIMRTLGLNSVF	24
lpg1797	KSRLQAVVSERNAHK DDTEEV TINNHNSLR	15
lpg1851	QKPKSSQGGLFSVPSGK EKVTDTHE VANHRL	14
lpg1949	QKLPLAVKGEM ESARE IYTTVDREKSNLQSHKIN	23

lpg1963	TDEKNIFKLS DEKHLSENKEDI RGKGITNI	14
lpg2147	LPSEQLA EIKAESE TAQKAWDEQYGLVLRDK	24
lpg2199	IFLRLCQYNPKIYLDVATRL EEPAEQ SNTLD F	12
lpg2311	KELNPVHKTT ERTIEQE GPAPESPSTGMRK	20
lpg2351	QMYLS EEYKNR HQ H PNKDPDLKTNESLKPGL	26
lpg2424	RLKHRHRHNLFKPQI EEQKINE QNQSLFLG	15
lpg2425	LNSWVHIKNQLSQLAGK EVSQER QSLVAPM	13
lpg2498	ILWFAPQDKKTIPKPSSQ EELKQN QLSFLN	12
lpg2504	CPELGFFGSRRGDL EAKQEN NKLKESILVF	16
lpg2525	SQNLYINQDETIELFNKYT EEKTNN FCSIL	11
lpg2526	RSMFSLFSHSTTTEPSPTVK EEESKK SGPQ	10
lpg2529	DDVILFVQEMDKIIKQQ ESISEN SSKRAAM	13
lpg2577	MEKDLVG EEYKSS LPNPFDISRGPKLPDWI	23
lpg2603	LNLKTSSRKKVMVIFK EAEERI LNSPNVSI	14
lpg2793	SKRPCFFKTRSHRLL EEVDTI LHSMAPTSS	15
lpg2815	HRLFNQKGEKTRTQLFI EELPER INLSSGK	13
lpg2879	ISPDSLFSTRRRRVNPDQ EKVAITE LSMTK	12
lpg2884	HTTKVFAQPKDVTP EVVENP TSTVSLTGSK	16
lpg2975	EHRADYFKEVKNYA EPISHRQE TTLNSNV TSLVV	20

Supplemental Table 7: ExxE motif in E Block proteins

I. Members of original BLAST search pool

lpg number	Motif	ExxE	EExxE	ExxxE
>lpg0008 NSQMEPIVSDS EEEEEK QNCWTSFCGLFGK	EExxE		1	
>lpg0012 DGLKKHSILAAKQTHSAIQ EeeEVR LNLGV	ExxE	1		
>lpg0016 DQELPAFK EthELE ILYQLILSWNYLCADG	ExxE	1		
>lpg0059 KLFQPVNKTALRPND EETFEL SCKPVLLQR	EExxE		1	
>lpg0096 PSILSRRTPTKGK EEQCQEE YVLTNPLHNS	EExxxE			1
>lpg0126 GRWRLFPVPT ESEKIV KDFSLSMQPLSVSA				
>lpg0135 KEELHTIKNTDGASPRKG EGQEGE IEFTKL	ExxE	1		
>lpg0160 LFSFWCEKNESFVESRKII EEHRIC RLNNL				
>lpg0171 FAQPSQSAAQQNL EEENLS ADPKACQCEPL				
>lpg0195 AKIKTKDQVIIRGIN EPEVAE QISKLSKKM	ExxE	1		
>lpg0196 KFQDTVKEKSEKAVREQ DESEEN RIGLTIS	ExxE	1		
>lpg0227 HVFGKAQTGLESDEALAAQLQ EAELRR ILY				
>lpg0234 DELLNEFGKQAPRTEMIKTV EEKQGT LLRL				
>lpg0246 YKYDILDLIKRVHKDKF EEMEDE NLIRMSI	EExxD		1	
>lpg0294 QEPKALETKR EEIRQE IESGAEAPTTQSIR	EExxxE			1
>lpg0390 FLKELNAKIK ERKERP KDEPHTTPVEKKNL	ExxE	1		
>lpg0422 LTWSYVSVLRAIHLR EQLENRI KTTGWNTY	ExxE	1		
>lpg0436 FMTGGEIPPSRKVPESRF EEAGID TPKNAL				
>lpg0483 RIVLNEMFNPIPRVH EDIESQ QSSPRVVLK	ExxE	1		
>lpg0518 TRLGTTWVSACLKP EVTETE HENTTAYQFN	ExxE	1		
>lpg0634 FKKAPSMS EEDEED DLKDQNTAEETSKPTV	EExxE		1	
>lpg0693 ILSYG EEAFSNPGEE LQTDSVMQPPMVFNI				
>lpg0898 ASQTKSTEQKTL EIEHLP TEEIQPPSLAIH				
>lpg0940 PSVTSIRSKT DEVLEQ RFNPTPSLSPFKTS	ExxE	1		
>lpg0944 GTLKEQIGFIKIQ EQNPVEE IENLSRPNLT	ExxE	1		
>lpg0945 KFLQKFTFLSCMSPCQ ETAESD KGLNKKPN	ExxE	1		
>lpg0952 DFHKAKEAIH EGENCV LREKNNILQLVGLE				
>lpg1101 TGIKTDSQRAV EEIFSK ALKDLQSPKLAMR				
>lpg1109 YRRDRDLNRRAISHLLT EPLENS YKASFSL	ExxE	1		
>lpg1111 LFFNILLVPENQGH EPEQEN TGEHTHGHSH	ExxE	1		
>lpg1129 FFSDSPKNP DEHKTE KDKTISPSRNPTKGG	ExxxE			1
>lpg1152 YQLVRNRFFSSKP EKEELE QENILQKTIQQ	ExxE	1		
>lpg1158 EEVAKKIPGDMK DEFTDIE PSSTKPSSTGS	ExxD	1		
>lpg1166 YDVEALQDTYQ ELEDLYEE YCNPKKELLNF	ExxD	1		
>lpg1183 FSIWAAKQENLDFGI EEVESV IKQRYVMKS	ExxE	1		
>lpg1227 LLKGLPGLNNTSSHCT EEFNEQ STPSLYRA	EExxE		1	
>lpg1290 KTEKTEKTEKTEKT EKTENE QSRNTRGFPI	ExxE	1		
>lpg1312 QIEKVPEKNVNQNNKQKSL EEGEPP CCTII	ExxE	1		
>lpg1317 GKNNCNAYRQYFK EIQKEAA DTVSKFDLKK	ExxxE			1
>lpg1355 EPSMSSTI DEEENI DSEHQIETGTESTMRI	DExxE		1	
>lpg1483 TGRLGLFSQTRS EKLVEE MKSSVLGIAVGN	ExxxE			1
>lpg1588 FFATTRAKSAEAV EKAESE LELNQLGSLPK	ExxE	1		
>lpg1602 VASNNLRFAREVQKLLP DEVTER IVKNILG	DExxE		1	
>lpg1660 APLFFISPKSR QEENKNE LSSLPSHSVHLH	EExxxE			1
>lpg1683 QKQIKTDLLDLR EEDKTG LKKPLHGGIKVK				

Supplemental Table 7: ExxE motif in E Block proteins

I . Members of original BLAST search pool, continued

lpg number	Motif	ExxE	EExxE	ExxxE
>lpg1687 KLLPHFWNRHGKSSSSYW EEVFTP SCLNNK				
>lpg1692 AVWRLVEDAKANII EPEQEQ VPDLRTSGPS	ExxxE			1
>lpg1701 STHSEESDEGLNDSNDSLQAL EEETVNE IA	EExxxE			1
>lpg1718 LRESGLHLP EDLLEQ LGIMDSSITLESKFF	ExxxE			1
>lpg1752 ALRHSR EKQETS FYDIYKGIMRTLGLNSVF	ExxE	1		
>lpg1797 KSRLQAVVSERNAHK DDTEEV TINNHNSLR	DDxxE		1	
>lpg1884 KLGIMAT EEKESP TPVSAEENTIPTQTLTG	ExxE	1		
>lpg1953 LEGSFGMLAGVLNQFL EGSTSESE SFDIKL				
>lpg1963 TDEKNIFKLSDEKHLS ENKEDI RGKGITNI	ExxE	1		
>lpg1966 NAFFKLSK ETNKEN VIDQVNQTDVFGFCRN	ExxxE			1
>lpg2144 AFKDKEKKQIRALIRGMQ EEKIAQ SKCLVC				
>lpg2148 PESSPSLFWKNVDKK EQEMTD SSRYKKGPS				
>lpg2153 VSTKIGKEPVKQDHTITI DEEESD DIRYGF	EExxD		1	
>lpg2154 SLLRHVFWCCPSNQKR DDENTD VLEKKAQL	DExxD		1	
>lpg2155 FWREARKNLSEKSDIDS EKPESE RTTDKRL	ExxE	1		
>lpg2156 KIGTEPVKLESTHTV DEDERD TYHRESGYF	EDxxD		1	
>lpg2157 DISTKIGREPEHLKFVMV EEDESN KKTIGF	ExxE	1		
>lpg2176 ASPGSLRIFTNVPV EEDEGL RNRKTEKYKV	ExxE	1		
>lpg2199 LRLCQYNPKIYLDVATRL EEPAEQ SNTLDF	EExxE		1	
>lpg2200 YQGGDFKASKEDFTSFKMP EREEPK MGMQL	ExxE	1		
>lpg2210 KWWYVPNPDYSDRMLN EEDKKL LSMINQAK				
>lpg2271 KTSNSTSSVRNK EAEVEE SSQRLYPNLQTV	ExxE	1		
>lpg2298 SHGIYSTPKGKVTQPKV EVVEDR QTIALVN	ExxE	1		
>lpg2311 KELNPVHKTT ERTIEQE GPAPESPSTGMRK	ExxxE			1
>lpg2327 FFAYARPDSAKAV EKAEED LGLRQAASSPK	ExxE	1		
>lpg2391 PSIAGFFSRSTPVI EEVETE KQEQKSIELK	ExxE	1		
>lpg2392 ILFTQRHQKDSDNKIIPK ELEESI RTFNSR	ExxE	1		
>lpg2400 LIFAQKHQTNIEDLNIP DELRES IQTCKPL	DExxE		1	
>lpg2409 ISSLVHLGLFRDTQIQY EEKEES LHNKYRK	EExxE		1	
>lpg2424 RLKHRHRHNLFKPQI EEQKINE QNQSLFLG	ExxxE			1
>lpg2425 LNSWVHIKNQLSQLAGK EVSQER QSLVAPM	ExxxE			1
>lpg2461 HETVTELYP EFTDPE NPMSGYIYSTFDSPS	ExxD	1		
>lpg2464 KTSSVSSFEKMV EETRES IKSQERQTIKIK	EExxE		1	
>lpg2482 FNTVVDLIT EEQQLR HDRFNDNTIGKSISM				
>lpg2498 ILWFAPQDKKTIPKPSSQ EELKQN QLSFLN				
>lpg2504 CPELGFFGSRRGDL EAKQEN NKLKESILVF	ExxxE			1
>lpg2508 RFWREHKK ENQSLE KGFDFDRNTSSSQSPL				
>lpg2525 SQNLYINQDETIELFNKYT EEKTNN FCSIL				
>lpg2526 RSMFSLFSHSTTTEPSPTVK EEESKK SGPQ				
>lpg2529 DDVILFVQEMDKIIKQQ ESISEN SSKRAAM	ExxxE			1
>lpg2556 HASEANIIRKINGFL EDIEYQ NLTNSGFEL	ExxE	1		
>lpg2577 MEKDLVG EEYKSS LPNPFDISRGPKLPDWI				
>lpg2584 KPKDMTSKVDLTAAM EDDNRSE STPTPVNF	DDxxxE		1	
>lpg2603 LNLKTSSRKKVMVIFK EAEERI LNSPNVSI	ExxE	1		1
>lpg2793 SKRPCFFKTRSHRLL EEVDTI LHSMAPTSS	ExxD	1		

Supplemental Table 7: ExxE motif in E Block proteins
I . Members of original BLAST search pool, continued

lpg number	Motif	ExxE	EExxE	ExxxE
>lpg2813 TKGTFKFWKSHGEALSDNI EEVSKQ QPGMK	EExxE		1	
>lpg2815 HRLFNQKGEKTRTQLFI EELPER INLSSGK	ExxE	1		
>lpg2829 QNIKGPEPVATPM ETPENE APLVNANITRF	ExxE	1		
>lpg2830 GFGFFSLNFLTSWLWGT EEKKEK TSSDMTY	EExxE		1	
>lpg2831 IAEVRREHTDPSPSLQ EKERVG LSTTFGGH	ExxxE			1
>lpg2832 DIPVMKEICYQTVVNSI ETMQER RTHLSQE	ExxxE			1
>lpg2862 ITSS EEVERT QSLRTDGLSWMPSEQARLSK	ExxE	1		
>lpg2884 HTTKVFAQPKDVTP EVVENP TSTVSLTGSK	ExxE	1		
	**# motifs**	**41**	**18**	**18**

II. Proteins identified by BLAST searching for E BLOCK

lpg number	Motif	ExxE	EExxE	ExxxE
>lpg0294 QEPKALETKR EEIRQEIE SGAEAPTTQSIR	EExxxE		1	
>lpg0437 KSNSFFSKK DEESGD KKNDHTPKDGTVYGC	EExxD		1	
>lpg0563 DYMQKSFQIIEKAMLSFVQ EAKKTTAE VKK				
>lpg0921 NTAKAVK EAEQVIS LLQDQDSMQLVVYNGT				
>lpg1144 KEEAEFSSSESEN EEKEEENEE SSRFTM	EExxE		1	
>lpg1663 AEPKVVSEDKA ESEEENEDEE SRNSASV	EExxE		1	
>lpg1670 ILDEFTVEVKDMP EEEKLENE VAPTLSI	EExxE		1	
>lpg1798 SSAYLI EEQEEGE VTENEYSGLDSAKLT	EExxE		1	
>lpg2073 IKEDSLAITY EINEAIH LLSEEIDKNSR	ExxE	1		
>lpg2160 PSSEEEFDDI EESEWEQ PELSRSVASPM	ExxE	1		
>lpg2395 EQ EELETIEAMKE KDDARFNEIRQTMYA	ExxxE			1
>lpg2409 LVHLGLFRDTQIQY EEKEES LHNK Y RK	EExxE			
>lpg2420 GFYKRKGGRVIC EGRYND SYPTEVMRLRF				
>lpg2455 YSIFGGKDNRKVMQ EKTDSLTS YDIQPHH	ExxD	1		
>lpg2523 SALQHAPHQQYVVKH EEEESLD SDMWVK	ExxE,EExx	1	1	
>lpg2527 KPSKNQDSEVKKTL DSEQLEE VQRQDDKS	ExxE	1		
>lpg2552 DVGLS EERTHKPN SNPTLESRAVLTSPSP				
>lpg2638 IIGRTYCEDHV ELEDNEG KIALSPQPLA	ExxE	1		
>lpg2678 GIAKREEIKQLHN ELEDLAK TNSTMCWI	ExxD	1		
>lpg2806 SRKLSAKESDD ENGESES DIEHPDVPKI	ExxE	1		
>lpg2826 KVRITTELVQ EEVGQEFDE NHTSLYRNN	EExxxE			1
>lpg2907 EEFESTCAI DDLEEYANS CLSSKSVRL	DDxxE		1	
	**# motifs**	**8**	**6**	**2**

	ExxE	EExxE	ExxxE
**TOTAL =**	**49**	**24**	**20**
**Total with ≥1 D**			
**18**			
**Total ExxE, no D**			
**55**			
